# A complex network of interactions governs DNA methylation at telomeric regions

**DOI:** 10.1093/nar/gkac012

**Published:** 2022-01-21

**Authors:** Colin Farrell, María I Vaquero-Sedas, María D Cubiles, Michael Thompson, Alejandro Vega-Vaquero, Matteo Pellegrini, Miguel A Vega-Palas

**Affiliations:** Department of Molecular, Cell and Developmental Biology, University of California, Los Angeles, CA90095, USA; Instituto de Bioquímica Vegetal y Fotosíntesis, CSIC-Universidad de Sevilla, IBVF (CSIC-US), Seville, E41092, Spain; Departamento de Estadística e Investigación Operativa, Facultad de Matemáticas, Universidad de Sevilla, Seville, E41012, Spain; Department of Molecular, Cell and Developmental Biology, University of California, Los Angeles, CA90095, USA; Escuela Técnica Superior de Ingeniería Informática, Universidad de Sevilla, Seville, E41012, Spain; Department of Molecular, Cell and Developmental Biology, University of California, Los Angeles, CA90095, USA; Institute of Genomics and Proteomics, Los Angeles, CA90095, USA; Instituto de Bioquímica Vegetal y Fotosíntesis, CSIC-Universidad de Sevilla, IBVF (CSIC-US), Seville, E41092, Spain

## Abstract

DNA methylation modulates telomere function. In *Arabidopsis thaliana*, telomeric regions have a bimodal chromatin organization with unmethylated telomeres and methylated subtelomeres. To gain insight into this organization we have generated TAIR10-Tel, a modified version of the Arabidopsis reference genome with additional sequences at most chromosome ends. TAIR10-Tel has allowed us to analyse DNA methylation at nucleotide resolution level in telomeric regions. We have analysed the wild-type strain and mutants that encode inactive versions of all currently known relevant methyltransferases involved in cytosine methylation. These analyses have revealed that subtelomeric DNA methylation extends 1 to 2 kbp from Interstitial Telomeric Sequences (ITSs) that abut or are very near to telomeres. However, DNA methylation drops at the telomeric side of the telomere-subtelomere boundaries and disappears at the inner part of telomeres. We present a comprehensive and integrative model for subtelomeric DNA methylation that should help to decipher the mechanisms that govern the epigenetic regulation of telomeres. This model involves a complex network of interactions between methyltransferases and subtelomeric DNA sequences.

## INTRODUCTION

Telomeres together with telomerase guarantee the replication of chromosome ends, prevent genome instability and influence relevant biological processes like the proliferative capacity of stem cells, illness, aging and cancer. In most eukaryotes, telomeres are composed of short G/C-rich double strand tandem repeats followed by a short stretch of single strand repeats (1). These repeats are also present at internal chromosomal loci, where they have been related to genome instability. However, the function of these Interstitial Telomeric Sequences (ITSs) remains largely unknown (2). The primary DNA sequences of telomeres and ITSs differ. Whereas telomeres are essentially composed of perfect tandem telomeric repeat arrays, ITSs usually contain perfect telomeric repeats interspersed with degenerate telomeric repeats (2). These differences in sequence compositions are thought to influence the chromatin organizations and functional properties of telomeres and ITSs.

The length of telomeres and the chromatin organization of telomeric regions influence telomere functions (3,4). Multiple states of chromatin have been described according to the combinational distribution of several epigenetic modifications such as H3K4, H3K9, H3K27 and H3K36 methylation, different types of histones acetylation and cytosine methylation (5–7). All these chromatin states can be grouped within two major types: euchromatin and heterochromatin. Euchromatin can be transcriptionally active or silenced, in which case it is referred as polycomb chromatin. In turn, heterochromatin is usually silenced although it requires certain level of transcription (3).

Cytosine methylation is important for cell biology and regulates multiple processes in plants and animals, including the homeostasis of telomere length (8–11). However, whereas mammalian DNA methylation is primarily found in the CG context, plants have significant levels of DNA methylation in the CG, CHG and CHH contexts (where H is A, C or T) (8). In plants, CG methylation (CGm) can be found in heterochromatin and in the body of most euchromatic genes other than the shortest ones. In turn, CHGm and CHHm are almost exclusively located in heterochromatic regions. Hence, plant heterochromatin is characterized by the presence of DNA methylation in the three sequence contexts. In addition, plant heterochromatin is labelled with additional epigenetic marks such as H3K9me2 (5,6,8,12).

In Arabidopsis, specific DNA methyltransferases establish and/or maintain the different types of cytosine methylation with the assessment of a plethora of accessory proteins, including chromatin remodelers that allow access to heterochromatin like DDM1 or DRD1 (13–15). Methylation in all sequence contexts is established *de novo* by the RNA-directed DNA Methylation (RdDM) pathway, which relies on the activity of DOMAINS REARRANGED METHYLTRANSFERASE 2 (DRM2). Once established, methylation is maintained by METHYLTRANSFERASE 1 (MET1), CHROMOMETHYLASES 2 and 3 (CMT2 and CMT3) and also DRM2. Whereas MET1 and CMT3 are the major CG and CHG methyltransferases, respectively, CMT2 and DRM2 maintain methylation at CHH sites and, to a lower extent, at CHG sites. These methyltransferases are responsible for most of the Arabidopsis DNA methylation and can be recruited to specific genomic loci through different pathways (8,12). MET1 is thought to be recruited to hemi-methylated CG sites through DNA replication by VARIANT IN METHYLATION proteins 1–3 (VIM1–3) and methylate the newly synthetized CG sites (16–19). In addition, CMT3, CMT2 and DRM2 can associate with the heterochromatic H3K9me2 mark to maintain non-CGm (20–23). Indeed, the histone methyltransferases that maintain H3K9me2, the SU(VAR)3–9 HOMOLOGUES KYP (hereinafter referred as SUVH4), SUVH5 and SUVH6, should be considered part of the non-CGm machinery (16,24–26). CMT3 and CMT2 bind directly to H3K9me2 and, then, methylate DNA. In turn, SUVH4, SUVH5 and SUVH6 bind to methylated cytosines and establish H3K9me2. Thus, these methyltransferases create a positive feedback loop that reinforces heterochromatin spreading and maintenance (8,12). As mentioned above, DRM2 is targeted to heterochromatin through the RdDM pathway. In this pathway, DRM2 requires the upstream action of two specific plant RNA polymerases, POLIV and POLV (16,27,28). POLIV can be recruited to H3K9me2 indirectly through the action of the SAWADEE HOMEODOMAIN HOMOLOG 1 (SHH1) protein. However, POLIV can also be targeted by alternative means, independently of SHH1 (16,21–23). POLV is indirectly recruited to heterochromatin by two inactive histone methyltransferases that bind methylated cytosines, SUVH2 and SUVH9 (29–31). Whereas POLIV transcribes precursors for small interfering RNAs (siRNAs), POLV produces scaffold transcripts that are recognized by these siRNAs bound to ARGONAUTE (AGO) proteins, which, in turn, recruit DRM2 (8,12).

In *Arabidopsis thaliana*, telomeres are not heterochromatic and, consequently, their cytosines remain unmethylated. By contrast, Arabidopsis ITSs are heterochromatic and undergo DNA methylation. Similarly, Arabidopsis subtelomeres also undergo DNA methylation. Therefore, telomeric regions in Arabidopsis have a bimodal chromatin organization with unmethylated telomeres and methylated subtelomeres (32–35). However, the molecular mechanisms that govern this bimodal organization remain largely unknown.

Here, we analysed DNA methylation at Arabidopsis telomeric regions. To that end, we first completed the DNA sequences of most Arabidopsis chromosome ends, generating a modified version of the Arabidopsis reference genome that we denote as TAIR10-Tel. Then, we aligned high-quality previously published Whole Genome Bisulfite Sequencing (WGBS) libraries to TAIR10-Tel. We found that telomeric regions in Arabidopsis contain ITSs that abut or localize very near to telomeres, are enriched in specific types of degenerate telomeric repeats and undergo high levels of methylation. This DNA methylation extends up to about 2 kbp into subtelomeres. However, DNA methylation decreases at the telomeric side of the telomere-subtelomere boundaries and disappear at the inner part of telomeres. Hence, boundaries are transition regions characterized by a shift in genetic and epigenetic organization. Our analyses of subtelomeric DNA methylation extend previously reported WGBS studies. They reveal that a complex network of interactions governs subtelomeric DNA methylation and highlight the relevance that primary DNA sequences and different methyltransferases play in this process.

## MATERIALS AND METHODS

### Identification of new DNA sequences at Arabidopsis chromosome ends

The DNA sequences present at most ends of the five *Arabidopsis thaliana* chromosomes are ill-defined in the currently used version of the Arabidopsis reference genome (TAIR10) (36). Two of these ends, the left telomeric regions of chromosomes 2 and 4 (2L and 4L, respectively), contain long arrays of ribosomal genes and have not been fully assembled. In addition, the sequences of most of the remaining ends do not include telomeres, as can be observed in TAIR10 (36).

We used previously published DNA sequence data to assemble additional sequences to 3L, 5L, 1R and 5R. These data were obtained from different Arabidopsis genome-wide DNA sequencing experiments including SRR1818411, SRR2087601, SRR1168327, SRR1451413, SRR1945757, SRR5626994 and SRR5209711 (37–43). First, we assembled illumina reads from these experiments with unique sequences present at 3L, 5L, 1R and 5R. Then, we assembled these reads with additional reads until they connected with long arrays of perfect telomeric repeats containing 9 tandem repeats or more. According to our previous research, only one Arabidopsis ITS located in the pericentromeric region of chromosome 3 contains >8 perfect tandem telomeric repeats (33,35). Thus, tandem arrays containing >9 perfect telomeric repeats indicate the beginning of telomeres.

### Modification of the Arabidopsis TAIR10 reference genome

To build an extended version of the Arabidopsis TAIR10 genome, which we denote as TAIR10-Tel, we first added the newly identified DNA sequences to 3L, 5L, 1R and 5R. In addition, we also incorporated previously published sequences to 1L and 3R (Supplementary Figure S1) (44,45). Then, we removed perfect telomeric repeats from the very end of the telomeric sequences in order to keep only 10 perfect telomeric repeats. Therefore, telomeres in TAIR10-Tel are represented by 10 perfect telomeric repeats, which extend 70 bp (Supplementary Figures S1 and S2). Keeping only 10 perfect telomeric repeats at the end of the chromosomes allows mapping cytosine methylation at each specific telomere-subtelomere boundary, even if reads mapping to more than one site in the genome are allowed to align. Since WGBS illumina reads are usually >70 bp, telomeric reads arising from inside telomeres and containing only arrays of perfect telomeric repeats should not align to TAIR10-Tel telomeres. Only reads containing subtelomeric and telomeric sequences should align to them. If TAIR10-Tel would contain long arrays of perfect telomeric repeats at telomeres, telomeric reads arising from inside telomeres would be shorter than TAIR10-Tel telomeres and would align to them, which would not allow the faithful analysis of DNA methylation evolution through the telomere-subtelomere boundaries. We envision that, in the future, the study of DNA methylation at telomere-subtelomere boundaries containing long stretches of perfect tandem telomeric repeats will be possible by analysing Nanopore reads, when methods for estimating CHHm from these reads are refined.

Although TAIR10-Tel contains 2R and 4R, we did not include these chromosome ends in the methylation analyses shown here. Whereas both ends contain large amounts of ITSs and are poorly covered after mapping WGBS experiments, 4R does not include the telomere-subtelomere boundary.

### Selection of WGBS experiments and mapping to TAIR10-Tel

We used different sets of WGBS data to analyse cytosine methylation (Supplementary Table S1). In all cases, we selected experiments with high genomic cytosines conversion from the Sequence Read Archive at the National Center from Biotechnology Information. We used three different experiments to analyse subtelomeric DNA methylation in the Arabidopsis wild-type (WT), which allowed us to avoid coverage gaps as much as possible (15,16). These experiments were SRR534177, SRR534193 and SRR771524. Although SRR771524 was performed using DNA from seedlings and SRR534177 and SRR534193 were performed using DNA from leaves, similar results were obtained when both kinds of experiments were analysed independently (data not shown). Whereas SRR534177 and SRR534193 were also used for comparison with the DNA methylation mutants, as they were reported in the same study, SRR771524 was also used to analyse cytosine methylation at the telomere-subtelomere boundaries. DNA methylation mutants included *met1*, *cmt3*, *suvh4/5/6*, *drm1/2* and *cmt2* and were analysed using experiments SRR534239, SRR534209, SRR534253, SRR534222, SRR534223 and SRR869314 (16). These experiments yielded high coverage depth (>20×) and, therefore, allowed accurate analyses of subtelomeric DNA methylation. Since *DRM1* is only expressed in the female gamete, for simplicity, we refer to the *drm1/2* mutant as *drm2* (46). Considering that mapping of cytosine methylation at the telomere-subtelomere boundaries is complicated by the repetitiveness of telomeres and subtelomeric ITSs and by the tendency of telomeric cytosines to remain unconverted after bisulfite treatment (35), experiments with long reads, high coverage and high levels of telomeric cytosines conversion should be selected to analyse the boundaries. We assessed the levels of telomeric cytosines conversion of a large number of WGBS experiments following our previously reported criteria (35) and selected two different sets of WGBS experiments to analyse the boundaries. WGBS set1 included SRR771524, which has 100 bp reads that render high coverage and exhibit high levels of telomeric cytosines conversion. WGBS set2 included four different experiments that have 101 or 151 bp reads and exhibit high levels of telomeric cytosines conversion (SRR3384734, SRR5494752, SRR5494753 and SRR5494754) (47,48). The sum of the four experiments also yielded high coverage. Thus, we analysed cytosine methylation at the telomere-subtelomere boundaries twice, once with WGBS set1 and a second time with WGBS set2. Bisulfite sequencing reads were aligned to the TAIR10-Tel genome using BSBolt v0.1.2 (49) using default settings. Duplicate reads were marked using SAMtools v1.9 (50). Following duplicate removal, methylation values were called for all observed cytosines with one or more reads covering the cytosine with a base call quality above 25 using BSBolt v0.1.2 (49).

### DNA methylation analyses

Statistical analyses and graphical representations were performed using the data provided in Supplementary Tables and the SPSS v25 statistical program. Statistical levels of significance of pair-wise comparisons were determined using two-sided *U* of Mann–Whitney or Student *T* tests, depending on whether the distributions were normal or not according to the Shapiro–Wilk test. *P* values are indicated in the legend of the figures or in the text and also in Supplementary Table S2 together with additional statistical parameters. Comparisons rendering *P*-values <0.05 were considered significant.

To analyse interrelationships between methyltransferases and the C context, we performed a principal components analysis. For that purpose, we divided the subtelomeric sequences undergoing DNA methylation into 40 bp tiles. For every tile, we determined the levels of CGm, CHGm and CHHm in the WT and in the methyltransferase mutants as well as the densities of CG, CHG, CHH, (CAG + CTG) and CCG sites. Then, the principal components analysis with varimax rotation was performed using SPSS. Three components that correlate with CGm, CHGm and/or CHHm in the WT and with eigenvalues higher than 1 were selected. These components explained 53% of the variance (21% PC1, 19% PC2 and 13% PC3).

To study the overlapping influence of methyltransferases on subtelomeric DNA methylation, we analysed differentially methylated tiles (DMTs). To that end, we focused on 40 bp tiles that had at least 20% of any type of methylation in the WT. We referred to those tiles as CGm, CHGm and/or CHHm labelled tiles. Tiles with WT levels of CGm, CHGm or CHHm <20% were considered to have low levels of the corresponding types of methylation, which were not further analysed. In this way, we analysed robust methylation activities and minimized background noise. We considered DMTs those that had lower levels of methylation in the mutants than in the WT (at least 20% for CGm, 15% for CHGm and 10% for CHHm).

To estimate the influence of the different methyltransferases on non-CGm, we calculated their contribution to CHGm and CHHm as the percentages of WT methylation that disappear in the corresponding mutants. We calculated the contributions of methyltransferases to individual telomeric regions, to all telomeric regions and to the Ta3 retrotransposon.

Clustering analysis was performed by calculating the euclidean distance between all samples using all methylation sites (CG, CHG, CHH). Hierarchical clustering was then performed using the nearest point algorithm as implemented in scipy v1.4.1 (51).

## RESULTS

### DNA sequence organization of telomeric regions in *Arabidopsis thaliana*

*Arabidopsis thaliana* has five chromosomes and, therefore, ten chromosome ends. Two of these ends, the left telomeric regions of chromosomes 2 and 4 (2L and 4L, respectively), contain long arrays of ribosomal genes and have not been fully sequenced. The remaining ends contain canonical telomeres, which consist of tandem arrays of the plant type telomeric repeat (CCCTAAA) that extend 2.5–5 kbp. However, the currently used Arabidopsis reference genome sequence (TAIR10) does not include these terminal repeats for most chromosome ends (36).

We used previously released genome-wide DNA sequence data to complete the sequences of several TAIR10 chromosome ends (37–43). Using these data, we connected 3L, 5L, 1R and 5R with telomeres. In addition, we incorporated to TAIR10 the sequences of 1L and 3R, which had been previously published (44,45). This modified version of the genome, which we denote as TAIR10-Tel, allows the study of cytosine methylation at Arabidopsis telomeric regions using WGBS experiments (Supplementary Figures S1 and S2).

Telomeres in TAIR10-Tel are represented by an array of 10 perfect telomeric CCCTAAA repeats (considering forward sequences for the left telomeric regions and reverse complementary sequences for the right telomeric regions). These repeats expand 70 bp and are followed by degenerate telomeric repeats interspersed with perfect telomeric repeats, which we denote as subtelomeric ITSs (Supplementary Figure S2). The perfect and degenerate telomeric repeats of ITSs have the same head to tail orientation than the perfect repeats present in their corresponding telomeres. Thus, the DNA strand that holds the C-rich repeats of telomeres is the same strand that accommodates the C-rich strand of ITSs. In most cases, subtelomeric ITSs abut the perfect telomeric repeats of telomeres and, therefore, localize at the telomere-subtelomere boundaries. However, a mitochondrial DNA insertion separates the telomere from the subtelomeric ITS in 1L (44). Interestingly, this insertion shares conserved motifs with DNA sequences embedded within the 5R subtelomeric ITS (2). Thus, the two sequences could be evolutionarily related.

Specific types of degenerate telomeric repeats are very abundant within subtelomeric ITSs (Supplementary Table S3 and Supplementary Figure S2). Although the Arabidopsis perfect telomeric repeat sequences (5′-CCCTAAA-3′ and 5′-TTTAGGG-3′) can generate up to 42 different variants containing one mismatch, only 15 of these variants are found within subtelomeric ITSs. Interestingly, about half of these degenerate repeats are present in more than one subtelomeric ITS, with some of them localized in up to four different ITSs. In addition, most subtelomeric ITSs contain several types of degenerate telomeric repeats. Hence, specific types of degenerate telomeric repeats are enriched at subtelomeric ITSs.

### Subtelomeric DNA methylation is heterochromatic and extends up to about 1–2 kbp

**Figure 1.**
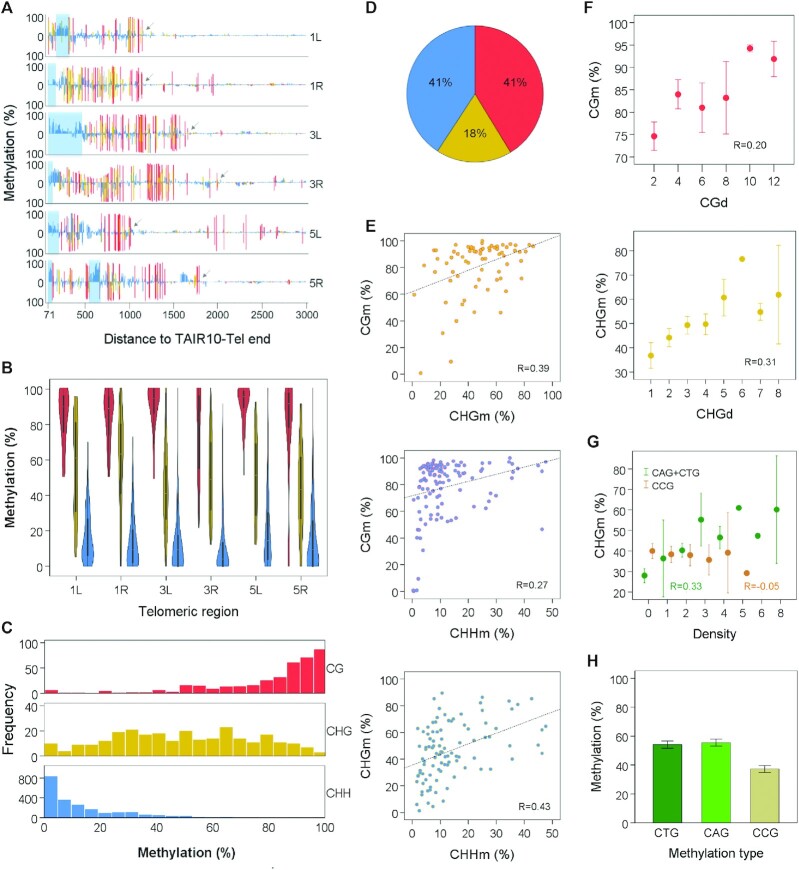
Subtelomeres exhibit heterochromatic DNA methylation. (**A**) Subtelomeric DNA methylation profiles. Bar plots represent the percentages of methylation at individual cytosines along 1L, 1R, 3L, 3R, 5L and 5R, from positions 71 to 3000 relative to TAIR10-Tel ends. Hereinafter, coordinates correspond to the sequences displayed in Supplementary Figure S2. CGm, CHGm and CHHm are indicated in red, yellow and blue, respectively. Whereas methylation of the forward strand is represented above the cero line, reverse strand methylation is represented below. Light-blue rectangles demarcate the positions of subtelomeric ITSs and arrows point to the last CG sites in the right borders of subtelomeric DNA methylation, according to our arbitrary criteria. Their coordinates have been used to delimitate the extent of subtelomeric DNA methylation in further analyses. The high levels of CGm detected at the right sides of 1R and 5L are not heterochromatic because they are not accompanied by CHGm and CHHm. These high levels of CGm might associate with the expression of At5g01010 in 5L and of a previously unidentified gene in 1R. (**B**) Violin plots showing that the levels of CGm are higher than the levels of CHGm, which, in turn, are higher than the CHHm levels in 1L, 1R, 3L, 3R, 5L and 5R (*P* < 0.001 in all cases). (**C**) Histograms representing the frequencies of subtelomeric CG, CHG and CHH sites with different methylation percentages. (**D**) Pie chart showing the percentages of subtelomeric methylcytosines in the different sequence contexts. (**E**) Scatter plots showing correlations between the different types of subtelomeric cytosine methylation. Pearson correlation coefficients are shown (*P* < 0.003 in all cases). (**F**) Scatter plots showing correlations of CGm with CGd and CHGm with CHGd. CGd and CHGd refer to the number of CG and CHG sites within 40 bp tiles. Pearson correlation coefficients are shown (*P* = 0.035 and 0.002, respectively). Mean values ± SEM are represented in this panel and in panels G and H. (**G**) Scatter plots showing correlation of CHGm with (CAG + CTG)d (*R* = 0.33, *P* = 0.001) but not with CCGd (*R* = −0.05, *P* = 0.626). (**H**) Bar plot showing that the subtelomeric CHGm levels are lower in the CCG context than in the CAG or CTG contexts (*P* < 0.001). All panels in this figure were performed by analysing cytosines between positions 71 and the right limits of subtelomeric DNA methylation (see arrows in Figure 1A). Panels A–D and H were performed by analysing untiled cytosines and correspond with Supplementary Table S4. The levels of methylation obtained for every subtelomeric cytosine from experiments SRR534177 + SRR534193 and SRR771524 were averaged and used for representations. Panels E–G were performed by analysing tiled cytosines and correspond with Supplementary Table S5. The levels of methylation obtained for every subtelomeric tile from experiments SRR534177 + SRR534193 and SRR771524 were averaged and used for representations.

We analysed DNA methylation at *Arabidopsis thaliana* subtelomeres. To accomplish this, we first selected high quality WGBS experiments and mapped them to TAIR10-Tel. Then, we determined the levels of the different types of cytosine methylation. We generated high-resolution DNA methylation profiles of subtelomeres, from position 71 to 3000 (Figure 1A). These profiles revealed that subtelomeric DNA methylation extends from the telomere-subtelomere boundaries up to about 1–2 kbp into subtelomeres.

Arabidopsis heterochromatin is characterized by the presence of CGm, CHGm and CHHm, with CGm being higher than CHGm, which, in turn, is higher than CHHm (52,53). We analysed these three types of cytosine methylation at subtelomeres and found average levels of CGm > CHGm > CHHm (Figure 1B). Whereas almost all subtelomeric cytosines in the CG context tend to be heavily methylated, most CHH cytosines have low levels of methylation. In turn, the frequencies of CHG cytosines with different levels of methylation do not show a skewed distribution (Figure 1C). However, since CHH sites are more abundant than CHG or CG sites, the percentages of subtelomeric methylcytosines that are in the CHH and CG contexts are similar and higher than the percentage of methylcytosines in the CHG context (Figure 1D). Therefore, subtelomeres have the characteristic DNA methylation pattern of Arabidopsis heterochromatin.

We performed correlation analyses to search for interdependence between the different types of subtelomeric cytosine methylation and the density of the different methylation sites. To this end, we first divided the subtelomeric sequences into 40 bp tiles. Then, we determined the levels of CGm, CHGm and CHHm as well as the densities of CG, CHG and CHH sites. Our analyses revealed positive correlations between the three types of cytosines methylation (Figure 1E). In addition, we noticed that CGm and CHGm had a positive correlation with the densities of CG and CHG (CGd and CHGd), respectively (Figure 1F). More specifically, we found that CHGm correlates with (CAG + CTG)d but not with CCGd (Figure 1G). In agreement with this result, we observed that subtelomeric cytosines in the CAG and CTG contexts are methylated more efficiently than subtelomeric cytosines in the CCG context (Figure 1H). All these results are in agreement with previously reported WGBS studies and reveal that subtelomeres are a good model system for the study of heterochromatic DNA methylation in Arabidopsis (52,53).

### Subtelomeric DNA methylation drops at the telomere-subtelomere boundaries

**Figure 2.**
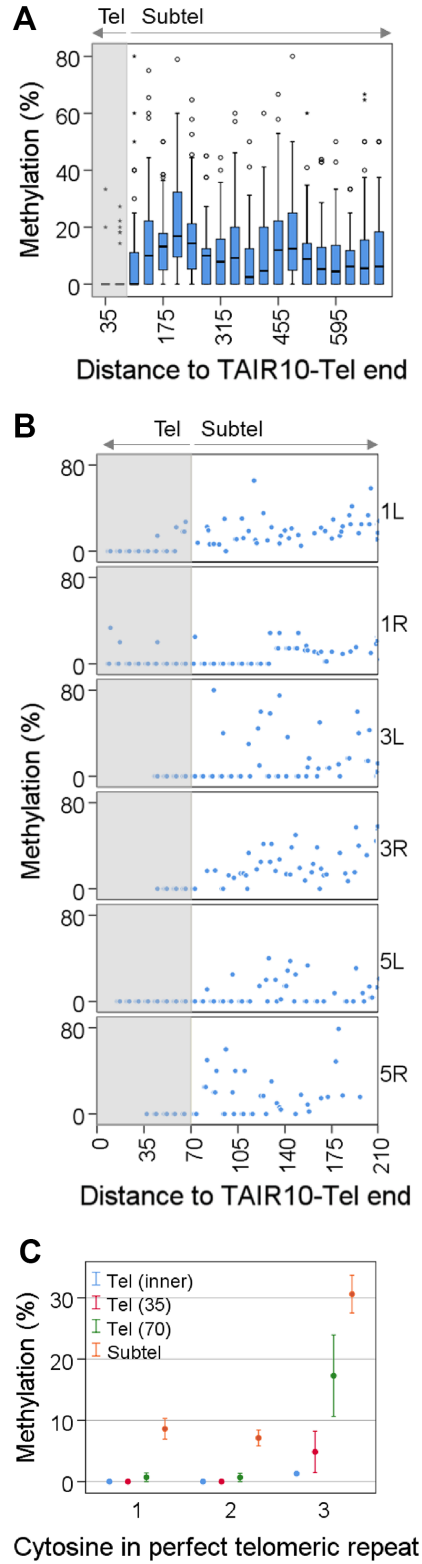
Cytosine methylation drops at the telomeric sides of the telomere-subtelomere boundaries. (**A**) Box plots showing the percentages of CHHm along the first 700 bp of telomeric regions after dividing them into 35 bp tiles. The distance of tiles to TAIR10-Tel ends is indicated. The first two tiles (35 and 70) are telomeric (Tel) and have significant lower levels of methylation than the rest of the tiles, which are subtelomeric (Subtel) (*P* < 0.001). The grey shadow demarcates telomeric tiles. (**B**) Scatter plots showing the methylation levels of individual CHH cytosines along the first 210 bp of 1L, 1R, 3L, 3R, 5L and 5R. The distance of cytosines to TAIR10-Tel ends is indicated. The grey shadow demarcates telomeric cytosines. (**C**) Scatter plots showing the methylation levels of the first, second and third cytosines of the perfect telomeric repeats present at the inner part of telomeres, Tel (inner), at the telomeric sides of the boundaries, Tel (35) and Tel (70), and at the subtelomeric tiles shown in panel A (Subtel). Methylation values for the inner part of telomeres have been obtained from Supplementary Figure S4, assuming that most telomeric reads methylation occurs at the third cytosine of the perfect telomeric repeats. This figure has been performed using experiment SRR771524 (WGBS set 1) and corresponds with Supplementary Table S6.

Since Arabidopsis telomeres have been reported to be unmethylated and subtelomeres undergo DNA methylation, we decided to analyse cytosine methylation at telomere-subtelomere boundaries. We analysed the levels of methylation in the 70 bp that represent telomeres in TAIR10-Tel and in the adjacent subtelomeric sequences. Considering that the perfect telomeric repeats of Arabidopsis telomeres are of the CCCTAAA type and, therefore, only contain CHH cytosines, we focused on CHHm. We found that the levels of CHHm drop at the telomeric sides of the telomere-subtelomere boundaries (Figure 2A,B). However, certain levels of CHHm methylation that vary among experiments can be observed within the 10 perfect telomeric repeats that represent telomeres in the boundaries (Figure 2A,B and Supplementary Figure S3A,B). Therefore, the drop of cytosine methylation at the telomere-subtelomere boundaries is not abrupt but progressive. This decrease of CHHm can also be observed when only perfect telomeric repeats are analysed. Previous reports have shown that the three cytosines of the perfect telomeric repeat units that localise at ITSs are methylated, with the third one methylated more efficiently than the first and the second. However, the three cytosines of the perfect telomeric repeats units present at the inner part of telomeres remain essentially unmethylated (35,53). Thus, a decrease in the levels of perfect telomeric repeats methylation would be expected at the telomere-subtelomere boundaries. We detected higher levels of methylation in the third cytosine of the telomeric repeat units than in the first and second cytosines (Figure 2C and Supplementary Figure S3C). This could be observed at subtelomeres and also at the telomeric side of the telomere-subtelomere boundaries. However, the methylation levels of the three cytosines decrease at the telomeric side of the boundaries and disappear at the inner part of telomeres. Thus, boundaries are transition regions characterized by a shift in DNA methylation levels.

To determine the levels of DNA methylation at the perfect telomeric repeats present in the inner part of telomeres we analysed the reads that represent telomeres in WGBS studies, which follow the (YYYTAAA)n pattern, as previously reported (35). We found that, in all the experiments analysed, the inner telomeric reads were essentially unmethylated showing from 0.5% to 1.3% methylated cytosines (Supplementary Figure S4), which are in the range of the CHHm error rates determined for the corresponding experiments (Supplementary Table S1). This result and the insensitivity of the Arabidopsis telomeric DNA to methylation-dependent restriction enzymes previously reported (35) are in contradiction with previous proposals stating that Arabidopsis telomeres undergo DNA methylation (10,54). However, the low levels of DNA methylation detected at the telomeric side of the boundaries could contribute to reconcile both views to certain extent.

### DNA and Histone H3K9 methyltransferases cooperate to maintain subtelomeric DNA methylation

**Figure 3.**
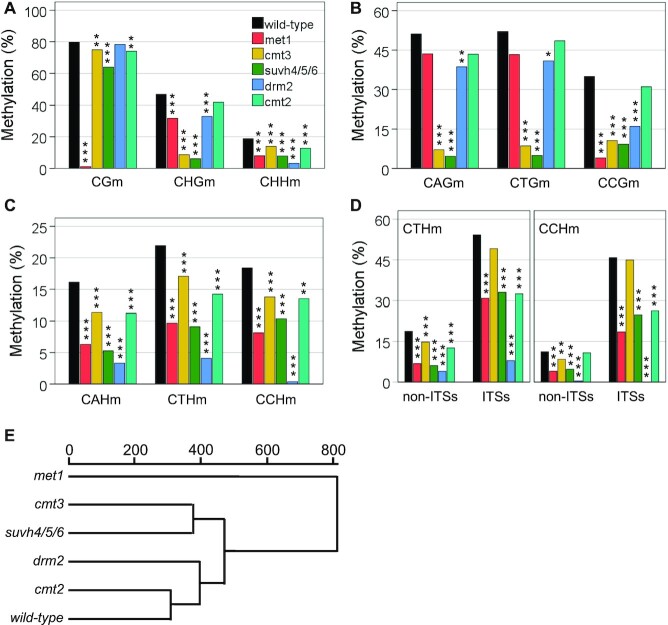
DNA and histone methyltransferases cooperate to maintain subtelomeric DNA methylation. (**A**) CGm, CHGm and CHHm levels at subtelomeres. Here and in the following panels bar plots represent the mean levels of methylation in the WT strain and in DNA methylation mutants, which are labelled with different colours. Significant differences of DNA methylation levels between the WT and the DNA methylation mutants are indicated with asterisks (*P < 0.05, **P < 0.01, ***P < 0.001). (**B**) Influence of the cytosine context on subtelomeric CHGm. Although it is not indicated, the levels of CAGm and CTGm are significantly higher than those of CCGm in *met1*, *drm2* and *cmt2* (*P* < 0.05 in all cases) but not in *cmt3* or *suvh4/5/6* (*P* > 0.05 in all cases). (**C**) Influence of the cytosine context on subtelomeric CHHm methylation. (**D**) Subtelomeric CTHm and CCHm levels within and outside ITSs. Although it is not indicated, all strains have significantly enhanced levels of CTHm and CCHm within ITSs (*P* < 0.001 in all cases) except *drm2* (*P* = 0.213 and *P* = 0.502, respectively). (**E**) Hierarchical clustering of methyltransferase mutants together with the WT according to their euclidean distance between the telomere methylation profiles (CGm, CHGm and CHHm). Panels A–D correspond with Supplementary Table S7 and panel E corresponds with Supplementary Table S8.

To further understand the nature of subtelomeric DNA methylation, we decided to analyse several Arabidopsis DNA methylation mutants. These mutants encode inactive versions of DNA or histone methyltransferases. We analysed *met1*, *cmt3*, *suvh4/5/6*, *drm2* and *cmt2* (Figure 3A). As expected, we found that subtelomeric CGm is essentially abolished in *met1* and CHGm is greatly reduced in *cmt3* and *suvh4/5/6*. These results are in accord with the notion that MET1 and CMT3 are the major subtelomeric CG and CHG methyltransferases, respectively, and that SUVH4/5/6 strongly influences CHGm. We detected greatly reduced levels of CHHm in the *drm2* mutant, which suggests that subtelomeric CHHm is mainly achieved by DRM2. In addition, we found that CHGm and CHHm are reduced, to different extents, in all the mutants and that CGm is moderately reduced in *suvh4/5/6* and slightly decreased in the other mutants. Thus, DNA and histone H3K9 methyltransferases cooperate to maintain the three types of subtelomeric DNA methylation.

We have shown that subtelomeric CHGm correlates with CAGd and CTGd and is more effective in the CAG and CTG contexts than in the CCG context (Figure 1G and H). To further understand this bias of CHGm towards CAGm and CTGm, we decided to analyse the different types of CHGm in the mutants (Figure 3B). We found that the levels of CAGm and CTGm are higher than those of CCGm in *met1*, *drm2* and *cmt2* but not in *cmt3* or *suvh4/5/6*. Thus, the bias of CHGm towards CAGm and CTGm is related to SUVH4/5/6 and CMT3 but not to the other methyltransferases. We detected slight reductions of CAGm and CTGm in *met1* and *drm2*. However, the levels of CCGm are significantly decreased in these mutants. Specially in *met1* who has lower CCGm levels than *cmt3* and *suvh4/5/6*. This result is not surprising because previous studies have shown that MET1 is required for CCGm and only have a slight influence on CAGm and CTGm at genome-wide level (55,56). Thus, both, MET1 and DRM2, influence subtelomeric CHGm mainly in the CCG context. In turn, the *cmt2* mutant only exhibits slight reductions of CAGm, CTGm and CCGm. Hence, CMT2 has a low influence on subtelomeric CHGm in the three sequence contexts.

To gain insight into the influence of the cytosine context on CHHm we studied the levels of subtelomeric CAHm, CTHm and CCHm (Figure 3C). We found that the levels of the three types of methylation were reduced in all the DNA methylation mutants, being lower in *drm2* where the CCHm levels were almost undetectable. Therefore, DRM2 performs most of the subtelomeric CHHm and is essential for subtelomeric CCHm. In turn, the influence of CMT2 on the three types of CHHm is lower that the influence of MET1 and SUVH4/5/6.

Considering the potential relevance of subtelomeric ITSs, we decided to analyse CHHm methylation at subtelomeric regions within and outside ITSs (Figure 3D). Subtelomeric ITSs start with the first degenerate repeats located after the 10 perfect tandem telomeric repeats that represent telomeres in TAIR10-Tel and contain degenerate telomeric repeats that are usually interspersed with perfect telomeric repeats, which in Arabidopsis are of the CCCTAAA type. Since most of the perfect and degenerate telomeric repeats of subtelomeric ITSs only contain cytosines in the CCH and CTH contexts, we decided to focus our analyses on these kinds of sites. Interestingly, we detected higher levels of CTHm within ITSs than in the rest of the subtelomeric cytosines that undergo DNA methylation. These enhanced levels of CTHm could be observed in the WT and in all methylation mutants, although only a small non-significant increase could be detected in *drm2*. Similarly, we observed enhanced levels of CCHm within ITSs in the WT and in all methylation mutants, with the exception of *drm2*. Hence, DRM2 is responsible for the highly enhanced levels of CHHm found within ITSs in the WT. We found that MET1, SUVH4/5/6 and CMT2 influence CTHm within and outside ITSs. However, whereas MET1 and SUVH4/5/6 also influence CCHm within and outside ITSs, CMT2 only influences CCHm within ITSs. Thus, DRM2 is essential for CCHm within and outside ITSs and performs all CCHm outside ITSs.

In summary, the analysis of DNA methylation mutants reveals extensive cooperation of multiple methyltransferases to maintain the different types of subtelomeric DNA methylation. Nevertheless, each methyltransferase is mainly associated with a specific methylation type. This is confirmed by clustering of DNA methylation mutants based on similarities between CGm, CHGm and CHHm profiles, which reveals three different groups (Figure 3E). These groups involve (1) *met1*, (2) *cmt3* and *suvh4/5/6* and (3) *drm2*, *cmt2* and WT. Since CGm, CHGm and CHHm are mainly mediated by MET1, CMT3 together with SUVH4/5/6 and DRM2 or CMT2, respectively, this clustering analysis groups the mutants according to their major DNA methylation activities.

### Primary DNA sequences condition the influence of methyltransferases on subtelomeric DNA methylation

**Figure 4.**
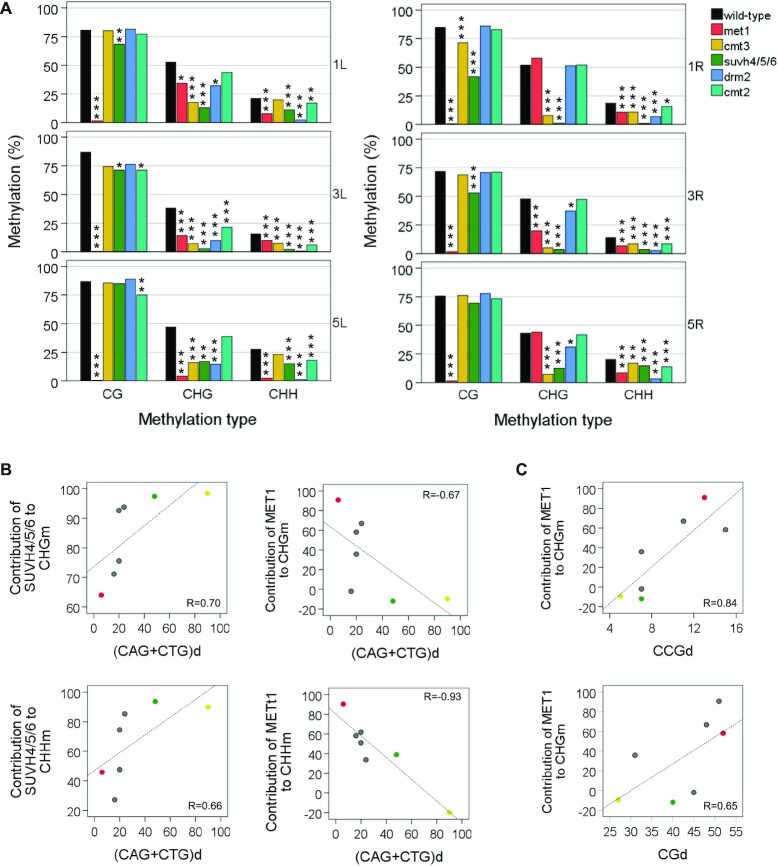
Primary DNA sequences condition the influence of methyltransferases on subtelomeric DNA methylation. (**A**) The influence of methyltransferases on DNA methylation varies among subtelomeres. Bar plots represent mean CGm, CHGm and CHHm levels at different subtelomeres in the WT strain and in methyltransferase mutants. Significant differences of DNA methylation levels between the WT and the DNA methylation mutants are indicated with asterisks (*P < 0.05, **P < 0.01, ***P < 0.001). This panel corresponds with Supplementary Table S7. (**B** and **C**) The contributions of SUVH4/5/6 and MET1 to non-CGm correlate with the context of cytosines. Scatter plots represent the contributions of methyltrasferases versus the density of cytosines at 1L, 1R (green), 3L, 3R, 5L (red), 5R and at the Ta3 retrotransposon (yellow). The contributions of methyltransferases to CHGm and CHHm were determined as the percentages of wild-type methylation that disappear in the corresponding mutants. Density values indicate the number of (CAG + CTG), CCG or CG sites per kbp. Pearson correlation coefficients are shown. Associated *P*-values are below 0.12 in all cases. Correlation coefficients obtained only for subtelomeric regions, without including Ta3 in the analysis, are between 0.52 and 0.80 and between −069 and −075.

To further understand the influence of methyltransferases on subtelomeric DNA methylation, we examined subtelomeres independently. To that end, we first determined the levels of CGm, CHGm and CHHm for each subtelomere in the WT and in the mutants (Figure 4A). Then, we tiled subtelomeric DNA sequences undergoing DNA methylation into 40 bp tiles and examined the resulting mutant profiles together with those of the WT (Supplementary Figure S5). As expected, we found that the lowest levels of CGm, CHGm and CHHm are present in *met1*, *cmt3* or *suvh4/5/6* and *drm2*, respectively, at most subtelomeres. However, this is not always the case. Indeed, the influence of methyltransferases on the different types of methylation varies among subtelomeres. This differential behavior of methyltransferases can be clearly observed when comparing the levels of CHGm and CHHm in *met1* and *suvh4/5/6*. Whereas at 5L the levels of CHGm and CHHm are lower in *met1* than in *suvh4/5/6*, at 1R both levels of methylation are lower in *suvh4/5/6* than in *met1*. Thus, MET1 has a stronger influence on non-CGm than SUVH4/5/6 at 5L and SUVH4/5/6 has a stronger influence than MET1 at 1R (Figure 4A and Supplementary Figure S5). These results reveal that the influence of methyltransferases vary among subtelomeres and largely rely on the primary subtelomeric DNA sequences. This phenomenon has also been reported in humans where the lack of the DNA methyltransferase DNMT3B influences DNA methylation at certain subtelomeres more than at others (57).

In order to quantify the influence of MET1 and SUVH4/5/6 on subtelomeric non-CGm, we decided to determine their contributions to CHGm and CHHm at the different subtelomeres as the percentages of wild-type methylation that disappear in the corresponding mutants. In addition, we analysed whether these contributions are influenced by the context of cytosines. Interestingly, we found that whereas the contribution of SUVH4/5/6 to non-CGm tends to increases with (CAG + CTG)d, the contribution of MET1 tends to decrease with it (Figure 4B). In addition, the contribution of MET1 to CHGm tends to increase with CCGd and CGd (Figure 4C). These results suggest that the density of the different types of cytosines at specific subtelomeres conditions the contributions of MET1 and SUVH4/5/6 to non-CGm.

### Overlapping influence of methyltransferases on subtelomeric DNA methylation

**Figure 5.**
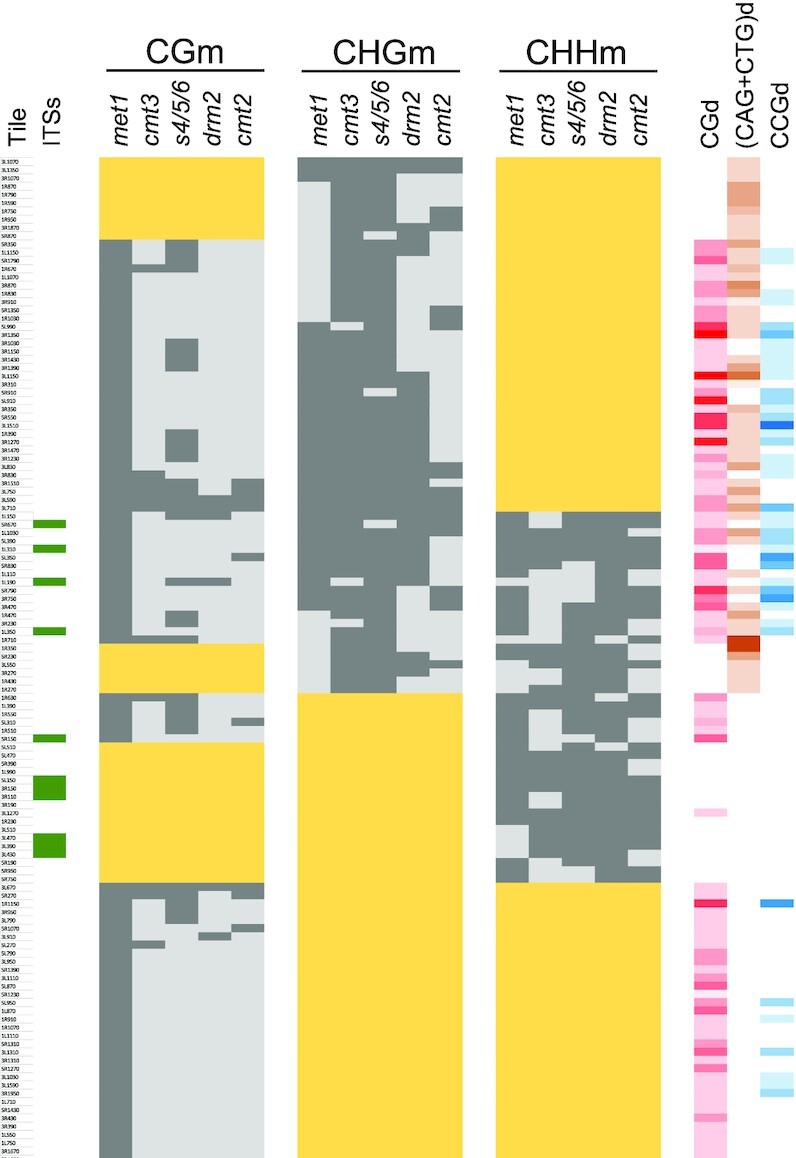
The influence of methyltransferases on DNA methylation overlap along subtelomeres. A classification of subtelomeric tiles according to their levels of methylation in methyltransferase mutants is shown. Wide yellow rectangles label tiles with levels of CGm, CHGm and/or CHHm below 20% in the WT strain. Whereas dark grey rectangles correspond with DMTs, light grey rectangles indicate the absence of differential methylation. The presence of ITSs in specific tiles is indicated by green rectangles in the left. The densities of CG, (CAG + CTG) and CCG sites are indicated in the right with different colours. The intensity of the colours (red, orange and blue, respectively) is proportional to the number of the sites, with white representing cero sites. This figure corresponds with Supplementary Table S8.

To further dissect the role of methyltransferases at subtelomeres, we decided to perform an analysis of differentially methylated tiles (DMTs). This analysis allowed us to study how the influence of different methyltransferases overlaps in subtelomeres. We divided the portions of subtelomeric regions that undergo DNA methylation into 40 bp tiles and focused on those tiles that had at least 20% of any type of methylation in the WT. We referred to those tiles as CGm, CHGm and/or CHHm labelled tiles. In addition, we defined as DMTs those that had lower levels of methylation in the mutants than in the WT (at least 20% for CGm, 15% for CHGm and 10% for CHHm). We considered that a specific methyltransferase influences one type of methylation in a defined tile when this tile is differentially methylated for this type of methylation in the corresponding mutant. We found that MET1 influences CGm in all CGm-labelled tiles and that SUVH4/5/6 also influences CGm in 35% of these tiles (Figure 5). In addition, CMT3, DRM2 and CMT2 affect CGm in a small proportion of the tiles (7–12%), most of which are also influenced by SUVH4/5/6. Thus, the influence of SUVH4/5/6 on CGm is higher and more widespread than the influence of CMT3, DRM2 or CMT2 (Figure 3A and Figure 5).

CMT3 together with SUVH4/5/6 influence CHGm in 95% of the CHGm-labelled tiles (Figure 5). In turn, MET1, DRM2 and CMT2 affect CHGm in 59%, 54% and 31% of these tiles, respectively. Thus, although CMT3 is the major subtelomeric CHG methyltransferase and affects most CHGm-labelled tiles, it influences CHGm in concert with MET1, DRM2 and, to a lower extent, with CMT2. We analysed whether the influence of MET1, DRM2 and CMT2 on CHGm is biased by the context of cytosines. We found that MET1 tends to influence CHGm in CHGm-labelled tiles with high CGd and CCGd and low (CAG + CTG)d (*P* < 0.003 in all cases). By contrast, neither DRM2 nor CMT2 tend to influence CHGm-labelled tiles with high or low CGd, CCGd or (CAG + CTG)d (*P* > 0.116 in all cases). However, when DRM2 influences CHGm together with MET1 it targets tiles with higher CGd and CCGd than when it influences CHGm independently (*P* ≤ 0.02). Similarly, when CMT3 influences CHGm together with MET1 it also targets tiles with higher CGd and CCGd (*P* < 0.002). Thus, the cooperation of MET1 with DRM2 and CMT3 to maintain CHGm along subtelomeres is mainly achieved in tiles enriched in CG and CCG sites.

DRM2 influences subtelomeric CHHm in 93% of the CHHm-labelled tiles, including all the tiles that contain subtelomeric ITSs (Figure 5). In addition, CMT2 influences CHHm in 56% of the CHHm-labelled tiles, including those that are not affected by DRM2. Thus, DRM2 and CMT2 account for all CHHm along subtelomeres, where they can cooperate to maintain it. We found that 79% of the tiles where DRM2 influences CHHm are affected by MET1 and 81% are affected by SUVH4/5/6, with 62% of them being simultaneously affected by the three methyltransferases. In addition, 81% of the tiles where CMT2 influences CHHm are affected by MET1 and also 81% are affected by SUVH4/5/6, with 67% of them being simultaneously affected by the three of them. Therefore, the concerted action of DRM2 or CMT2 with MET1 and SUVH4/5/6 on CHHm is widespread along subtelomeres. However, whereas MET1 has certain preference to influence tiles with high CGd (*P* = 0.028), SUVH4/5/6 does not show a preference for tiles with high or low CGd, (CAG + CTG)d or CCGd (*P* > 0.05 in all cases).

In general, the analysis of DMTs supports the notion that the different methyltransferases cooperate to maintain DNA methylation within the same stretches of subtelomeres. However, this cooperation varies along subtelomeric tiles, which is likely influenced by their primary DNA sequences. This primary sequence influence is also revealed by a principal components analysis, which supports the existence of a MET1-dependent DRM2 activity on CHGm and that the activity of SUVH4/5/6, CMT3 and CMT2 on subtelomeric non-CGm increases with (CAG + CTG)d and decreases with CCGd (Supplementary Figure S6).

**Figure 6.**
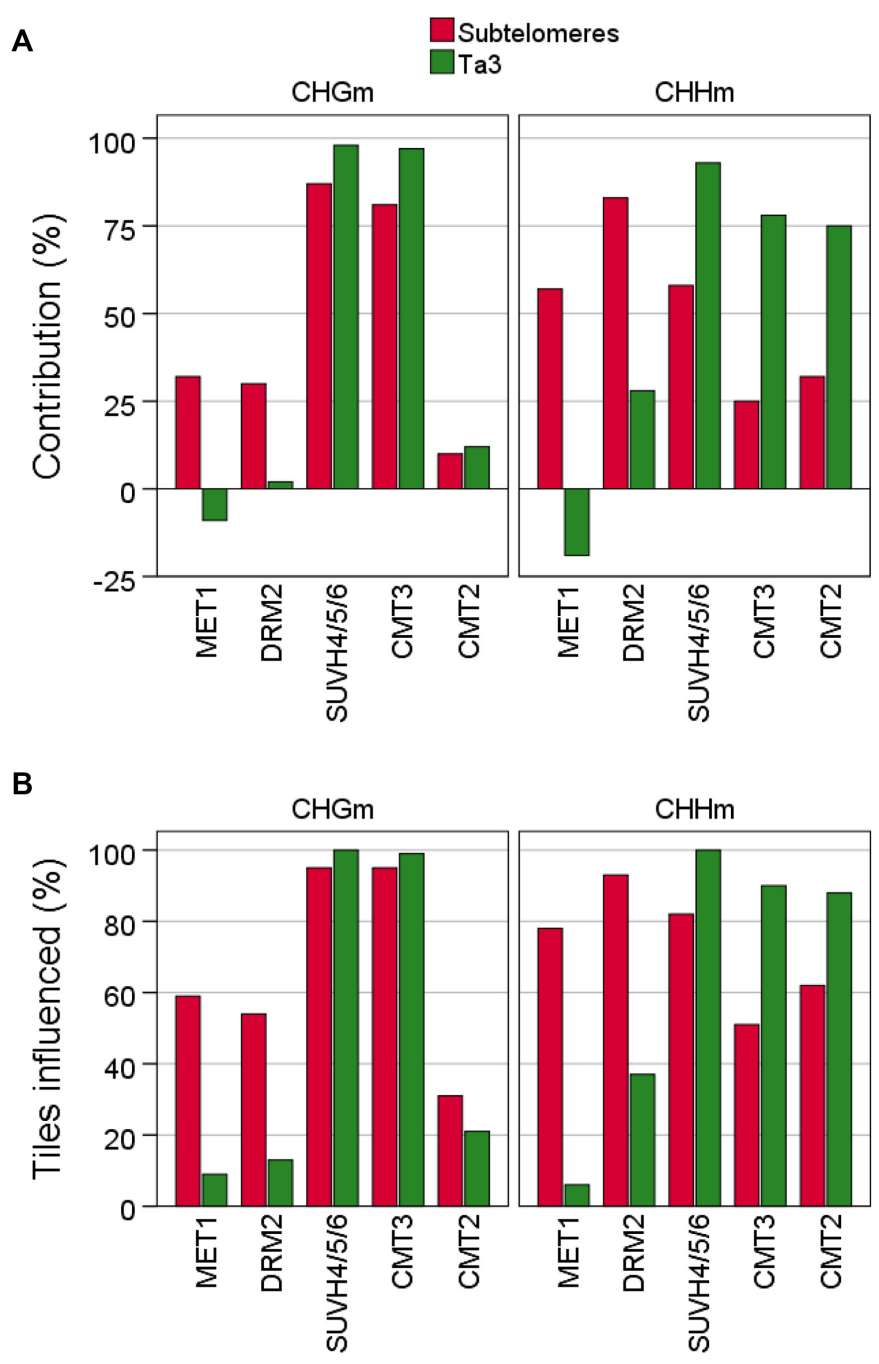
The influence of methyltransferases on non-CGm varies among subtelomeres and the Ta3 retrotransposon. (**A**) Contribution of meyltransferases to CHGm and CHGm. The contribution of each methyltransfease was calculated as the percentage of wild-type methylation that disappears in the corresponding mutants. The colour code corresponding to subtelomeres and Ta3 is indicated at the top. This panel corresponds with Supplementary Tables S7 and S9. (**B**) Percentages of CHGm- and CHHm-labelled tiles influenced by methyltransferases. These tiles are differentially methylated in the corresponding mutants. This panel corresponds with Supplementary Tables S8 and S10.

To obtain further insights into the influence of the primary sequences on the behavior of methyltransferases, we compared subtelomeres with the Ta3 retrotransposon, which has been studied as heterochromatic reference in multiple publications (Supplementary Figure S7) (32,58–60). We decided to study the Ta3 retrotransposon because it has higher (CAG + CTG)d and lower CGd/CCGd than all subtelomeres analysed here. We noticed that the contribution of MET1 and DRM2 to non-CGm is higher at subtelomeres than at Ta3 (Figure 6A, see also Figure 3 and Supplementary Figure S8). Accordingly, whereas MET1 and DRM2 influence non-CGm in a considerable percentage of subtelomeric tiles, their influence on non-CGm within Ta3 is less widespread (Figure 6B, see also Figure 5 and Supplementary Figure S9). In contrast, the contributions of SUVH4/5/6, CMT3 and CMT2 to non-CGm are lower at subtelomeres than at Ta3 (Figure 6A). Besides, their influence on non-CGm is generally less widespread at subtelomeres than at Ta3 (Figure 6B). Thus, MET1 and DRM2 play a more relevant role on non-CGm at subtelomeres than SUVH4/5/6, CMT3 and CMT2 and SUVH4/5/6, CMT3 and CMT2 play a more relevant role on non-CGm at Ta3 than MET1 and DRM2. Since the Ta3 retrotransposon has higher (CAG + CTG)d and lower CGd/CCGd than subtelomeres, these results are in agreement with the analysis of individual subtelomeres (Figure 4B and C) and further support that the behaviour of methyltransferases at specific subtelomeres is conditioned by the density of the different types of cytosines.

## DISCUSSION

### Telomere-subtelomere boundaries are transition regions characterized by a shift in genetic and epigenetic organization

To map DNA methylation at *Arabidopsis thaliana* telomeric regions, we have extended the DNA sequences of most chromosome ends present in the Arabidopsis TAIR10 reference genome. As a result, we have obtained a modified version of the genome that we denote as TAIR10-Tel. TAIR10-Tel reveals interesting features of the telomere-subtelomere DNA boundaries and has allowed us to study cytosine methylation at nucleotide resolution level in telomeric regions. In addition, TAIR10-Tel will allow future studies focusing on the epigenetic features of telomeric regions.

Telomeres in TAIR10-Tel are represented by 10 perfect telomeric repeats and are followed by subtelomeric sequences that contain ITSs at most chromosome ends. These subtelomeric ITSs abut or localize very near to Arabidopsis telomeres and contain specific types of degenerate telomeric repeats interspersed with perfect telomeric repeats. Interestingly, we detected higher levels of CHHm within ITSs than in the rest of subtelomeres that undergo DNA methylation. In turn, CHHm decreases at the telomeric sides of the telomere-subtelomere boundaries. This drop of cytosine methylation is not abrupt but progressive and is in agreement with previous studies showing that Arabidopsis telomeres are not methylated (35), which we have corroborated here. Thus, telomere-subtelomere boundaries are transition regions characterized by a shift in genetic and epigenetic organization.

Arabidopsis telomere-subtelomere boundaries could contribute to nucleate the formation of subtelomeric heterochromatin. Since ITSs localize at most boundaries, their perfect and degenerate telomeric repeats could play a role in this nucleation by recruiting Telomeric Repeats binding Factors (TRFs), which are very abundant in Arabidopsis. Whereas some TRFs are thought to bind the perfect telomeric repeats of telomeres and play telomeric functions, others are known to bind perfect and degenerated telomeric repeats outside telomeres and play additional functions (58–60). Therefore, some TRFs could contribute to recruit DRM2 to subtelomeric ITSs. DRM2 influences CHHm in all the subtelomeric tiles that contain ITSs, is responsible of the high levels of methylation found within ITSs and is essential for methylation in the CCH context, which is the context of the first and second cytosines of the perfect telomeric repeat units. Thus, DRM2 recruited to ITSs by TRFs could lead to significant levels of DNA methylation and spread methylation towards subtelomeres together with the other methyltransferases. The recruitment of DRM2 to ITSs could also involve POLIV and POLV (see below).

As in Arabidopsis, a bimodal chromatin organization of telomeric regions has been reported in humans. Whereas subtelomeres in humans are clearly heterochromatic, human telomeres exhibit low levels of heterochromatic marks (61–67). Interestingly, subtelomeric ITSs containing degenerate telomeric repeats can also be observed near to telomeres in human telomeric regions (68). Therefore, Arabidopsis and humans seem to share a similar organization at the telomere-subtelomere boundaries. It will be interesting to ascertain whether these boundaries contribute to seed subtelomeric heterochromatin formation and if, by doing so, influence telomere functions.

### A complex network of interactions governs subtelomeric DNA methylation

Subtelomeric cytosine methylation extends from telomeres up to about 2 kbp within subtelomeres. Different methyltransferases maintain subtelomeric DNA methylation. Whereas MET1 catalyses CGm, CMT3 and SUVH4/5/6 maintain most of the CHGm and DRM2 accomplishes most of the CHHm and part of the CHGm. In turn, CMT2 contributes to maintain CHHm and exerts a very minor role on CHGm maintenance. All these methyltransferases cooperate to maintain the three types of subtelomeric DNA methylation. However, the influence of MET1 on non-CGm is higher than the influence of the non-CGm machinery on CGm. Whereas CHGm in the *met1* mutant is strongly inhibited in the CCG context, CHHm is highly reduced in *met1* in the three contexts analysed (CAH, CTH and CCH). In turn, CGm is only slightly affected by mutations in *CMT3, DRM2* and *CMT2*, although it is moderately decreased in the *suvh4/5/6* mutant.

### Insights into subtelomeric CGm

The moderate influence of the non-CGm machinery on subtelomeric CGm might be related to the mechanism by which MET1 catalyses DNA methylation. Since MET1 methylates hemimethylated CG sites through DNA replication after being recruited by the VIM1-3 proteins, in principle, it should not be expected to depend on additional activities (8,12). However, subtelomeric CGm has a weak positive correlation with CGd, which has been previously shown at a genome-wide level (53,69). This correlation might reflect that certain regions with high CGd potentiate their own methylation by increasing the efficiency of MET1 recruitment and/or activity. Similarly, the moderate influence of the non-CG methylation machinery on subtelomeric CGm, which rely on SUVH4/5/6, might reflect that H3K9me2 can potentiate the recruitment and/or activity of MET1. SUVH5 could play a relevant role on this potentiation. Previous studies have shown that SUVH5 contributes more than SUVH4 or SUVH6 to the maintenance of H3K9me2 at CGm enriched regions and, in contrast to SUVH4 and SUVH6, moderately influence the genome-wide levels of CGm (69,70).

### Insights into subtelomeric CHGm

Genome-wide analyses of DNA methylation in Arabidopsis have shown that CHGm depends on CMT3 and SUVH4/5/6 and is preferentially found in the CAG and CTG contexts (52,53). In addition, these analyses have revealed that CCGm is strongly dependent on MET1 (55,56). Whereas the dependency of CHGm on CMT3 and SUVH4/5/6 has been related to the positive feedback loop that CMT3 establishes with SUVH4/5/6, the bias of CHGm towards CAGm and CTGm has been proposed to arise from the higher affinity that SUVH4 has for CAG and CTG sites, as compared to CCG sites. SUVH4 has been proposed to recruit CMT3 preferentially to regions with high CAGd and CTGd, thus potentiating CAGm and CTGm versus CCGm. In turn, SUVH5/6 would preferentially recruit CMT3 to regions with high CGd and CCGd and potentiate CCGm versus CAGm and CTGm. Since SUVH4 is the major Arabidopsis H3K9 methyltransferase, the result of this targeting balance would lead to the bias of CHGm towards CAGm and CTGm. In addition, the strong influence of MET1 on CCGm has been related to the recruitment of CMT3 by SUVH5/6 to methylated CG and CCG sites and/or to the inability of CMT3 to methylate the CCG motif if it is not previously methylated by MET1 (8,12,52,53,55,56,70).

Previous proposals can explain part of our results on subtelomeric CHGm, which are in full agreement with the results found in previous genome-wide studies and extend them. The high dependency of subtelomeric CHGm on CMT3 and SUVH4/5/6 should be related to the positive feedback loop that CMT3 establishes with SUVH4/5/6. In addition, the bias of subtelomeric CHGm toward CAGm and CTGm should be related to the recruitment of CMT3 by SUVH4. However, this scenario is far more complex because the activities of SUVH4/5/6 and CMT3 on CHGm coexist at subtelomeres with a MET1-dependent DRM2 activity on CHGm. MET1 could influence the catalytic action of DRM2 on CHGm and its recruitment. On the one hand, the catalytic action of DRM2 on CCG sites might require their previous methylation by MET1. Our DMTs and principal components analyses support this notion. On the other hand, MET1 could potentiate the recruitment of DRM2 at certain subtelomeres enriched in CGm through the action of POLV (see below). In this context, the strong reduction of subtelomeric CCGm observed in the *met1* mutant could be related to the reduced recruitment of DRM2 and CMT3 by POLV and SUVH5/6, respectively, and to the inefficient methylation of CCG sites by DRM2 and CMT3.

### Insights into subtelomeric CHHm

Subtelomeric CHHm is mainly mediated by DRM2 and is highly influenced by SUVH4/5/6 and MET1. By contrast, the influence of CMT2 on subtelomeric CHHm is modest and largely associates with SUVH4/5/6. Previously reported results could help to explain the influence of MET1 and SUVH4/5/6 on subtelomeric CHHm. MET1 could influence subtelomeric CHHm through SUVH2, which can target POLV to heterochromatin. Since SUVH2 binds preferentially to methylated CG sites and POLV recruitment and transcription is enhanced at regions enriched in CGm and requires MET1, SUVH2 bound to methylated CG sites could recruit POLV and, as a consequence, drive DRM2 activity on CHGm and CHHm at subtelomeres enriched in CG sites (29,30,71). In turn, SUVH4/5/6 might potentiate subtelomeric CHHm through the establishment of H3K9me2 and the consequent recruitment of CMT2 and DRM2. Whereas CMT2 would bind directly to H3K9me2, DRM2 would be targeted indirectly through SHH1. SHH1 bound to methylated H3K9me2 could recruit POLIV and, as a consequence, drive DRM2 activity on subtelomeric CHGm and CHHm. (8,12,21–23). Methylated CHH sites could also potentiate their own methylation through SUVH9, which is known to bind preferentially CHH methylated sites and can recruit POLV. Thus, POLV recruited by SUVH9 could target DRM2 activity on CHGm and CHHm to regions enriched in methylated CHH sites (29,30).

We have found that the concerted action of methyltransferases is widespread along subtelomeres. Considering that we have analysed short 40 bp tiles, we speculate that the concerted action of methyltransferases might involve physical interactions among them that could be required for their activities and, therefore, partially explain their cooperation. Such interactions might be particularly relevant at specific subtelomeric tiles like those that localize at the telomeric side of 3L. At these tiles, all methyltransferases are required for CGm and all methyltransferases but MET1 are required for non-CG methylation (see Supplementary Figure S5).

### A model for DNA methylation at telomeric regions

**Figure 7.**
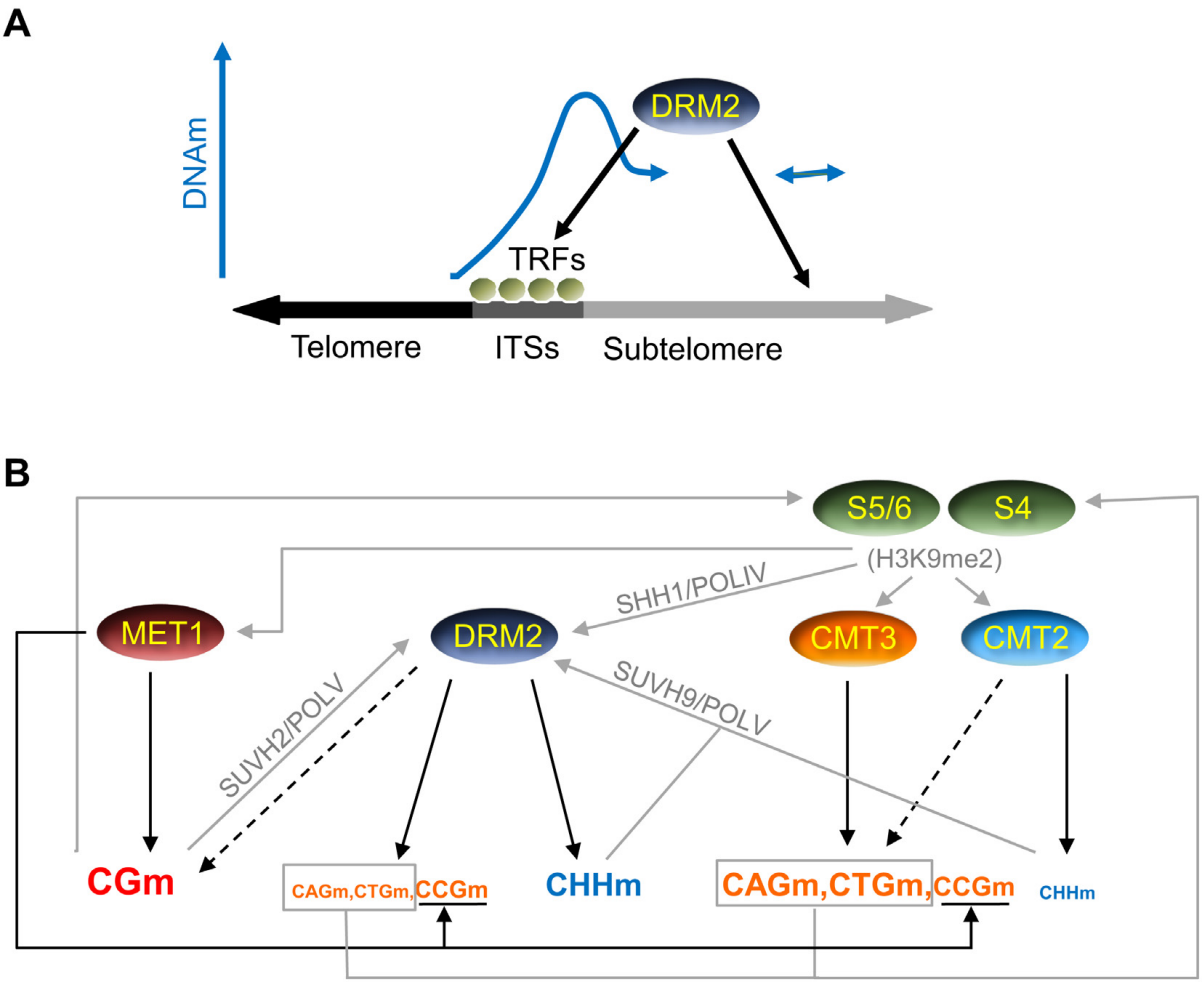
Model for DNA methylation at telomeric regions. (**A**) DRM2 recruitment to subtelomeres and initiation of DNA methylation spreading. ITSs bound by specific Telomeric Repeats binding Factors (TRFs) and additional subtelomeric loci recruit DRM2, which initiates the spreading of subtelomeric DNA methylation. Although some TRFs should also bind to telomeres and play pivotal functions, it is not reflected in the model for simplicity. (**B**) Recruitment pathways and methylation activities reinforcing the spreading of subtelomeric DNA methylation and its maintenance. Whereas DNA methylation activities are indicated by black arrows, putative recruitment pathways are indicated by grey arrows. The size of the letters used to represent the different types of methylation increases with the activities of the DNA methyltransferases that achieve them, as inferred from Figure 3. Black discontinuous arrows represent slight activities of CMT2 on CHGm and of DRM2 on CGm. See text for further explanations.

Here we present a model for the regulation of DNA methylation at telomeric regions based on our results and on the aforementioned previously reported data. This model has two consecutive steps. The first one involves the recruitment of the *de novo* methyltransferase DRM2 to an unmethylated telomeric region and the initiation of subtelomeric DNA methylation spreading (Figure 7A). The second one includes the reinforcement of this spreading and the maintenance of DNA methylation by all methyltransferases (Figure 7B). The first step is included because, considering current knowledge, the molecular pathways involved in the second one require the previous heterochromatization of subtelomeres. The first step starts with the recruitment of DRM2 to subtelomeric ITSs. DRM2 methylates ITSs and initiates the spreading of DNA methylation towards subtelomeres by methylating subtelomeric cytosines adjacent to ITSs in all sequence contexts. DRM2 is also recruited to alternative subtelomeric loci where it initiates spreading too. However, DRM2 do not spread DNA methylation towards telomeres. The perfect tandem telomeric repeat arrays of telomeres might directly or indirectly inhibit DNA methylation spreading from subtelomeric ITSs, which might be related to the recruitment of different TRFs by telomeres and ITSs (Figure 7A). Once spreading is initiated by DRM2, subtelomeric DNA methylation is further spread and maintained by all methyltransferases (Figure 7B). MET1 maintains most of the subtelomeric CGm independently of other methyltransferases, although its activity can be influenced by SUVH4/5/6, mainly SUVH5, through the dimethylation of H3K9. SUVH4/5/6, CMT3 and CMT2 contribute to reinforce spreading and maintain subtelomeric CHGm and CHHm through a positive feedback loop. This loop involves the recruitment of CMT3 and CMT2 by H3K9me2 as well as the recruitment of SUVH4/5/6 by methylated DNA. The activities of CMT3 and CMT2 increase at regions with high (CAG + CTG)d, where they are preferentially targeted by SUVH4, and decrease at regions with high CCGd, where they are preferentially targeted by SUVH5/6 bound to CG sites previously methylated by MET1. Since SUVH4 is the major H3K9me2 methyltransferase, CMT3 leads to higher levels of CAGm and CTGm than of CCGm. In turn, DRM2 leads to higher levels of CCGm than of CAGm and CTGm. However, both CMT3 and DRM2 preferentially methylate CCG sites previously methylated by MET1. DRM2 targeting to subtelomeres involves transcripts produced by POLIV and POLV. Whereas POLIV can be recruited by SHH1 bound to H3K9me2, POLV can be recruited by SUVH2 bound to CG sites methylated by MET1 and by SUVH9 bound to CHH sites methylated by DRM2 or CMT2. Both, POLIV and POLV, are recruited to specific subtelomeric locations and might act in concert with specific TRFs at ITSs.

In summary, the aforementioned model reflects that a complex network of interactions involving different self-reinforcing feedback loops govern subtelomeric DNA methylation. Although most of these interactions have already been described at a genome-wide level, there are some features of subtelomeric DNA methylation that, to our knowledge, have not been previously highlighted. Among those are the progressive drop of DNA methylation at the telomere-subtelomere boundaries, which supports the bimodal model of telomeric chromatin organization, the high levels of DRM2-dependent CHHm within subtelomeric ITSs and the strict dependence of subtelomeric CCHm on DRM2. In addition, our results have led us to propose that the methylation of the first cytosine within CCG sites by DRM2 is influenced by the prior methylation of the second cytosine by MET1, as has been previously proposed for CMT3, and that the activities of SUVH4/5/6, CMT3 and CMT2 on subtelomeric non-CGm increase with (CAG + CTG)d and decrease with CCGd whereas the activity of MET1 on non-CGm decreases with (CAG + CTG)d. In turn, the activity of MET1 on CHGm tends to increase with CGd/CCGd. Besides this, considering that we have analysed DMTs that are very short (40 bp), our results also support that the cooperation among methyltransferases to maintain subtelomeric DNA methylation might involve their physical interactions, which, in certain cases, have already been described (8,12).

Cytosine methylation has been related to a wide variety of biological features from the maintenance of genome stability to cell differentiation, development or illness (8). Since many of these features are also affected by telomere length and DNA methylation controls telomere length homeostasis in different organisms including Arabidopsis, understanding the molecular mechanisms that govern subtelomeric DNA methylation is an issue of significant interest (8–11). We have shown that Arabidopsis telomeric regions are an ideal system for the study of heterochromatic DNA methylation and its influence on telomere biology. Future studies should shed light on these issues and contribute to the development and refinement of the model that we have seed here.

## DATA AVAILABILITY

The TAIR10-Tel FASTA file is available and can be downloaded from the GitHub repository (https://github.com/NuttyLogic/DNA-methylation-at-telomeric-regions).

## Supplementary Material

gkac012_Supplemental_Files
