# Supplementary material for: A complex network of interactions governs DNA methylation at telomeric regions
Source: Nucleic Acids Res. 2022 Jan 21;50(3):1449–64. doi: 10.1093/nar/gkac012 (PMC8860613; doi:10.1093/nar/gkac012)
Supplement: gkac012_Supplemental_Files [file gkac012_supplemental_files.zip › Supplementary data.pdf]

SUPPLEMENTARY FIGURE AND TABLE LEGENDS

Supplementary Figure S1. DNA sequence modifications of TAIR10 to build TAIR10-Tel. The terminal sequences present in TAIR10 (>TAIR10 END) are shown together with the new terminal sequences present in TAIR10-Tel (>TAIR10-Tel END) for all telomeric regions except 2L and 4L, which contain ribosomal DNA. Changes made to the sequences of 1L, 3L, 5L, 1R, 2R, 3R, 4R and 5R are indicated with a color code. Black-labeled nucleotides remain in TAIR10-Tel as they are in TAIR10. Red-labeled nucleotides were added to TAIR10 to build TAIR10-Tel. Blue-labeled nucleotides were removed from TAIR10 to build TAIR10-Tel. In the case of 4R, we found that the end sequence present in TAIR10 is followed by long arrays of perfect telomeric repeats (TTTAGGG) interspersed with a specific type of degenerated telomeric repeat (TTAAGGG). In this case, ten TTWAGGG repeats (where W is A or T) were added to the end sequence present in TAIR10 to build TAIR10-Tel. In turn, ten perfect telomeric repeats represent telomeres in TAIR10-Tel in the remaining telomeric regions. These repeats are bold labelled.

Supplementary Figure S2. Chromosome ends in TAIR10-Tel. The end sequences of the telomeric regions present in TAIR10-Tel are shown. These sequences include the centromeric side of telomeres (70 bp) and the adjacent subtelomeric 2930 bp. Whereas forward sequences are shown for the left telomeric regions, reverse complementary sequences are displayed for the right telomeric regions. The color code used to highlight relevant sequences is indicated at the top of the figure.

Supplementary Figure S3. Cytosine methylation drops at the telomeric sides of the telomere-subtelomere boundaries. (**A**) Box plots showing the percentages of CHHm along the first 700 bp of telomeric regions after dividing them into 35 bp tiles. The distance of tiles to TAIR10-Tel ends is indicated. The first two tiles (35 and 70) are telomeric (Tel) and have significant lower levels of methylation than the rest of the tiles, which are subtelomeric (Subtel) ($p<0.001$). (**B**) Scatter plots showing the methylation levels of individual CHH cytosines along the first 210 bp of 1L, 1R, 3L, 3R, 5L and 5R. (**C**) Scatter plots showing the methylation levels of the first, second and third cytosines of the perfect telomeric repeats present at the inner part of telomeres, Tel (inner), at the telomeric sides of the boundaries, Tel (35) and Tel (70), and at the subtelomeric tiles shown in panel A (Subtel). Methylation values for the inner part of telomeres have been obtained from Supplementary Figure S4, assuming that most telomeric reads methylation occurs at the third cytosine of the perfect telomeric repeats. This figure has been performed using experiments SRR3384734, SRR5494752, SRR5494753 and SRR5494754 (WGBS set 2) and corresponds with Supplementary Table S6.

Supplementary Figure S4. Arabidopsis telomeres are not methylated. (**A**) Telomeric cytosine conversion levels in the experiments selected to analyse telomere-subtelomere boundaries. The number of reads, their length, the percentages of unconverted cytosines within telomeric reads (*A), which follow the telomeric (YYYTAAA)n pattern, and the percentage of telomeric reads containing one or more cytosines (*B) are indicated. (**B**) Number of telomeric reads containing from 0 to n Cs, being n the maximum number of Cs that these reads can contain. The information is supplied as follows: Experiment (total

number of telomeric reads) [nº of reads without Cs, with 1 C, with 2 Cs,…, with n Cs]. Telomeric cytosines conversion levels were determined as previously described in Vega-Vaquero et al. (2016) Genome Research 26:1047–1056.

Supplementary Figure S5. Subtelomeric DNA methylation profiles in methyltransferase mutants. Bars represent the mean levels of methylation within 40 bp tiles in the WT (red) and in the mutants (blue). The schematic representation at the bottom indicates the relative positions of telomeres and subtelomeres. This figure corresponds with Supplementary Table S8.

Supplementary Figure S6. DNA and histone methyltransferases interact at subtelomeres**.** (**A**) Principal components analysis of the different types of cytosine density and of CGm, CHGm and CHHm in the WT and in the methyltransferase mutants. Pearson correlation coefficients between the indicated variables (left column) and 3 principal components (PC1-3) are indicated. Correlation coefficients higher than 0.25 ($p<0.05$) and lower than -0.25 ($p<0.05$) are labelled in brown and blue, respectively. This figure corresponds with Supplementary Table S8. (**B**) Subtelomeric DNA methylation activities and interactions revealed by the principal components analysis. Black arrows indicate activities of methyltransferases on the different types of DNA methylation. Lines connecting proteins with arrows indicate positive influences of the proteins on the activities. The principal components related to the different activities and the contexts of cytosines that correlate positively with them are shown within grey rectangles at the bottom. White upside arrows next to each kind of cytosine density indicate high density levels whereas white downside arrows indicate low levels of density.

Explanation of the figure: This principal components analysis addresses how the different DNA methylation profiles relate among themselves and with the context of cytosines. PC1 shows a positive correlation with CHHm in WT, *met1*, *cmt3, suvh4/5/6* and *cmt2* but not in *drm2*, which reveals that it is related to DRM2 activity on CHHm. PC1 correlates positively with CHGm in WT, *cmt3*, *suvh4/5/6* and *cmt2* but not in *met1* or *drm2*, supporting that DRM2 can also establish CHGm and that this CHGm activity is influenced by MET1. In addition, PC1 has a positive correlation with CGm in *met1*. Considering that DRM2 can establish all types of DNA methylation, this result supports that DRM2 is responsible of the low levels of CGm remaining in the *met1* mutant. Importantly, all these activities of DRM2 are independent of the cytosine contexts. Thus, PC1 reflects DRM2 activity on CHHm and CHGm that is independent of the context of cytosines. PC2 doesn't correlate with sequence context. It shows a positive correlation with CGm in WT, *cmt3*, *suvh4/5/6*, *drm2* and *cmt2* and a negative correlation with CGm in *met1*, which reveals that it is related to MET1 activity on CGm. In addition, PC2 also has a positive correlation with CHGm in WT and *cmt2* but not in *met1*, *cmt3*, *suvh4/5/6* or *drm2*. Thus, PC2 supports that MET1 is the main CG methyltransferase at subtelomeres and positively influences CHGm in a CMT3, SUVH4/5/6 and DRM2 dependent manner. Since in *met1* CGm correlates positively with PC1 and negatively with PC2, DRM2 might slightly contribute to maintain CGm in the WT in regions with low MET1 activity. PC3 shows a positive correlation with (CAG+CTG)d and a negative correlation with CCGd. PC3 has a positive correlation with CHGm in WT, *met1*, *drm2* and *cmt2* but not in *cmt3* or *suvh4/5/6,* which reveals that it is related to CMT3 activity on CHGm and that this activity depends on SUVH4/5/6. In addition, PC3 also has a positive correlation

with CHHm in *drm2.* Therefore, the CHHm activity that remains in the absence of DRM2 correlates with the CHGm associated to SUVH4/5/6 and CMT3. This CHHm activity should be achieved by CMT2. Hence, PC3 supports that both, the activity of CMT3 on CHGm and the activity of CMT2 on CHHm are SUVH4/5/6-dependent, increase with (CAG+CTG)d and decrease with CCGd.

Supplementary Figure S7. Influence of methyltransferases on the DNA methylation profiles of the Ta3 retrotransposon. The positions of the Long terminal repeats (LTRs) are indicated. Bar plots represent the percentages of methylation at individual cytosines along At1g37100.1. CGm, CHGm and CHHm are indicated in red, yellow and blue, respectively. Whereas methylation of the forward strand is represented above the cero lines, reverse strand methylation is represented below. This figure correspond with Supplementary Table S9.

Supplementary Figure S8. DNA and histone methyltransferases cooperate to maintain DNA methylation within the Ta3 retrotransposon. (**A**) CGm, CHGm and CHHm levels within the coding region of Ta3 (At1g37100.1). Here and in the following panels bar plots represent the mean levels of methylation in the WT strain and in DNA methylation mutants, which are labelled with different colours. Significant differences of DNA methylation levels between the WT and the DNA methylation mutants are indicated with asterisks (*p<0.05, **p<0.01, ***p<0.001). (**B**) Influence of the cytosine context on CHGm. (**C**) Influence of the cytosine context on CHHm methylation. This figure corresponds with Supplementary Table S9.

Supplementary Figure S9. The influence of methyltransferases on DNA methylation overlap along the Ta3 retrotransposon. A classification of subtelomeric tiles according to their levels of methylation in methyltransferase mutants is shown. Wide yellow rectangles label tiles with levels of CGm, CHGm and/or CHHm below 20% in the WT strain. Whereas dark grey rectangles correspond with DMTs, light grey rectangles indicate the absence of differential methylation. The densities of CG, (CAG+CTG) and CCG sites are indicated in the right with different colours. The intensity of the colours (red, orange and blue, respectively) is proportional to the number of the sites, with white representing cero sites. This figure corresponds with Supplementary Table S10.

Supplementary Table S1. WGBS experiments used in this study. The characteristics of the WGBS experiments used to analyse subtelomeric DNA methylation in the wild-type strain (**A**) and in the mutants (**B**) are indicated together with the characteristics of the experiments used to analyse the telomere-subtelomere boundaries in the wild-type (**C**). WGBS set1 and WGBS set2 refer to the different sets of experiments used to analyse the boundaries. All the experiments have been previously analysed and validated, with the exception of SRR771524, which was not used in their original research [see Cell (2013) 152:352–364, Plant Physiology (2017) 175: 1893-1912 and G3: Genes, Genomes, Genetics (2016) 6: 2773-2780]. We estimated the background levels of methylation by determining the CHHm levels at subtelomeres between positions 2000 and 3000, which are out of subtelomeric heterochromatin, and by determining the levels of methylation of the chloroplast. The slight differences in chloroplast methylation between our analysis and those reported in the papers

are due to the fact that we are using more modern alignment tools (i.e. BSBolt; Farrel et al. Gigascience (2021) 10: giab033) that more accurately align reads compared to the approaches used in the previous papers (i.e. BS Seeker and Bismarck).

Supplementary Table S2. Statistical parameters. N, mean, standard deviation and p values corresponding to different Figures are shown in different tabs.

Supplementary Table S3. Distribution of perfect and degenerate telomeric repeats at Arabidopsis telomeric regions. The frequencies with which the CCCTAAA, TTTAGGG or any of these telomeric repeats containing one mismatch are found within the sequences of the telomeric regions displayed in Supplementary Figure S2 (1L, 3L, 5L, 1R, 2R, 3R, 4R and 5R) are indicated. The number of times that each telomeric repeat is found within all telomeric regions (in all TR) and all subtelomeric ITSs (in all ITS) is also shown. When a specific telomeric repeat is present in a precise subtelomeric ITSs its frequency is highlighted in brown. Telomeric repeats are ordered according to the number of occurrences in all ITSs.

Supplementary Table S4. Subtelomeric cytosine methylation in the Arabidopsis WT Col-0 ecotype. The methylation levels of individual cytosines between coordinates 71 and 3000 of 1L, 1R, 3L, 3R, 5L and 5R are shown. Hereinafter, coordinates correspond to the sequences displayed in Supplementary Figure S2. They indicate the distance to TAIR10-mod ends. Methylation levels were estimated using experiments SRR534177 + SRR534193 or SRR771524. Mean

methylation values for each cytosine were used for representations and analyses. Strand refers to the position of cytosines in the forward (1) or reverse (-1) strand, also according to the sequences displayed in Supplementary Figure S2.

Supplementary Table S5. Tiled subtelomeric cytosine methylation in the Arabidopsis WT Col-0 ecotype. The percentages of methylation and the number of cytosines in different sequence contexts within 40 bp tiles are shown. Tiles group cytosines between coordinates 71 and the right borders of subtelomeric DNA methylation at 1L, 1R, 3L, 3R, 5L and 5R. They are numbered according to their last nucleotide. Cm refers to the average methylation of all cytosines within a tile. ITS (Y/N) refers to the presence (Y) or absence (N) of telomeric repeats within a tile. Percentages of methylation were estimated using experiments SRR534177 + SRR534193 or SRR771524. Mean values (mean) were used for representations and statistical analyses.

Supplementary Table S6. Cytosine methylation at the telomere-subtelomere boundaries of the Arabidopsis WT Col-0 ecotype. CHHm levels of individual cytosines between coordinates 1 and 700 of 1L, 1R, 3L, 3R, 5L and 5R are shown. Cytosines are grouped in 35 bp tiles, which are numbered according to their last nucleotide (Tile). When cytosines are part of perfect telomeric repeats (CCCTAAA) their positions in the repeats are indicated (1, 2 or 3). CHH methylation levels were estimated using experiments SRR771524 (WGBS set1) or SRR3384734 + SRR5494752 + SRR5494753 + SRR5494754 (WGBS set2).

Supplementary Table S7. Subtelomeric cytosine methylation in the Arabidopsis WT strain and in methyltransferase mutants. Individual cytosines between coordinates 71 and the right borders of DNA methylation at 1L, 1R, 3L, 3R, 5L and 5R are included. The context of cytosines, their coverage and their methylation levels are shown together with their position in the forward (1) or reverse (-1) strands, according to the sequences displayed in Supplementary Figure S2.

Supplementary Table S8. Tiled subtelomeric cytosine methylation in the Arabidopsis WT strain and in methyltransferase mutants. Cytosines between coordinates 71 and the right borders of subtelomeric DNA methylation at 1L, 1R, 3L, 3R, 5L and 5R were grouped in 40 bp tiles and analysed. Tiles are numbered according to their last nucleotide. Cm refers to the average methylation of all cytosines within a tile. ITS (Y/N) refers to the presence (Y) or absence (N) of telomeric repeats within a tile.

Supplementary Table S9. Cytosine methylation within the coding region of the Ta3 retrotransposon (At1g37100.1) in the Arabidopsis WT strain and in methyltransferase mutants. Individual cytosines between coordinates 14130454 and 14134521 of chromosome 1 are included. The context of cytosines, their coverage and their methylation levels are shown together with their position in the forward (1) or reverse (-1) strands.

Supplementary Table S10. Tiled cytosine methylation within the coding region of the Ta3 retrotransposon (At1g37100.1) in the Arabidopsis WT strain and in

methyltransferase mutants. Cytosines between coordinates 14130454 and 14134521 of chromosome 1 were grouped in 40 bp tiles and analysed. Tiles are numbered according to their last nucleotide.

Supplementary Figure S1

1L

>TAIR10 END

CCCTAAACCCTAAACCCTAAACCCTAAACCTCTGAATCCTTAATCCCTAAATCCCTAAATCTTTAAATCCTACATCCA
TGAATCCCTAAATACCTAATTCCCTAAACCCGAAACCGGTTTCTCTGGTTGAAAATCATTGTGTATATAATGATAATT
T

>TAIR10-Tel END

CCCTAAACCCTAAACCCTAAACCCTAAACCCTAAACCCTAAACCCTAAACCCTAAACCCTAAACCCTAAACCCTAAATAAAG
CGCTGTGGGATCAATCATTGCATTTTTCCATAGGATAGATAGGGCCGACAAGATCATCAGGGAAGAAGTCAAATCA
CATCCGAATTCAATTGTTCTTTTCCTAAACCCTAAACCCTAAACACTAAACCCTAAACCCTAAACCCTAAACCCTAAA
CCTCTGAATCCTTAATCCCTAAATCCCTAAATCTTTAAATCCTACATCCATGAATCCCTAAATACCTAATTCCCTAAAC
CCGAAACCGGTTTCTCTGGTTGAAAATCATTGTGTATATAATGATAATTT

3L

>TAIR10 END

NNNNNNNNNNNNNNNNNNNNNNNNNNNNNNNNNNNNNNNNNNNNNNNNNNNNNNNNNNNNNNNNNNNNNNNNNNNNNNNN
NNNNNNNNNNNNNNNNNNNNNNNNNNNCCCTAAACCCTAAACCCTAAACCCTAAACCCTAAACCCTAAACCCTAAA
CCCTAAATCCATAAATCCCTAAAACCATAATCCTAAATCCCTTAATTCCTAAATCCCTAATACTTAGACCCTAATCTTT
AGTTCCTAGACCCTAATCTTTAGTTCCTAGACCCTAAATCCATAATCCTTAATTCCTAAATTCCTAAATCCCTAATACT
AAATCTCTAAATCCCTAGCAATTTTCAAGTTTTGCTTGATTGTTGTAGGATGGTCCTTTCTCTTGTTTCTTCTCTGTGT
TGTTGAGATTAGTT

> TAIR10-Tel END

CCCTAAACCCTAAACCCTAAACCCTAAACCCTAAACCCTAAACCCTAAACCCTAAACCCTAAACCCTAAACCCTAAACCCTT
AACCCTTAACCCTTAACCCTTAACCATAAACCATAAACCATAAACCCTAAACCCTAAACCCTAAACCATAAAACCATA
AACCCTTAAACCCTAAAACCATAAAACCATAAACCATAAACCATAAACCCTAAACCCTAAACCCATAAAACCATAAAA
CCATAAATCCCTAAAACCATAAAACCATAAATCCCTAAACCCTAAATCCCTAAACCCTAAATCCATAAATCCCTAAAA
CCATAATCCTAAATCCCTTAATTCCTAAATCCCTAATACTTAGACCCTAATCTTTAGTTCCTAGACCCTAATCTTTAGT
TCCTAGACCCTAAATCCATAATCCTTAATTCCTAAATTCCTAAATCCCTAATACTAAATCTCTAAATCCCTAGCAATTT
TCAAGTTTTGCTTGATTGTTGTAGGATGGTCCTTTCTCTTGTTTCTTCTCTGTGTTGTTGAGATTAGTT

5L

> TAIR10 END

TATACCATGTACCCTCAACCTTAAAACCCTAAAACCTATACTATAAATCTTTAAAACCTATACTCTAAACCATAGGGTT
TGTGAGTTTGCATAAAGTGTCACGTATAAGTGTTTCTAACATGTGAGTTTGCATAAGAGTCTCGACTATGTGTTTGTT
CAAAAGTGACGTAAGTGTTTAGACTAGAGCCGGCCGTGAGCACAAGCGGGCCAAGCCCATGCTTGCGGCAGATTA
TCCTATAATTATGTTTTGCGGCTTTACAATTTTGAATTTGTTTTGTTGTTTAGTGGTCGAGTCAGGGATGAGTTTGTTC
TCAAACATCTCAAATTTCTAATCTTCCAAGTGTTAGGTTCACTCTACCTTATTTTTTTTTTTTTTTTTTTATTTTAAATTTC
TATTAGAAAGAACTTGAACCTTATTTCAAGTGAACATTAAAAAGAATTGTAACCCTACCTTATTTCTATTATTATGAAT
ATGTGTAAA

> TAIR10-Tel END

CCCTAAACCCTAAACCCTAAACCCTAAACCCTAAACCCTAAACCCTAAACCCTAAACCCTAAACCCTAAACCCTAAACCCTA
TACCATGTACCCTAAACCCTAAACCCTAAACCCTAAACCTATATAGTATACCCTATACCATGTACCCTCAACCTTAAA
ACCCTAAAACCTATACTATAAATCTTTAAAACCTATACTCTAAACCATAGGGTTTGTGAGTTTGCATAAAGTGTCACG
TATAAGTGTTTCTAACATGTGAGTTTGCATAAGAGTCTCGACTATGTGTTTGTTCAAAAGTGACGTAAGTGTTTAGAC
TAGAGCCGGCCGTGAGCACAAGCGGGCCAAGCCCATGCTTGCGGCAGATTATCCTATAATTATGTTTTGCGGCTTT
ACAATTTTGAATTTGTTTTGTTGTTTAGTGGTCGAGTCAGGGATGAGTTTGTTCTCAAACATCTCAAATTTCTAATCTT
CCAAGTGTTAGGTTCACTCTACCTTATTTTTTTTTTTTTTTTTTTATTTTAAATTTCTATTAGAAAGAACTTGAACCTTATT
TCAAGTGAACATTAAAAAGAATTGTAACCCTACCTTATTTCTATTATTATGAATATGTGTAAA

1R

> TAIR10 END

ACAATCAGTGAAAACTGCTTCGAGTTTTGCGGATGCTGCAATGGGAAGAATTGCACAGAGTACAAAGGTTCTGACA
AAAGGAGGATATGAGAAAATTTTCAGACAAACCTTTGAAACTATTCTAGAGGAGCAGCTGCAGAATTCATTTGCGTG
CTACTTATCAACATCAGCTGGTCCAGTCATGGGAGTTCTTTACGTATCCACTACTAAACTTGCTTACTCAAGCGATAA
CCCTCTCTCGTACAAAAATTCTGGTCAAACTGAATGGAGCTATTATAAGCTAATCTTCTTTATTTGAGACAATCGAAC
TTTTTGCTATAAATAACTATTGTAGAAAGCGACCTCATAGATGGTAGGAATAGTGTGAAGAACGTTAGATTCTGTGAC
TAATTTCTTGTGCAGACTATAATAACACTGGGTAGTAATACTTATATCTTCATCATCTTAAACATGTAGTAATCCCATT
ACATCAGCTTAAATCGGTTAACCCTTCTACAAACACAGTCAATCCTGCAGAAAGGTACATCCAGATAATCTCAGTCG
ACGACCATGAGTTTTGGTTCATGTGTTTCTTAAACTACTGTGTGATATAATATTGGTGGATTCTTGATTAGAATAACA
TTATTTTGGTTGCAAACTAAATTATTTGATTCTTATTCTTAATTAGTTACCATGTCTTGATTAGGGTTTAGGGTTTAGG
GTTTAGGGTTTAGGGTTTAGGG

> TAIR10-Tel END

ACAATCAGTGAAAACTGCTTCGAGTTTTGCGGATGCTGCAATGGGAAGAATTGCACAGAGTACAAAGGTTCTGACA
AAAGGAGGATATGAGAAAATTTTCAGACAAACCTTTGAAACTATTCTAGAGGAGCAGCTGCAGAATTCATTTGCGTG
CTACTTATCAACATCAGCTGGTCCAGTCATGGGAGTTCTTTACGTATCCACTACTAAACTTGCTTACTCAAGCGATAA
CCCTCTCTCGTACAAAAATTCTGGTCAAACTGAATGGAGCTATTATAAGCTAATCTTCTTTATTTGAGACAATCGAAC
TTTTTGCTATAAATAACTATTGTAGAAAGCGACCTCATAGATGGTAGGAATAGTGTGAAGAACGTTAGATTCTGTGAC
TAATTTCTTGTGCAGACTATAATAACACTGGGTAGTAATACTTATATCTTCATCATCTTAAACATGTAGTAATCCCATT
ACATCAGCTTAAATCGGTTAACCCTTCTACAAACACAGTCAATCCTGCAGAAAGGTACATCCAGATAATCTCAGTCG
ACGACCATGAGTTTTGGTTCATGTGTTTCTTAAACTACTGTGTGATATAATATTGGTGGATTCTTGATTAGAATAACA
TTATTTTGGTTGCAAACTAAATTATTTGATTCTTATTCTTAATTAGTTACCATGTCTTGATTAGGGTTTAGGGTTTAGG
GTTTAGGGTTTAGGGTTTAGGGTTTAGGGTTTAGGGTTAAGGGTTTAGGGTTAAGGGTTTAGGGTTAAGGG**TTTAG**
GGTTTAGGGTTTAGGGTTTAGGGTTTAGGGTTTAGGGTTTAGGGTTTAGGGTTTAGGGTTTAGGG

2R

> TAIR10 END

TATAGGGTATAGGGTATAGGGTATAGGGTATAGGGTATAGGGTTTAGGGTTTAGGGTCTAGGGTTTAGGGTTTAGG
GTTTAGGGTTTAGGGTTTAGGGTTTCGGGTTTAGGGTTTAGGGTTTAGGGTTTAGGGTTTAGGGTTTAGGGTTTAG
GGTATAGGGTTTAGGGTTTAGGGTTTAGGGTTTAGGGTCTAGGGTTTAGGGTTTAGGGTTTAGGGTCTAGGGTTTA
GGGTTTAGGGTCTAGGGTCTAGGGTTTAGGGTTTAGGGTTTAGGGTCTAGGGTTTAGGGTCTAGGGTTTAGGGTTT
AGGGTTTAGGGTCTAGGGTTTAGGGTTTAGGGTTTAGGGTTTAGGGTCTAGGGTTTAGGGTTTAGGGTTTAGGGTT
TAGGGTTTAGGGTTTAGGGTTTAGGGTTTAGGGTTTAGGGTTTAGGGTTTAGGGTTTAGGGTTTAGGGTTTAGGGT
TTAGGGTTTAGGGTTTAGGGTTTAGGGTTTAGGGTTTAGGGTTTAGGGTTTAGGGTTTAGGGTTTAGGGTTTAGGG
TTTTAGGGTTAGGGTTTAGGGTTTAGGGTTTAGGGTTTAGGGTTTAGGGTTTAGGGTTTAGGGTTTAGGGTTTAGG
G

> TAIR10-Tel END

TATAGGGTATAGGGTATAGGGTATAGGGTATAGGGTATAGGGTTTAGGGTTTAGGGTCTAGGGTTTAGGGTTTAGG
GTTTAGGGTTTAGGGTTTAGGGTTTCGGGTTTAGGGTTTAGGGTTTAGGGTTTAGGGTTTAGGGTTTAGGGTTTAG
GGTATAGGGTTTAGGGTTTAGGGTTTAGGGTTTAGGGTCTAGGGTTTAGGGTTTAGGGTTTAGGGTCTAGGGTTTA
GGGTTTAGGGTCTAGGGTCTAGGGTTTAGGGTTTAGGGTTTAGGGTCTAGGGTTTAGGGTCTAGGGTTTAGGGTTT
AGGGTTTAGGGTCTAGGGTTTAGGGTTTAGGGTTTAGGGTTTAGGGTCTAGGG**TTTAGGGTTTAGGGTTTAGGGTT
TAGGGTTTAGGGTTTAGGGTTTAGGGTTTAGGGTTTAGGGTTTAGGG**

3R

> TAIR10 END

AATTAAAGGATCACAAAACAAACTCCAGTTTATGTATCCCATTCTCGTTCTCACCAAATTAAATACATTTCGACAAGC
AAACATTGAAATTTCAAGAAGAAGAGACAGTCTTGAAGGAGTCGCGAAGGTGGGTTAATGTATCGTGGTCGCGGTT
CCACTCGGGCGCAGGGGCGTTTACGAAATGGGCGTAGAGCTTGTTCTTGGAGCAAGTGACTGTGATCAGGCTGTG
CTTTCCAACCCCATCTATCTCGTACAGATAGTAGACCTGCCCTGTCTCGTCCTTCCTCTCCTCCGACACCACTTTGT
CCGCGCCGGCTATCAAATCCTGCAGCTCCACGTCCGACAAGGCAAGGTTGTTCAGAATCACATCCCTCGGCGCCA
GCTGTCTGAATCCCGCCAGGAATGTCAGGTACTCCTTCTCCGTCTTCTTCTTCGGGTTATAGAATTCGCTGTCTGTT
CCGTTTGTCCCCTTCTCCACCTTCGACACCAGCCTCTCCTTCCACCCTTCCTCTACTTCTACCCTAAACCCTAAACC
CTAAACCCTAAACCC

> TAIR10-Tel END

AATTAAAGGATCACAAAACAAACTCCAGTTTATGTATCCCATTCTCGTTCTCACCAAATTAAATACATTTCGACAAGC
AAACATTGAAATTTCAAGAAGAAGAGACAGTCTTGAAGGAGTCGCGAAGGTGGGTTAATGTATCGTGGTCGCGGTT
CCACTCGGGCGCAGGGGCGTTTACGAAATGGGCGTAGAGCTTGTTCTTGGAGCAAGTGACTGTGATCAGGCTGTG
CTTTCCAACCCCATCTATCTCGTACAGATAGTAGACCTGCCCTGTCTCGTCCTTCCTCTCCTCCGACACCACTTTGT
CCGCGCCGGCTATCAAATCCTGCAGCTCCACGTCCGACAAGGCAAGGTTGTTCAGAATCACATCCCTCGGCGCCA
GCTGTCTGAATCCCGCCAGGAATGTCAGGTACTCCTTCTCCGTCTTCTTCTTCGGGTTATAGAATTCGCTGTCTGTT
CCGTTTGTCCCCTTCTCCACCTTCGACACCAGCCTCTCCTTCCACCCTTCCGGCACATCGTACACGTACTCCGCCG
ATGTCTTCCTGTCTGAATTCCCCCCGTATCCTCCGTAGTTCGCCGCGCTCGCCGCCGTCCCGTAGTAAATTTTGTA
CGTCTGGTTCCCCTCGCCTGCCGCCGGCAGCACCGGAACCATAGTCAGCACGGAAGTCGCGGCCAACGCGGCGG
CCAGGGAGGTGCCTAGACGTGATGCTATCGCCTTTGGGACGATCGGCGTTCTGGTGGCGGAGATAACGGGAGAT
GCCAAGAAGATTTTCGTGGCCATTATACTCTCTATGGATAACGTTTTCCCAGCTCCCTTCTTGTGTCTTATTTATTAC
AACCTTCTACTTGTGTCAGCGGGTTTGATTTTTGCTGAATAAATAAAATCTCCGGATAAGCCCCACAGTTTATCATAT
TTGCATGTACACCCCGGGATTCCCAATTAAGCCGGACTCTCAATCCGCGAAACCCTAAACAATCATCTTTTTTTATT
CCCAGGAGTCGAGAGATATATTCATTCATAATCAGCCCCCGGATTCTTCTCTCGATCGCCGATGTCGAAAAGGTGC
GTGCTTTACCCATTTCTCTTCTTCATAGGCTGACTTCTTTGTCTGAAAGCTTGCAACCTTTTTTAGTTTTAATTACAAA
TTGATACGAGTAATGTTCTTATATTGATACGTCTCTGCACATGAACCATCTTGTACCAATGAATATAATCTCAGGGTG
TGGGCTCTTATAATTTCTTCATCCACGGGGACAACGTTTAGACTTGAGAGTAATTTATCATGAATTCGCCATCTGGA
AAGATCTTTATCACTGTTTTTTCGTAAATCTAAGCGTGTGATAATTTTCAGTCAAGAATCCGTGAATAGTTTCATCTTC
TGTATGCTTAGTTGCATTCCTTGTTCATTGATGCTCTTTTTGTATTTTGTTTCTTCTCAACTTTTTCTTCGTTTTCTTTT
GTTTTCTCTTAGAATCCTTTGTAAATTCTTTGCTCACGGTGCATGCTTGAAAGGGGAGCACTGTGAATTTTCTCATGA
CTGGAAGGATCCTACAAATAATGTAAGCTTTAACTCTCTCCCCTATGTATGGGGGAACCAGCTTTCATCAACTTTAA
ATTCCTTTGTATTGTTAATGTTCTCTAAGAAACGACCATCCTATCACAGATTTGCACCTACTACCAGAAAGGAATCTG
CTCTTACGGAAGTCGATGCAGGTACGAACATGTCAAAGCTTCCAGGCCTCATCCATCTGCTTCATCTTCGTCCGCT
GTGCCTCGGCCATCTCCTGCTTCAGCTTCCAACACTCTTGCCTGTGCTTTTCTTCCTGGTGTCGCGGAAAGAGATTT
GAGCCCAGTTCCTTCTTCATCTTTAAAACCAACGTGGAATTTGGATTCTCTTCACCAGGATTCCGTAGACGAGGTCA
AAACTTCTAACCCTGGTACAGTCAAACCTGAAGAACTACCCATCTGCTCATTTGCTGCTGCTGGTGATTGCCCACGT
GGGAATGAATGCCCTCACATCCATGGAACTATTTGCCCAACTTGTGGGAAATGTTGCTTGCATCCTTTCAGACCCG
ACGAAAGAGAGGAGCACAAGAAAGTTTGTGAAAAAAAGCACAAGCAACTTGAAGCATTAAAACTTAGCCAAGAGAT
CGAGTGTTGTGTCTGTTTGGAACGTGTATTGTCTAAGGCAACTCCAGCTGAACGCAAGTTCGGGTTACTCACTGAA
TGTGATCATGCTTTCTGCATTGCATGTATCAGGAATTGGCGGAGCAGTTCTCCTTCCACTGGGATGGATGTCAATAG
CACACTAAGGGCTTGCCCCATATGCCGCAAGTTATCGTATTTTGTTGTTCCTAGTGTAATCTGGTTTTCAGCTCCAG
AAGAGAAAAAAGAAATTATGGATAACTACAGGGAAAAGCTCAGGTACATGCTTTATGAACATTGCCTAAGTTAACTA
ACCATGGGCAAGGTTTTTCATTCTGGTGGTTTTATTGCTGTCTTTGTGTTCATCATACTTGATTGGCAAACAAAGCAA
GAAAAATCTGTATACTTATAATCAATTTTATGCTCTGTCTACCAGGTCAATCGATTGTAAACACTTTAGTTTTGGAGAT
GGGAATTGTCCATTCGGGACAAGTTGCTTCTACAAGGTATTTTTATGCATTCAGGGTTGAAACTCTAACTAAATCAA
GCATGCTCGGATGATGTTAAATAATATGTGTTGATGTTTTTTACTGATAGTGACAGGCAATCCCTTTTTAGTCGGGG
GTGTATTTAAAACCATGCTGGTTGGATTAATTTTGGTTCTTAGTTTCTTTGAGGAGCCTTTGGTTGAAATGAGTTCAA
AAACTTAATTGTTTAAGTAGACATTTGGCACTGCTGTTGAAGATTACATTGAAATCAGGTTTCATTGTATGTATTTACT
GCCTTGATTTCATACATGAGTATTTTCTGGTATCTGTTTGAAACCAATATTGAATCACCATATCTGAAGTTCTCTTGAA
AGTTGAAAATCTAGTTATATATCTCATACTTGAGATGACGTCAACTATTAAACCTTGATGTCTCTTACTGATAGGCAA

TTCCTGCTTTTTTACATTTCTTTTGTGTTTCTATATCTGAGCTTACCTATCTATACTACTTATTGCCAGATGTTCCGAAT
CTAGAAAATTTAAAATGTTTGCTGATAACCGCAGCGTTGAAGCTTTATAATTATCCAAGAGTCTTGGCAATTAGTTTA
CATAAATTTTTCAGCTAATATTTTATGTCACTATAGAAGGAAGTGTAGAAAATTGATATAATGTGATATAGCATCAATG
GTTAGTGATATTCAGTAGGGAGGGTGGATTTGCAGAATTTTCTTCTAAGTCCTTAGAAAGCTTGATTAGTCCTTAATG
GATGTTGTTGTGTTCCAGCACACTGTGAAGCCAGGTTCATATGCATGGAAATATCACAGGCCACCTCCAAGGCGTC
CACGTCCCTATGGAACTAACTTCTCAGACGTGGATACACTTGTAAATATGATCGGGAATATAATTTCAGAAGGTGAA
TATGGTCCTATTGGGTTTAGAGATACTGACGATGACGAACTGACTACAACGGATATGATGATGTTACTGATGCACAT
GGATATCGAATCTGAGGACTCGTCCAATGAGGATAGCTATTAGCTACCCATATCGAAGTTAGTTAACTAAAGTGAGA
GTCTTAGGTTATATGGCTATATTATGGTGTTGGGAGTTCGTAGTCGTTTTTTGTCTTTTGAGTGAAGCATAATTTGAC
TATTTTCCTCTTGAAATGTATATTTGTTGGTGTTGATATGGATGCACCTCTAAAACTCAAAAGGGGGGTTGTGGGTG
TGATTGCAGCATGCGTATCATGATGGTCGTTTGGAGGAAGTGGTTTTGCGGCATCTGGATGCAGAAGATGGGCAG
ACTGTGATAGCAAAGGATATAAGGTATTGGTGCCTTCAACTTGTACCACTAGTCTCTTGTTTCAATTGATTTGAGCTT
ACCTCTGGATGGTTATTCGTCTTTATAGGTTGTCCGACTTCCTTGAGAACATGCACATATGAACAGAGTCTCTCTTA
GCACAATAAGGGAGAAGGCAAAGATGTTGTGATGACATCTTTTTTTCTTCTTCCTGGGGTGTTTCCCATTCATTGTA
ACGATTTGATTTGAGTTGGAAGATTATGTCTTAGACCCCAACGCATTATGACTTGCGTTAAGAGGGGATCATTTTATT
AATTTTGTATTTTTAACAAATATCACTACTTCCTGCAAATCAACGCACATGAGCTATACAGATGTAGTAACGTGGGGC
TATAATATTTATCAAAATCCTTTGGATTGCTTACCTGTCCTTCTGTTACCAACTGGAACTACCGTTTCACTTTCACCTA
TATCATCGTCTTATTCATCTGCTTAGTTGCTTTCCCGACAACTCAACTCCTTTAGTGCTAATTGTTACATTGCGTATAT
GCAATCTCTGATAGCTGTTTATTTCTTTTTAAGTAAATTGCGTATATTCAATCTCTGATAGCTGTTTATTCCTCATCCA
AAGTACGTACTCTTGTTACTTGCGTCTAAACAACCTAATAATTATTTTATTTTATTCTTTTTGACAAGCTATATTTTTCA
AAAACATTGTAGTTTTCACAAATTTTAAACTATTTCAATGTTTTTCTTTCAGATATCGTGTCATTTTTATTTGGTACATG
TTATTTATTAAAATTACTAGCACATGTGTCTTCTTTATATATCTCATATGTAACTTCCGGTATTGCATTCATAAATCTTG
ACAACACCACCTTCATCCAAATTTGTAACTTTGCTACTTGTTTCATTTTTTTATCTTTCACTGTGACCTCTCCCTCCAT
ATTTACCATCATCACCACTTTTTGAATTGTAATTTTAGTTATCACTACAATTCTATGATCTATTGTTTTGTACTTACTAT
GATCTAAAAAAACAATTTGATATCTATCTTACATCTACCCCCACATGAAAAAGAGTTAGTTTGCAAAGAAAATTGATA
CAAACGTTAGTTTCTTAATTATAGTGTAAAATTGCTATCCATTAATATTATCTAAAAAAAATTACAAAGATCAATTCAAT
CCCCCACTATCTTGTTTTGGTCTTAGATGTCATAATGTTTAAAAATTTATTTATAGGAGTTATATGGATTTAAAAACGC
ACTGTTTATCTATTTTTCATGTAATGATGGGCCTAAATTTGAAGTCTGATATTAAACGAGTCCATTCTTCACCGACAT
GTCATTGTTCTGTTACATTTGGGTACCTTGGTTTATGTAAAATGACAGTGACGGGAGCCAAGCAGAAAGTAAAACGG
CGGTTGTTGCGGCGACTCCTTTAACAGCAACGACGTCACCTTTCAACAAAAGTGGCGACTCTTTTTAATAATTTAAA
CTCCGTCGACGTCTATTTTGGAGTCGCAGAAAGTGACCGCTGTGACCACGTGCCGAACATGGTAATTATATTATGT
ATAATGATGTGATAAAATAAAATGGTCATTGTAAAAGCATATTTTAAAAGATGTTTTCCCATTTACCGTCAACCTATTC
CTTATGTAGTCTTGAAAGTGATTAATTTTTTAGCACATCTTGAGTCATCTCAAAGTGAACACTGTAATTTGAGGGAGG
TGATTTTGACGGTTCTTGTAAACTTAAATTATACCATCCAACACTCTTAAAAATTTGAGTCTTCTTAAAAAATTTAACC
ATAAAATAAACCAAAGTTTAGAATAGTTCGTAAATGAACTAAATACATTAGAATGGTTTAACTAAACGGTTACAAAAAC
TTATAGCCTGGACGCTTCATTGCTTCAGTTTTCTCCAAAAACCAGACGAAGGCAAACCTATCCAATCCCTAAACCGT
CACATTTTGGCGCCAATTTTCATCTTCCTCATCATGGACTCCGATTCCGCCGGAGCCACAATCTCCGGCAACTTCCC
AAACTCCGACAACACAGAAACCCTAAATCTCAACACTACGAAACTCTACTCATCAGCAATTTCCTCTATCTCCCCAC
AATTCCCCCCACCGAAACCAACATCTTCATCTCCTTCAATCCCTAATTCAAAACGAATCCCTAAAACTAACTTCATCG
TCGATCTCTTCCGCCTCCCACACCAATCCTTCTCCGTCGCTTTCTTCCTCTCACATTTCCACTCTGATCACTACTCC
GGTCTCTCCTCTACTTGGTCTAAAGGAATTATCTACTGCTCACACAAAACCGCTCGTCTCGTTGAAGAAATCCTTCA
AGTTCCTTCACATTTCGTCTTCGCTTTACATATGAACCAAATGGTTAAGATCGATGGCTTTGAAGTTGTATTGATTGA
AGCTAATCATTGTCCAGGAGCGGTACAGTTGAGATTTGATTCGTTTCTTAATGGTTTTGTTGGTTGTGATGGTGTTTT
TCTGGATACTACTTATTGTAATCCTAAGTTTGTTTTCCCGAGTCAAGAAGAGTCTGTTGATTATGTGGTTATGTGATA
GATAAAATTAGTGAGGAGAAGGTTTTGTTTCTTGTAGCTACTTATGTTGTTGGTAAAGAGAAGATTTTGGGTGAGATT
ACAAACTTAGGATTACAATAAGTAATAATAGGG**TTTAGGGTTTAGGGTTTAGGGTTTAGGGTTTAGGGTTTAGGGTT
TAGGGTTTAGGGTTTAGGGTTTAGGG**

4R

> TAIR10 END

TTCTCCTAATCATATTATTGCTAATAAAAAATATAATTGGATTTTAGGATTTAATGCTGTTTTGAGATGATAGTGTATG
GTTGTAAAACAAGATTTGGTTGTTACTCTTACAATCTCCATCGCACTAGGTGATTAGCAAACATAACTCTTCTTTCAT
TCCCTTTTAAATAGAATTTGCAAGAAGAAAAAGTGCAAACATAGATAGATATCATAGAAAGAGAGAGACAGTAGGAG
AGATAGAGTGAAAAATAGGGTTTAGGGTGAGGGATAAGGTTATAGGGATTTAGAGATTTAGGGGTTTAGGTTTAAA
GGTTAAGGGTTAAGGGTTAAGGGTTAAGGGTTAGGGGTTAAGGGTTAGGGGTTAAGGGTTAAGGGTTTAGGGTTA
AGGGTTAAGGGTTTAGGGTTTAGGGTTTAGG

> TAIR10-Tel END

TTCTCCTAATCATATTATTGCTAATAAAAAATATAATTGGATTTTAGGATTTAATGCTGTTTTGAGATGATAGTGTATG
GTTGTAAAACAAGATTTGGTTGTTACTCTTACAATCTCCATCGCACTAGGTGATTAGCAAACATAACTCTTCTTTCAT
TCCCTTTTAAATAGAATTTGCAAGAAGAAAAAGTGCAAACATAGATAGATATCATAGAAAGAGAGAGACAGTAGGAG
AGATAGAGTGAAAAATAGGGTTTAGGGTGAGGGATAAGGTTATAGGGATTTAGAGATTTAGGGGTTTAGGTTTAAA
GGTTAAGGGTTAAGGGTTAAGGGTTAAGGGTTAGGGGTTAAGGGTTAGGGGTTAAGGGTTAAGGGTTTAGGGTTA
AGGGTTAAGGGTTTAGGGTTTAGGGTTTAGGG**TTWAGGGTTWAGGGTTWAGGGTTWAGGGTTWAGGGTTWAGG**
GTTWAGGGTTWAGGGTTWAGGGTTWAGGG

5R

> TAIR10 END

TTTATTCTAAACTAGAGCCGTCCCTATGATTTACAAGGCTATAAGAAAAGAACAAAAGTAGGTCACTTTATACATGTT
TATTGTAAAAAAGAATTACTTTACACGTATTCATAATAATAGAAATAAGGTAGAGTGACAATTCTTTCTAATGTTCATT
TGAAATAAAGTTCAAGTTATTTCTAATGGAAATTTAAACTTAAAAAATAAAAATAAAATAAAGTAGAGTGAACCTAACA
TTTTGGAAGATTTGAATTTGAGATGTATGAGAACTAACTCATCTCCGACCCGACCACTTAACCACAAAACAATTTCAA
AATTATGAAGCAGCAAAACATAATTATATGATAATCGGCCGCAAGCATGGGCTTGACCCGCTTGTGCTCACGGCCG
GCTCTGGTCTAAACACTTACGTCACGTTTGAACAAACACATAGTTGAGACTCTTATGCAAACTCACATGTTAGAAAC
ACTTATACAAGACACTTTATGCAAACTCATAAACCCTACGGTTTAGAGTTTAGGGTTTAGGGATTTAGGGTATAGGG
TATAGGGTATAGGGTATATGGTATAGGGATTTAGGGTATGGTATAGGTATATGGTTTAGGATTTAGGGTTTTTAGAT
C

> TAIR10-Tel END

TTTATTCTAAACTAGAGCCGTCCCTATGATTTACAAGGCTATAAGAAAAGAACAAAAGTAGGTCACTTTATACATGTT
TATTGTAAAAAAGAATTACTTTACACGTATTCATAATAATAGAAATAAGGTAGAGTGACAATTCTTTCTAATGTTCATT
TGAAATAAAGTTCAAGTTATTTCTAATGGAAATTTAAACTTAAAAAATAAAAATAAAATAAAGTAGAGTGAACCTAACA
TTTTGGAAGATTTGAATTTGAGATGTATGAGAACTAACTCATCTCCGACCCGACCACTTAACCACAAAACAATTTCAA
AATTATGAAGCAGCAAAACATAATTATATGATAATCGGCCGCAAGCATGGGCTTGACCCGCTTGTGCTCACGGCCG
GCTCTGGTCTAAACACTTACGTCACGTTTGAACAAACACATAGTTGAGACTCTTATGCAAACTCACATGTTAGAAAC
ACTTATACAAGACACTTTATGCAAACTCATAAACCCTACGGTTTAGAGTTTAGGGTTTAGGGATTTAGGGTATAGGG
TATAGGGTATAGGGTATATGGTATAGGGATTTAGGGTATGGTATAGGTATATGGTTTAGGATTTAGGGTTTTTAGAT
CCGTGAGACTGGTCAAGCTTTCGATCGCCTCGGTTGTCGTCTTCAAGTTAAAAAAATTTAACATCATGTTATATATCT
TAAATTTGTTACTATTTTCCTTTCTCTTTTTTCCTTGACCAGGAGACTTAATCTAAATTTTTTGTTAATGTCTTTACTTA
CCATCCATATTTAAATTAACTATCATTTCTTATATCTCTCTCGTCATTTAATTCAGGACGTGTGAAACAGAAACTACAA
GTGATATATATGAAACTAGATGTGATATGGAACTCATGATGTGCAACTCGACAAGGATTTTTCAAACTACTTCCATGA
TATGTGAAAAATATTTACTAATAATTACAGATCTGTGATTAAAAATATTCTAGACTATAAGTATACTAATAATTCCAAAT
CTATGAAAATATTTACCACTCGCAATGAGAGCGCGTGGTTTAGGGTTTAGGGTTTAGGGTTTAGGGTTTAGGGTTTT
AGGGA**TTTAGGGTTTAGGGTTTAGGGTTTAGGGTTTAGGGTTTAGGGTTTAGGGTTTAGGGTTTAGGGTTTAGGG**

Supplementary Figure S2

Color code:

Blue: CCCTAAA and TTTAGGG perfect telomeric repeats.
Light red: CCCTAAA repeats with 1 mismatch and CCCTWAA.
Dark red: TTTAGGG repeats with 1 mismatch.
Orange: CCCTAAA or TTTAGGG repeats with 2 mismatches that are near to perfect repeats or to repeats with 1 mismatch.
Green: DNA sequences conserved between 1L and 5R.
Violet: DNA sequences conserved within the same telomere-subtelomere boundary.

>1L forward

CCCTAAACCCTAAACCCTAAACCCTAAACCCTAAACCCTAAACCCTAAACCCTAAACCCTAAACCCTAAATAAAGCG
CTGTGGGATCAATCATTGCATTTTTCCATAGGATAGATAGGGCCGACAAGATCATCAGGGAAGAAGTCAAATCACAT
CCGAATTCAATTGTTCTTTTCCTAAACCCTAAACCCTAAACACTAAACCCTAAACCCTAAACCCTAAACCCTAAACCT
CTGAATCCTTAATCCCTAAATCCCTAAATCTTTAAATCCTACATCCATGAATCCCTAAATACCTAATTCCCTAAACCC
GAAACCGGTTTCTCTGGTTGAAAATCATTGTGTATATAATGATAATTTTATCGTTTTTATGTAATTGCTTATTGTTGTG
TGTAGATTTTTTAAAAATATCATTTGAGGTCAATACAAATCCTATTTCTTGTGGTTTTCTTTCCTTCACTTAGCTATGG
ATGGTTTATCTTCATTTGTTATATTGGATACAAGCTTTGCTACGATCTACATTTGGGAATGTGAGTCTCTTATTGTAAC
CTTAGGGTTGGTTTATCTCAAGAATCTTATTAATTGTTTGGACTGTTTATGTTTGGACATTTATTGTCATTCTTACTCC
TTTGTGGAAATGTTTGTTCTATCAATTTATCTTTTGTGGGAAAATTATTTAGTTGTAGGGATGAAGTCTTTCTTCGTTG
TTGTTACGCTTGTCATCTCATCTCTCAATGATATGGGATGGTCCTTTAGCATTTATTCTGAAGTTCTTCTGCTTGATG
ATTTTATCCTTAGCCAAAAGGATTGGTGGTTTGAAGACACATCATATCAAAAAAGCTATCGCCTCGACGATGCTCTA
TTTCTATCCTTGTAGCACACATTTTGGCACTCAAAAAAGTATTTTTAGATGTTTGTTTTGCTTCTTTGAAGTAGTTTCT
CTTTGCAAAATTCCTCTTTTTTTAGAGTGATTTGGATGATTCAAGACTTCTCGGTACTGCAAAGTTCTTCCGCCTGAT
TAATTATCCATTTTACCTTTGTCGTAGATATTAGGTAATCTGTAAGTCAACTCATATACAACTCATAATTTAAAATAAAA
TTATGATCGACACACGTTTACACATAAAATCTGTAAATCAACTCATATACCCGTTATTCCCACAATCATATGCTTTCTA
AAAGCAAAAGTATATGTCAACAATTGGTTATAAATTATTAGAAGTTTTCCACTTATGACTTAAGAACTTGTGAAGCAG
AAAGTGGCAACACCCCCCACCTCCCCCCCCCCCCCCCACCCCCCAAATTGAGAAGTCAATTTTATATAATTTAATCA
AATAAATAAGTTTATGGTTAAGAGTTTTTTACTCTCTTTATTTTTCTTTTTCTTTTTGAGACATACTGAAAAAAGTTGTA
ATTATTAATGATAGTTCTGTGATTCCTCCATGAATCACATCTGCTTGATTTTTCTTTCATAAATTTATAAGTAATACATT
CTTATAAAATGGTCAGAGAAACACCAAAGATCCCGAGATTTCTTCTCACTTACTTTTTTTCTATCTATCTAGATTATAT
AAATGAGATGTTGAATTAGAGGAACCTTTGATTCAATGATCATAGAAAAATTAGGTAAAGAGTCAGTGTCGTTATGTT
ATGGAAGATGTGAATGAAGTTTGACTTCTCATTGTATATGAGTAAAATCTTTTCTTACAAGGGAAGTCCCCAATTGGT
CAACATGTGAAAGCACGTGTCATGTTCTTACTTTTGTTTGGGTAATCTTCTAATTACTGTATATGGAAGATGTGAATG
AAGTTTTGGTCCTGAATGTGGCCAAGGTTCCGTCATTTGGAGATACGAAATCAAATCTCCTTTAAGATTTTGTTTTTA
TAATGTGTTCTTCCATCCACATCTATCTCCATATGATATGGACCATATCATACATCATCATTTGTCCAAATGCATGAAT
GAATTTGGAAATAGGTACGAGAATGCCAACAATGACAAGAAGGGATCAAAGACAGTTTTTAAAACAATATTTTACAG
GGTTTTAATCTAATTCTAAGTTTTGGTCACTCACTTTGTTAAAAGAATAATTCAGTGTCTGGACACTAAAATCTTCCAA
AAACCCCATATACATATATGCTATTTCGATACTTATATTTATTTACTCAGCATAAAAAATATTAACCATGTATTCATAGT
AAAATGTTTCATGTGATATCAAACCAGCGACAACAAAAGTATTATTCCCCTCATTATGTTTGACTCCTATTATATTTTT
ATTTTAATTTTTTTCACTATCATCTTTCTTGCAATGAAAGTCCCATATATTGGTCAACATTTCAAACCACTTGTTCTCTT
TTATGTTTTGGTAAGAGCTATCTTCTAAATTTATAATACGCATAAATTCAAAAGTAAAAGAAAATTTTGGTCATGAATG
TTGTTTAAGTCATTTGGAGATACGAAATCAAATCTCCTTGTAGATTTTGTTTTTAGAATGTCGTTCCTTTTTCATCATC
TTAGCTATATCTACAGCTATATATCCTATCTTTAAACCTATATTATTTTTTCCTCTCTTCACCAAAGCCATGTTTTTTAG
TTGTGGCGAAAAATAAGAAATCCATACATCAACATATCGCTTTCGTTACCTTAAATTTTGGCTTGTTATGAAGGCATG
TCATAACGTTTCTAGTCACAACTCACAAGCATACCAACGACCATGATAAATCCAAAAAGTAGAAACAATCTATTATCT
AAACCCCCAAAAGACAAAAGAAAAAAGTAGAAAGAAAAGGTAGGCAGAGATATAATGCTGGTTTTATTTGTTTGTTA
AAAGATATTGCTATTTCTGCCAATATTAAAACTTCACTTAGGAAGACTTGAACCTACCACACGTTAGTGACTAATGAG
AGCCACTAGATAATTGCATGCATCCCACACTAGTACTAATTTTCTAGGGATATTAGAGTTTTCTAATCACCTACTTCC
TACTATGTGTATGTTAT

>3L forward

CCCTAAACCCTAAACCCTAAACCCTAAACCCTAAACCCTAAACCCTAAACCCTAAACCCTAAACCCTAAACCCTAAACCCTTAA
CCCTTAACCCTTAACCCTTAACCATAAACCATAAACCATAAACCCTAAACCCTAAACCCTAAACCATAAAACCATAAA
CCCTTAAACCCTAAAACCATAAAACCATAAACCATAAACCATAAACCCTAAACCCTAAACCCATAAAACCATAAAACC
ATAAATCCCTAAAACCATAAAACCATAAATCCCTAAACCCTAAATCCCTAAACCCTAAATCCATAAATCCCTAAAACC
ATAATCCTAAATCCCTTAATTCCTAAATCCCTAATACTTAGACCCTAATCTTTAGTTCCTAGACCCTAATCTTTAGTTC
CTAGACCCTAAATCCATAATCCTTAATTCCTAAATTCCTAAATCCCTAATACTAAATCTCTAAATCCCTAGCAATTTTC
AAGTTTTGCTTGATTGTTGTAGGATGGTCCTTTCTCTTGTTTCTTCTCTGTGTTGTTGAGATTAGTTTGTTTAGGTTTG
ATAGCGTTGATTTTGGCCTGCGTTTGGTGACTCATATGGTTTGATTGGAGTTTGTTTCTGGGTTTTATGGTTTTGGTT
GAAGCGACATTTTTTTGTGGAATATGGTTTTTGCAAAATATTTTGTTCCGGATGAGTAATATCTACGGTGCTGCTGTG
AGAATTATGCTATTGTTTTGCAGGTCCTGTTCTTAATCTTTCATCGCTTTTGTGCTTATTGTCTCCTTGTCGTTTATGT
TGAGTGGTGTTTGGGCTTTAGTCAGGTTCAGTCCAACCGTTGCTCTTCCTCCCGTTCCTAGTCTCCTCGTCTCAACC
TTTGATTCATATTGGGTTTGTTTCCTTGCTAAGAAGAAGTTTCGTTTTATTTGTTTGGATCGATCGACCTCTAAGTTTA
CAAGGCTTTCTTCTCCCTAATGTTGTTGTTTGTTGTGGTTGTTAGCTGTCTCGGTTCTTCATCATTCTTGGTCTTGAG
ATTGGTGATTTTTTAGAGGTTAGACTTTTCTCCTTGTCTTTGGCCTTCCTGATCTATTTTGATAGGATCGCGTTTTGA
AGGTTTCTAGTTTGACGCAATTGGACGCAGCAGTCGTCAGTCGTCGTATTCGGAAGTTTGCTCGAGGTTTCCTTAA
CTCATTTGTCTTGCGTGCCGAGTTGTGAAGCCTTGGTTCTTGGTTTCTGAGAGATCAGCTCTCATCGTGATAGGAG
GAGGTAGATGTTATTTTTGGGTTGGCCACGGACATCCTTAGTAAATCTCAATCGCGGGTGCTTCAGAAGATTTGGTT
TACTTGTGCTTTTGGTCTTTTGTTTCTTTTAAGATTTAAGTATGTTGCTTCTATCTCAGTTTTGGGTTTCGACCTCTTG
TGTTGGTGGTTTGAAAGCTTTTGTTTTGTATTACTACTGTTTTTAGATTCCCTTCTATAGGGCTTAGCTTCCGGGAAA
ATTTCGTCTTCTGCCGACTTTGACTCCGGAGACTTTCTTGTCGTCCGCCGACTTTAGTTTCTCTTTTTGAGGATTTAT
ATTTCACTTCGGTTCCAATCCAAGATCAATAATATAATTTTAAATGATAAAAAAAAATCAAAAATAATTTCGGGTTTGT
CTTGTAATCTCTCATGTTTAAACCGGGAAACCAAATATTGGAAATAATATCCGGTTTGTCTTGTAATCTCATGTTTAAA
CTGGGTAACCGAATACTGGAAATAATCATCAGAATATATACCTTGCTTTCTTCTTTGTTTTATCTTTGATGATTCATGA
TTACAAAGATGGAACAAGTACGAGGAAACCCAAATCTTGCAAGTTCCAACTAATTGGTTGCTTCAAAGAAAATGTAA
GATGTTGTTTATAAAGAGATGATATGGACAAGGAACAAGATTATTGAATGCAAAGCTTGTTTAGAGGTCTTGTTGGT
ATAAGAGGTCGCTCATGTAAGATCCAGTGTCACTCCAAGACGATGTACTGCAGCATCTTATCTTCTTGTGTTGCGGT
ATATTAAACTTGGGATCTCGAGGTACCGTCGGGTGCTTGTTCGACCTTTAAGCAACAATGGAAAACAACAACACTTA
GAATAGTCGATCTGATAGTCTTCGAAATGGTTCATCAATCTATCAACAAGAAACCAGAAATTCAGGAGTTTCTATTTA
CCTTTTGGGAATGAATGGTCGATCAAAGTATGGCATTGGTTGTGCCTTAGGGACCAACTCTTTCCTCAGCCTTCGTA
TCTCTTCTTCTTCTGCTAGCTGAAGAATCCATACAAGTCTGTCATTTATTCCTACTAGTTTACATAAGAAGCAAAAAG
AGTTCCCTCTAATCTATTTACCTTTTGTTGGCGTTCTCTTTCTGTTTTGTATTGCTCGACTAGGTTTATTTTCTCAGTA
ACCTGATACAATGAGACAATGCTGGATTTTATCATACAAGATCCACAAAAAAGACTCTGCTTGTGTAGATTCAAAGTT
GAGGTCATAACGAGAAACTGAAACATACATAAATCTCAATTATGTGCAAGATTTGTCTCAAATTCTGACCTGGTAATC
AAATTCTGCACGTTCTACTGCTCGAATGTCGGAATGGAGCTTTAAGTCTACTGGTATCGTGATATCTTTCACATGAG
GCTTAACCAGATTCTGAAAACAGAGAGAAACATTAAAATCATCTGCTTATGTTCCATTCTATATTCTATATTTTTGAAG
TGAATTTCTCCGAGAGTCATATATCAAAATCAAACAGTTATATAAATGATCTGGTATGATAGTATGTTAATGAAGAGG
AAAGGGTTCTTCAGCTCACCTCAGGTTCATCAGTTGTCCAGGGAAGACCTTGAGCTATGGGGATTCTCTTTTTTGCT
TTCTCCAACGTCATCTCGTGTAGCTTCTTTGCAAACTCTTCTTCTTTCATTCGTCCCCGTTCCTACGTAGACATTAGA
CCAACAATCATAAATCTATTCTACAACATCTGAAAAATG

>5L forward

CCCTAAACCCTAAACCCTAAACCCTAAACCCTAAACCCTAAACCCTAAACCCTAAACCCTAAACCCTAAACCCTAAACCCTATA
CCATGTACCCTAAACCCTAAACCCTAAACCCTAAACCTATATAGTATACCCTATACCATGTACCCTCAACCTTAAAAC
CCTAAAACCTATACTATAAATCTTTAAAACCTATACTCTAAACCATAGGGTTTGTGAGTTTGCATAAAGTGTCACGTA
TAAGTGTTTCTAACATGTGAGTTTGCATAAGAGTCTCGACTATGTGTTTGTTCAAAAGTGACGTAAGTGTTTAGACTA
GAGCCGGCCGTGAGCACAAGCGGGCCAAGCCCATGCTTGCGGCAGATTATCCTATAATTATGTTTTGCGGCTTTAC
AATTTTGAATTTGTTTTGTTGTTTAGTGGTCGAGTCAGGGATGAGTTTGTTCTCAAACATCTCAAATTTCTAATCTTCC
AAGTGTTAGGTTCACTCTACCTTATTTTTTTTTTTTTTTTTTTATTTTAAATTTCTATTAGAAAGAACTTGAACCTTATTTC
AAGTGAACATTAAAAAGAATTGTAACCCTACCTTATTTCTATTATTATGAATATGTGTAAAGTAAATTTTTTTACAATAA
GCATGTATAAAGTAACATATTTTTGTTCTTTTCTTATAGCCTTGTAAATCATAGGGACGGATCTAGTTTAGAATAAAGT
GTTGCATAAGTTCTTCGACTAACATGTGAGTCCATTTTTGTGAGTTTTGCATAACTTTTGCGCCTTCGACAATTTGAA
TTTTATGCATAAGTGCTTTGACTAATTTATATGTTTTGTCTAAGTGATTCGACATTTCGACTAAGTGATGTGAATTGTG
TCATTCGTCGTCGTTGTCGCATCCGGAAGCGTGTTTGTGGTTCTTGTGTCTTACTTGCCGGGTTATGAAGTCCTTTG
TTTCGAGAGATCTGCTCTCATCATGATAGAAGATGGTAGATGTTATCTTTGGATTGCCGCGGGTTGAATTGTCGCGG
GCATCCTTCTTAAATCTCATTCACATGTTCTTTTGAGGAAGAGTTTTTTTTGTTTTTTATTTATTTATTGTTTCCCTTAA
GATTTATACTTTATTCCTTCTATTTTTTGAGGACTTTTATTTTGTCTGTTGGCTTTAGTTTTCCTTGTTTGGACATTTGT
ATTTTTGCGTCTTGTAACAACTATCCTTGTGAAAATATCAATATAAGAACAACCCTCCTCATTTTAATTCCTTCTTGTC
TACTTAGTTTAATATTTTCCAGCCGCAATGGGCCCATTAGCATCAACACCGGCCTATTTAGACGGCCCGTTATCTCC
TCTTTGCCAATTTTCACCTTCTGCAATGATATTGAAATTGAAGTAAATGCAAACAAAATAGTATGTTCCTGGTAGCAG
ATATTTAAGTTCCATTGATGTATTTCAAAGGGTTCAGAGTTTTATCGTTTTACAAAGAAATGATGAGTGTTCATGACT
GTAAAATCCACCTTCATCTTCCACTTTCAGTTTAACGGCTCCGGCTCTGGATCCGGCTCCACTACTGGTGCTATGCA
ATCCACCTTATATCGAAGAGTCTTTCACAGAGGAAAAGAAACGGCCCAATGCAAAGATCCAAATTAATGAACGCAAT
GGAAAAATGGGTTACTGATTTACTCCGTCTACCCCAAAATGTAAATATCGCCCTACCTTTTTGAAGAAAGTTGAATAT

GAATTATCCCAAACAAGTTTGTAGTTTCCAGCCATTAGCGTAGAGAAGTTGCCCTGCAGGTATAACCACAGTGGATC
CCCAAAACGATTACCTTCATAACAACAACAAAAAATACCCAAAAGCTTCCCCACAAATGCACATGGGCACTGACCTG
GTCAGCTTCATACCGACGGTAAGGTAATATCAGCTGAAATACGAAAAATTCATGATCAACCCTCAGAAATACACTAT
CTGGATGAATATTTGATAGAGTACGAGATTATATATACTATGCGAAAGTAAAGTTTAACCAGAGTTTGGAAATTAATG
AGCAGACTCACAGTCTTTTCCCCTGAAGCATTTATGTACTCCACGCTAAATCCGATATCCTGGTAAAAGACAACAGA
AATATAAACGGAGGACAAACAACAGAAGCTGAGAACCTAACTTAGATCCCGTATCATGTCAAGAAATAACCATTCTT
GTCCTTTTACATAAAAGATTCATCACAGACCAAGATAACGCTGTATACTATGTATGAAAACAAAGATAACCAGAGTCT
GGCAAAAATAGCAAAAGATACAACAATAAAAATCTAATATACGGCAGCTAGAGAGAGTGTGATGTTCTCGACATGCA
ATCCAGAGACCAAAAACTAAGTTTCATGAATTCTTGAACTCTACTCATAAGAGAACAAACTAGACACCCCTGTGAAG
AGGGAAAGAACCCCAATACCATGCTTGGGAAACATATTGCAGGTTTAAAAATCAATACCATGATGGGGAAACATATC
GCGAGTTCAATCATCCGAAAAAGCAATCAAATGATTCAAGGTTACGTATTGGGGTTTAAAAATTATTACCATGCTTAT
CTTGCCTTGCATCAAAGAGAAATCCCAAGCAATATACGAATTTTCAGATTCCACCATCAATGAGATCTAGATAAACAA
AAAGAACATGCATCAATACTTACGCCAGACTCAAACGAAAGTGCTTATCATATGCTTTCGTTTTTCTGCCAGCAGAA
AATCTGAATGCTACATACTACAGTCATCAACATCACTTTTCACGTTTTTGAACATGACAATTTCAGATGGAACAAGTG
TCAAGAGTCTTAGAATGGCAATAAGTTTCAGTTTCATTAATAACCAAAAGCATCTACGGAGAACATATTACCTCATAG
GTTCTCCCAGCTTGAACGATTATCTCCTTGTACTCCTCATTGTCCCTAAGAAGTATAAACACTTAGAGCTAGAAATCG
TGAATTGAAGTGAGAATCTTGTGAATGATGGAAGCTAACC

>1R complementary reverse

CCCTAAACCCTAAACCCTAAACCCTAAACCCTAAACCCTAAACCCTAAACCCTAAACCCTAAACCCTAAACCCT**T**AA
CCCTAAACCCT**T**AACCCTAAACCCT**T**AACCCTAAACCCTAAACCCTAAACCCTAAACCCTAAACCCTAAACCCTAAA
CCCTAATCAAGACATGGTAACTAATTAAGAATAAGAATCAAATAATTTAGTTTGCAACCAAAATAATGTTATTCTAATC
AAGAATCCACCAATATTATATCACACAGTAGTTTAAGAAACACATGAACCAAAACTCATGGTCGTCGACTGAGATTAT
CTGGATGTACCTTTCTGCAGGATTGACTGTGTTTGTAGAAGGGTTAACCGATTTAAGCTGATGTAATGGGATTACTA
CATGTTTAAGATGATGAAGATATAAGTATTACTACCCAGTGTTATTATAGTCTGCACAAGAAATTAGTCACAGAATCT
AACGTTCTTCACACTATTCCTACCATCTATGAGGTCGCTTTCTACAATAGTTATTTATAGCAAAAAGTTCGATTGTCT
CAAATAAAGAAGATTAGCTTATAATAGCTCCATTCAGTTTGACCAGAATTTTTGTACGAGAGAGGGTTATCGCTTGA
GTAAGCAAGTTTAGTAGTGGATACGTAAAGAACTCCCATGACTGGACCAGCTGATGTTGATAAGTAGCACGCAAAT
GAATTCTGCAGCTGCTCCTCTAGAATAGTTTCAAAGGTTTGTCTGAAAATTTTCTCATATCCTCCTTTTGTCAGAACC
TTTGTACTCTGTGCAATTCTTCCCATTGCAGCATCCGCAAAACTCGAAGCAGTTTTCACTGATTGTGATGAATATCAA
GGTTCTGGCAATAGGTGGTGGTAGATGTTATTTTTGGATTGACCGCATGCATCCTTCTTAAATCTCATTCACAGGTG
CTTTAGAGGAAGAGGTTTCTTTGCTTTCTTTAGTTTATTGCATCCCTTATGATTTATGCCTTATTTCCTCTGTTCTTAG
TTTATAGCGTCCTCTTTCGATTCGACTTTTCTATTTGGTATTTATGATGTGTTTAGCTTCCCTTTCATAGGGCTTAGTT
TGCGAGAAAATCACGTCTTCCGCCGGCCTTAGTTTTTGGATATTTTTATTTCGTCCGCCGACTTAAGTTTTCTTTGTT
TAAACATTTGTATTTCTCTTTAGTACCACAACAAGTTTAATTAATCAAATTTCAGATGAGAAAAAAAATGTTATGTGCG
TGTTGCGTCTTGTAACAACTATACTTGTGAAAATATAAGAACAATCCCTCTTCATTTTAATTCCTTCCTGGCTACTTAG
TTTAATATCTTCCAGCCGGGATAACTAAGTCTGAAGAAGTGCAGAAAACTGAGTCTTTTTCAGCGATGTATTCGCCA
ATTCCCCCTTCTTTATTTCAGCAGATACAGCTGGCATATTGTGGTCAAGGTTAGCTTCCATCACTCACAAGATTCTCA
CTTCAGTTCACGATTTCTAGCCCTAAGAGTTTATACTTCTTAGGGATAATGAGGAGTACAAGGAGATAATCGTTCAA
GCTGGGAGAACCTATGAGGTAATATGTTCTCCGTAGCTGCTTTTGTTTATTAATGAAACTGAAACTTATTGCCATTCT
AAGACTCTTGACACTTGTTCTATCTGAAATTGTCATGTTCAAAAACGTGAAAAGTGATGTTAATGACTGTAGTATGTG
GCATTCAGATTTTCTGCTGGCAAACAAAAAGAAAGCATATGATAAGCACTTTCGTTTGAGTCTGGCGTAAGTATTGA
TGCATGTTCTTTTTGTTTATCTAGATCTCATTGATGGTGGAATCTGAAAATTCGTATATTGCTTGGGATTTCTCTTTGA
CGCAAGGCAAGATAACCATGGTAATAATTTTTAAAACCAAATACGTAACCGTGAATCATTTGATTGCTTTTTCGGATG
ATTGAACTTGCGATATGTTTCCCCATCATGGTATTGATTTTAAAACCTGCAATATGTTTCCCAAGCATGGTATTGGGT
TTCTTTCCCTCTTCACAGGGGTGTCTAGTTTGTTCTCTTATGAGGTAGAGTTCAAGAATTCATGAAACTTAGTTTTTG
GTCTCTGGAATGCACCAACTAAGCTGCTTTGGTCTGACGCTGAGGTCATTTTCAACTGGGTAGAGACTAGAGACTG
TGAAGGGTAATGTATCTCGGGCTTTCTTCGAAACCATATGACCAAATGGTCCATGTCCATCTAATACACCACAAAAC
ACTGATCGTCTCTCGAACAAAAAGTTCTGGAAATTGAAATGGTTTTCACAAGATAGAAATTTGCAGAGATAGAATATA
AGAGCCAGTTTGACGCCCGGACGAGAGTCTTTCTCATTCAAAACCAACCAATTTCGCCGAGGCAGAAAACGTTTTC
GATTTTATTTACCAAGAATCGGATTACATCTATCATCAAACCAAACCCTAGAAAGTTAATCGGATTGCATCTATCCGT
ATCAAACCAAACCCTAGAAAAAAGTTAATCGGATTACATTTCTCCGTAACAAACTAAAAGCCAAGGAAAAAAAAAAAA
AATCGATCCTAAACGTAGAATGCCCAGCTCTACTCAGCAGCAGTCGTCACGACGTGGCCTGCCCTGAAGCCGCCG
TCGGGCTATCTCTGGAAGCGGACCGAGTAGTTCCATGAGTGTTGCTTCTCTTCTTTCTTCAAGATTCCACGGTTTCA
TATAAACATTCGTCCTCAAAGCCACTCGCGCTTCACGAAACGCCTTTCGTATTAGCCAATAGACTTCATCACCGGAG
CTCGCTTTGAGTCTTTGTAGATCCTTTTTGATTTGTTCCCCCTCTATGCCCCCATTGAACAAAGCACTGACTTGGTGT
TTCCAAGCATTGCTTATTGAAATTGCTCGGTTTAAAGTTTCTTGCTCCGTTTCCATCGCCGTCACTATGTGGCGTGA
ACATGCTTCTGTAATTTCATTAAGGTTTAGTCGTCCCATTCTTCTTTCAGG

>2R complementary reverse

CCCTAAACCCTAAACCCTAAACCCTAAACCCTAAACCCTAAACCCTAAACCCTAAACCCTAAACCCTAAACCCTAGA
CCCTAAACCCTAAACCCTAAACCCTAAACCCTAGACCCTAAACCCTAAACCCTAAACCCTAGACCCTAAACCCTAGA
CCCTAAACCCTAAACCCTAAACCCTAGACCCTAGACCCTAAACCCTAAACCCTAGACCCTAAACCCTAAACCCTAAA
CCCTAGACCCTAAACCCTAAACCCTAAACCCTAAACCCTATACCCTAAACCCTAAACCCTAAACCCTAAACCCTAAA
CCCTAAACCCTAAACCCGAAACCCTAAACCCTAAACCCTAAACCCTAAACCCTAAACCCTAGACCCTAAACCCTAAA

CCCTATACCCTATACCCTATACCCTATACCCTATACCCTATACCCTATACCCTATACCCTAAAACCCTATACCCTATA
CCCTAAGTCCCTAAAACCCTAAATCCCTAAAACCCTAAGTCCCTAAAACCCTCAAACCCCAAACCCTAAAACCCTAA
AACCCCAAACCCTAAGTCCCTAAAACCCTCAGACCCCAAACCCACAGACCCTCAAACCCTAAAACCCTAAACTCAC
AGACCCTCAAACCCTAAACTCATACAAAATTTTATGGTTAGTGTATATCAACATATATGTTTGTTTGAGTGTCGCATTT
TGAAAATTGAAGTTATAAATAGATTTACTTGTGTGAATTGACATTTCCCATTAATAAACCCTTTTAAGTTGGACAATTT
ACTTAGCAAATATTTTAGGAGTCAAAGAAAGAACATTCAATGCGAGTGGAAACATTGATGTCGAAGTTCCCACAGAT
GTTTCCACCAAAGCAAACCACTTGAGCTGTTCAATAAAAGCCTTGCAACACTTGCCATATAATCGGAAAATAGATAA
CAATAAAACGAACCTCTGAGATATTCGACAAGGTATTTCTTAACTTGAGTAAGGTCTGAAACGATTTAACTACTCTAC
CTTTGATTTTTATTTTTGGTTTTGAGAAGATGATTACAGTTGTGATTTTGGTGGAGACAAAAATGTCCATCTCTGTATG
TTGCTAGCCATCTTTTGTCCAACAGGCCACCTAACACTTTGGAAAAACCCGTTCAATCTTAAATGGGCCATGTTGAG
CCACTAATGGGCCTCAATACTATGAGATTTTAAGTGTCAAGTGTTAACCTACGCAGCAGCTAGGGCGCACGTTAAC
AAGGACCATTCAACAATAATATCATCAGTTTTTTTTTTCTAGGTTCGTGTGTGAATCCCCTTCTTCCGCATCAAAAAA
AAAACAATCTACAGACAGACAATCGTCTTGTCCTGGATTAGGGTTTCATTTTCTCCACTGTCTAGCAAACCCACTTCT
TCCGATTGGGGCGGGGATTCAATGCCTGCGAGTTCATCTGGGGAACCTGTCACCGAAAATCCTATACACTCCCAAT
CTCGCCTCCGGTTCTTGCTTTACTTGGAGTGTTAGAATTCGCTTCTTTACTTGATGGCTCCGAGCCGCAGGAAAGG
CGGTGGTAGGGCTGCCGCCGTAGCTGCTGCCTGCCGAAAGCGGAAGGTGGGCGACCTTGTGCTTGCCAAAGTCA
AAGGTTTTCCTGCTTGGCCTGCCGTGGTATGTATTTATTATTCTCTTTTGCGTTTGCTTCTTTATGGATGGCCTATAA
GTTTCTCCACATGTTACCAATCCTATTTATCTATCTAGGGTTTATTTATTTGGACATGTACCTGCTTCTCCTAGGATTG
AAAATCATGTTAAGTCGATTCTTTCTTGTCCGATTGTAGACTGAGTTTTAAGTTCTTCTGCCTAATCGATATGAGGTT
TTTCTCAAAGGAGCTGAAATCCATTAATTCTTGTTGTTAGTAGAGACGGGAACACAACTAGTTTAGTTTGTCCTTGTT
TCAGCCGCATCTGATATCCCATAATATTTCTCCTCTCTCAGCGCTCTCCTTTTTAGCTTCTATTTTTTCGATCTTCTGG
TCTTTCTGCTTATTGTTGGATGCGATTTCAGGTTAGTGAGCCTGAAAAGTGGGATGCATCACCCGATTCGAAGAAAG
TGTTTGTTCACTTTTTTGGAACACAACAGATGTGAGTTCTTATCCCTTTAACCTTTTCCCTCTTCAACCCAGCCTGGT
CTACACATGTGGGTTCATTTGAGCTGTTAATGTTAATTTAGTTGCAGCGTAATAAATTTAAATATATAGATTTAAGTGT
GTAATTTTCACCCTGGGGGTTCCCTGTTGAATCACTCACATCGTCCTTATGTGTAGTGCCTAATGTCTCTGTATCTA
GATACTTGGGAACATTTGCTTCTTGTTGGACATTTAGTTTATGCTGTTGGGTTTAACATAGTTATCTGATTTGGTTTT
GGTTGCATTCTTTCCCTGCAAAGTATCTTTTTCATGTTTGTTTATAAGAGTACAGACACTAGTAGGACGTTTTTTAAG
TTAAAATATTTTATAGTTAGATTGTGCTTGAAGCTTTTGGATTGGTTTATTTTTGTTCTCCTTTGAAAATTGTTCTCAAT
CTGTTAGGTTGAATAGAGAATAGTGATGAATTCATTGTAGTCTAGAGAGCATTCTGTGAACAGCAGCTTTGCAAGAG
CCAAATTTTCTCAGTGCTCGCTTTCTGCTTTGCCCTCATAAATATCAGTGAGAATTTTGTTTGTATGTAATCATATGG
AGTTGGTGACATTTTCTTGTTTTTTTTTTTTTTTGTAATTCATATGCAGAGCTTTCTGCAATCCTGGCGATGTTGAAGCA
TTTACAGAGGAGAGGAAGCAATCACTTTTGACAAGACGCCATGCGAAAGGGTCAGATTTTGTTCGTGCTGTGAAGG
AGATTATAGAGAGTTATGAGAAGCTTAAGCAGCAAGAACGAGCTAGTGATCCCAAATCTGCTGAAGAAGGCACTCT
TGGGAGTGCTGAGAATACAACACTGATGCCTCAGGTCATTGAAATTCCAACTGCG

>3R complementary reverse

CCCTAAACCCTAAACCCTAAACCCTAAACCCTAAACCCTAAACCCTAAACCCTAAACCCTAAACCCTAAACCCTATTA
TTACTTATTGTAATCCTAAGTTTGTAATCTCACCCAAAATCTTCTCTTTACCAACAACATAAGTAGCTACAAGAAACAA
AACCTTCTCCTCACTAATTTTATCTATCACATAACCACATAATCAACAGACTCTTCTTGACTCGGGAAAACAAACTTA
GGATTACAATAAGTAGTATCCAGAAAAACACCATCACAACCAACAAAACCATTAAGAAACGAATCAAATCTCAACTGT
ACCGCTCCTGGACAATGATTAGCTTCAATCAATACAACTTCAAAGCCATCGATCTTAACCATTTGGTTCATATGTAAA
GCGAAGACGAAATGTGAAGGAACTTGAAGGATTTCTTCAACGAGACGAGCGGTTTTGTGTGAGCAGTAGATAATTC
CTTTAGACCAAGTAGAGGAGAGACCGGAGTAGTGATCAGAGTGGAAATGTGAGAGGAAGAAAGCGACGGAGAAGG
ATTGGTGTGGGAGGCGGAAGAGATCGACGATGAAGTTAGTTTTAGGGATTCGTTTTGAATTAGGGATTGAAGGAGA
TGAAGATGTTGGTTTCGGTGGGGGGAATTGTGGGGAGATAGAGGAAATTGCTGATGAGTAGAGTTTCGTAGTGTTG
AGATTTAGGGTTTCTGTGTTGTCGGAGTTTGGGAAGTTGCCGGAGATTGTGGCTCCGGCGGAATCGGAGTCCATG
ATGAGGAAGATGAAAATTGGCGCCAAAATGTGACGGTTTAGGGATTGGATAGGTTTGCCTTCGTCTGGTTTTTGGA
GAAAACTGAAGCAATGAAGCGTCCAGGCTATAAGTTTTTGTAACCGTTTAGTTAAACCATTCTAATGTATTTAGTTCA
TTTACGAACTATTCTAAACTTTGGTTTATTTTATGGTTAAATTTTTTAAGAAGACTCAAATTTTTAAGAGTGTTGGATG
GTATAATTTAAGTTTACAAGAACCGTCAAAATCACCTCCCTCAAATTACAGTGTTCACTTTGAGATGACTCAAGATGT
GCTAAAAAATTAATCACTTTCAAGACTACATAAGGAATAGGTTGACGGTAAATGGGAAAACATCTTTTAAAATATGCT
TTTACAATGACCATTTTATTTTATCACATCATTATACATAATATAATTACCATGTTCGGCACGTGGTCACAGCGGTCA
CTTTCTGCGACTCCAAAATAGACGTCGACGGAGTTTAAATTATTAAAAAGAGTCGCCACTTTTGTTGAAAGGTGACG
TCGTTGCTGTTAAAGGAGTCGCCGCAACAACCGCCGTTTTACTTTCTGCTTGGCTCCCGTCACTGTCATTTTACATA
AACCAAGGTACCCAAATGTAACAGAACAATGACATGTCGGTGAAGAATGGACTCGTTTAATATCAGACTTCAAATTT
AGGCCCATCATTACATGAAAAATAGATAAACAGTGCGTTTTTAAATCCATATAACTCCTATAAATAAATTTTTAAACAT
TATGACATCTAAGACCAAAACAAGATAGTGGGGGATTGAATTGATCTTTGTAATTTTTTTTAGATAATATTAATGGATA
GCAATTTTACACTATAATTAAGAAACTAACGTTTGTATCAATTTTCTTTGCAAACTAACTCTTTTTCATGTGGGGGTAG
ATGTAAGATAGATATCAAATTGTTTTTTTAGATCATAGTAAGTACAAAACAATAGATCATAGAATTGTAGTGATAACTA
AAATTACAATTCAAAAAGTGGTGATGATGGTAAATATGGAGGGAGAGGTCACAGTGAAAGATAAAAAAATGAAACAA
GTAGCAAAGTTACAAATTTGGATGAAGGTGGTGTTGTCAAGATTTATGAATGCAATACCGGAAGTTACATATGAGAT
ATATAAAGAAGACACATGTGCTAGTAATTTTAATAAATAACATGTACCAAATAAAAATGACACGATATCTGAAAGAAA
AACATTGAAATAGTTTAAAATTTGTGAAAACTACAATGTTTTTGAAAAATATAGCTTGTCAAAAAGAATAAAATAAAAT
AATTATTAGGTTGTTTAGACGCAAGTAACAAGAGTACGTACTTTGGATGAGGAATAAACAGCTATCAGAGATTGAAT
ATACGCAATTTACTTAAAAAGAAATAAACAGCTATCAGAGATTGCATATACGCAATGTAACAATTAGCACTAAAGGAG
TTGAGTTGTCGGGAAAGCAACTAAGCAGATGAATAAGACGATGATATAGGTGAAAGTGAAACGGTAGTTCCAGTTG
GTAACAGAAGGACAGGTAAGCAATCCAAAGGATTTTGATAAATATTATAGCCCCACGTTACTACATCTGTATAGCTC

ATGTGCGTTGATTTGCAGGAAGTAGTGATATTTGTTAAAAATACAAAATTAATAAAATGATCCCCTCTTAACGCAAGT
CATAATGCGTTGGGGTCTAAGACATAATCTTCCAACTCAAATCAAATCGTTACAATGAATGGGAAACACCCCAGGAA
GAAGAAAAAAAGATGTCATCACAACATCTTTGCCTTCTCCCTTATTGTGCTAAGAGAGACTCTGTTCATATGTGCATG
TTCTCAAGGAAGTCGGACAACCTATAAAGACGAATAACCATCCAGAGGTAAGCTCAAATCAATTGAAACAAGAGACT
AGTGGTACAAGTTGAAGGCACCAATACCTTATATCCTTTGCTATCACAGTCTGCCCATCTTCTGCATCCAGATGCCG
CAAAACCACTTCCTCCAAACGACCATCATGATACGCATGCTGCAATCACACCCACAACCCCCCTTTTGAGTTTTAGA
GGTGCATCCATATCAACACCAACAAATATACATTTCAAGAGGAAAATAGTCAAATTATGCTTCACTCAAAAGACAAAA
AACGACTACGAACTCCCAACACCATAATATAGCCATATAACCTAAGACTCTCACTT

>4R complementary reverse

CCCTWAACCCTWAACCCTWAACCCTWAACCCTWAACCCTWAACCCTWAACCCTWAACCCTWAACCCTWAACCCTWAACCC
TAAACCCTAAACCCTAAACCCTTAACCCTTAACCCTAAACCCTTAACCCTTAACCCCTAACCCTTAACCCCTAACCCT
TAACCCTTAACCCTTAACCCTTAACCTTTAAACCTAAACCCCTAAATCTCTAAATCCCTATAACCTTATCCCTCACCCT
AAACCCTATTTTTCACTCTATCTCTCCTACTGTCTCTCTCTTTCTATGATATCTATCTATGTTTGCACTTTTTCTTCTTG
CAAATTCTATTTAAAAGGGAATGAAAGAAGAGTTATGTTTGCTAATCACCTAGTGCGATGGAGATTGTAAGAGTAAC
AACCAAATCTTGTTTTACAACCATACACTATCATCTCAAAACAGCATTAAATCCTAAAATCCAATTATATTTTTTATTAG
CAATAATATGATTAGGAGAAAATTTAGAAAAGAAATGCTATCGGTGTATTAAGACATGGTAACTAATTAAGAATAAGA
ATCAAATAATTTAGTTTGCAACCAAAATAATGCTATTCTGATCAAGAATCCACCAGTATTATATCACACAGACTGAGG
CACCGGCTTGCAATGTATCTTGTAGAGATGTAACAGCGCCTTCGTAGTTTAAGAAACCCATGAACCAAAACTCATGG
TCGTCGACCGAGATTACCTGGATGTACTTTTCTGCAGGATTAACTGTGCTTATCGAAGGGTTAACCGATTTAAGCTG
ATGTAATGGGATTACTACCTGTTTAAGATGATGAAGATATGTATTACTACCTAGTGTTATCATAGTTTGCACAAGAAA
TTACTCACAGAATCTAACGTTCTTCACTTTATTCCTATTATCTATGAGGTCGCTTTCTACAATAGTTATTTATAGCAAA
AATTTCGATTGTCTCAAAATAAAAAGGATTACCTTATAATAGCTCCATTCAGTTTGACCAGAATTTTTGTACGAGAGA
GGTATCGCTACAGTAAGCAAGTTTAGCAGTGGATACGTAAAGAACTCCCATGACTGGACCAGCTGATGTTGATAAG
TAGCACGCAAATGAATTTTGCAGTTGCTCCTCTGGAACAGTTTCAAAGGTTTGCCTGAAAATTTTCTCATATCCTCCT
TCTGCCAGAACCTTTGTACTTTGTGCAATTCTTCCCATTGCAGCACGCAAAGCTCGGAGCAGTTTTCACTAATTATG
ATGAATATCAAGATTTAATAGTTAGTTACCATAAATAATCCCATCAAATCACAGAATAAACAGAAAAAACAAAAGTTAA
TATTCGCTTTGAAACTCCATTGCTCAAACCTATAATATCTAACTCATCAAGAAGTGATATACCATGTAGCATGACAAT
CACCCAAATATCAACAATCTCAGCTCATAGTATATTTTCTCAGGGTTAACGATGCAACAATACAAGATCGGAAACTCA
CGGTGTTGCCACGTGTTCCCGGCAAGGCTCTCTGCCTTCATTGCAGCTTCTCCAACCCTCCTTCCCCATCTCCCAA
GCACACCTTTCACAGATTCCATCGTTTCTGATCCAATTTCAAAGAGATTTTAACGGTTACTTACACATCTCATAGCAT
AAACCAAAAAGATACTGACACAGTTTTAAACACAGTACCTTTCAACGAGGCATCCGAAGTTTCAGTAGGAGCACGAG
CGACGTACGGATTAGATCTCGCGGCAGAAGATGAGGATAAGGCCTTCTCATCCGGTGCGGGAGATTCAGAAACCA
ATTCGGGATTCCAATGGAGATTTTCTCGATCCGGACGATGTTCGATTCGGTGCTCGATCGCCTTTTGAATCGATTTC
GACGTGTAGCGTAGTAGATTCATCAGATAAAGCAATCACAGAACTCGCATCCTCGGCTCCTTTTGCCGCCACTTGA
TCCACATGTTTCATATCACTACTATGCGGTGGCTTCAAATTCTGATCCTCCGATTTCAGTGTCGTCTTTCGTCAGTTG
TTCCATTCTCCGATTGCGCAAGTATCGTATCGTTTTTTTATTGTCGTTGTATTTGGGATGTGGGGTAGAGTTTTTATT
TTATTTTATTTTTTTCCGAGAAACGCTGAAGAGAAAAAATAGTAAATGGAGAAGACGAGAGAAAGCAAAAATTAAAAA
AAAGATCTCTGATTTTATTTTATTATGTAGATTTTGGGCTATAAAACTTTTTTAAAACAAAAAAGTTATGTTATTATTGG
ATTCTGAATGTGAAAGAAAAAAAAATGGTGGTTGTGAATGTTTTTAGGATGACTTATAGAATTAAAATCATTGGCTAAT
TTTCTAAAAACTTAGAAAGTAAAGTTATTTTTTAATATCTTATTAAAAAAACTTGAAAAGCAGATCATATTTAAAACATA
TCAACAATGGCCATTTGAATCATACATATAATTTAAAACGTTACATTTTTTGTTTGTGGGAATCAAAACTCATAATTAC
AGCAATGGTATTTAATTAGGATGACTTCTCTTTTTTGTTGCTATTAATGTTAATATCACTAAAATGAAGAGTTAAGGTA
TTACGTAACAAAAAATGCAATCTGCAAAAAAAAAAAAAAAAAAAATTATAATATAATATGGCTAAGAGTGTGATTTAACG
GATCTAGAGAAGCATTAGATTTCTCCAACTCTTCCTATGACGCCGAAACGGAGATGGTATTGCGTACCTTATGATCG
ATAGACTTGAAGATGAGCAGAGGAGGGATGGACTGCAGATTATGGAGTACATTGTTCCTCTGTTCAAATATGGTAG
ACCGGAGCTTGGGCAGCCACCTAGGATGACTTCTCTAGAATCGGTTTTGGTTTTGATTTTAGAATTTCGGTTCAAAT
TTTGGTTTGGTTTGGTTTTGGTTAAGGATGAAGGTTCTGTTGTAGTACCCAATATTAGTATATGAAAACTTATTCATAA
TAATATAAATGTCATAATCATTCACAGATTAAGATAAGTTGCTTG

>5R complementary reverse

CCCTAAACCCTAAACCCTAAACCCTAAACCCTAAACCCTAAACCCTAAACCCTAAACCCTAAACCCTAAACCCTAAATCCCTAA
AACCCTAAACCCTAAACCCTAAACCCTAAACCCTAAACCACGCGCTCTCATTGCGAGTGGTAAATATTTTCATAGAT
TTGGAATTATTAGTATACTTATAGTCTAGAATATTTTTAATCACAGATCTGTAATTATTAGTAAATATTTTTCACATATC
ATGGAAGTAGTTTGAAAAATCCTTGTCGAGTTGCACATCATGAGTTCCATATCACATCTAGTTTCATATATATCACTT
GTAGTTTCTGTTTCACACGTCCTGAATTAAATGACGAGAGAGATATAAGAAATGATAGTTAATTTAAATATGGATGGT
AAGTAAAGACATTAACAAAAAATTTAGATTAAGTCTCCTGGTCAAGGAAAAAAGAGAAAGGAAAATAGTAACAAATTT
AAGATATATAACATGATGTTAAATTTTTTTAACTTGAAGACGACAACCGAGGCGATCGAAAGCTTGACCAGTCTCAC
GGATCTAAAAACCCTAAATCCTAAACCATATACCTATACCATACCCTAAATCCCTATACCATATACCCTATACCCTAT

ACCCTATACCCTAAATCCCTAAACCCTAAACTCTAAACCGTAGGGTTTATGAGTTTGCATAAAGTGTCTTGTATAAGT
GTTTCTAACATGTGAGTTTGCATAAGAGTCTCAACTATGTGTTTGTTCAAACGTGACGTAAGTGTTTAGACCAGAGC
CGGCCGTGAGCACAAGCGGGTCAAGCCCATGCTTGCGGCCGATTATCATATAATTATGTTTTGCTGCTTCATAATTT
TGAAATTGTTTTGTGGTTAAGTGGTCGGGTCGGAGATGAGTTAGTTCTCATACATCTCAAATTCAAATCTTCCAAAAT
GTTAGGTTCACTCTACTTTATTTTATTTTTATTTTTTAAGTTTAAATTTCCATTAGAAATAACTTGAACTTTATTTCAAAT
GAACATTAGAAAGAATTGTCACTCTACCTTATTTCTATTATTATGAATACGTGTAAAGTAATTCTTTTTTACAATAAACA
TGTATAAAGTGACCTACTTTTGTTCTTTTCTTATAGCCTTGTAAATCATAGGGACGGCTCTAGTTTAGAATAAAGTGT
TGCATAAGTGCTTCGACTAACATGTGAGTCCATTTTTGTGAGTTTGCATAAGTGTTGCGCGTTCGACAATTTGAATTT
GATGCATAAGTGCTTTGACTAATTTATGAGTTTTGTCTAAGTGATTCGACATTTCGACTAAACCCTAAAACTGGCGTG
TGAGTTTTGCCTAAATTCTTCGACTAGAGTGTGTGTTTTGCATGCTTCGACTAGAGTTTTGCCTAAATGTTTCGATTT
AGGTTTCATAATTATGGTTGTGGTATGATGCATTTTAGCTATATAGCTTTTACGTTTTGCATTTGCACTGGGGTTGGT
TAGCTATTTTTATCATTTGTCGCTTGACGCTATGTTTGCATCCTATACATCCATCTACCTAACACACACAAACAAAATA
TAACCCTACACATACATCTATTGCATCCATCCTCTCACCATATACATACACATCTAACTCACACATAATAAAAACCCT
AATCATACATCTATTACATCATTAATATTATCACCACACAGACACATAATAACCCTACACATACACATCTATCATTACG
TACATCATCCACTCTCTGATACCAGTTGGTATCAGATTCATCTCGTTCCTCGTGGTTTGATTCTCTTTCACCGATGTC
TTTAAAAAAACATGAGATGGGAGACCAATTATAAGTTTCGTATTCCCAACCTCCATGGTGGAATCTCTAGTGATTCGT
TACTAGATTGGCTTGTGGAGATTGAAGAAATTTGGGAGTTTCAAAACGTTTCGGATGATCTATTTGTTTCGCTTGTAG
TTAAGTAATTTCGAGGGCAAGCCGCATCATGGTGGCAACAAGTACAGAAAACAAACAAGGCAAGAATCCTATTAAAT
CATGGGATAAACTTAAGGAGAAGCTATGCAAAACTTTTTCACCTCCTCATTATACGAGTTTGGTGTCTAATCGTTTGA
AGAATTAAAACAAAGAGCCCGATTTGGGGATGATTTCACGAAGAATTGTCACGTTCAAGTCAACAACTTCGTGGAGA
AGCCTGCTTATGATCAGATGGTCACGTTCAAGACAAGGACTTTGTTGCTAAGTCCGTCCATGGTATTAATGAGGATG
AGTTATCTATCTACATAAAGCATTTTCTTGAGGTAGCCGTCAATGATATATATGGTGATAAATTATCTACTATAGAACC
ACTCTTCGATGTCAAGGAAGTCAGTGACAGTGTTGTCGATGTTGAAGGCGACGATGGTTTAATAAACCCCAACGAC
ACGGACGATGACGCTGATGTTGCAGATCCTGATGAACATGATCAAATTCAAGAGGAAGATAGTGACTTTGATGCCA
AAAAGCTTGATATTAATTTATCTACATCGGAGTCCCTCTCCAATGGGGATTTAATATATGATGTCAAAGTTAACATGC
CGATTGATAATATCAAACCCATAGATTTCGTTTCCGATGTCTTTGCTCTGGTGTGATGAAGACGTCAAAGACGCATA
TTTCATCGGGAATCAAGAAGTGGAATCACATATTAACCATGAACTAGTCATCAACAATCTTGACGAGAAGGAGGAAG
TTTCATTTATGTACAAGGAAAAATACAATTATTCTATTTCTCTTGCAAATCGTCAATGGAATGTCAATGACGAACCTCC
AGATCGTGGTCGAAGTGAGATTTTGGTTTTTGACGGTAAGGTTGATCTCTGCAACAAGCTCTATGGTACTCTGGTTT
CTTATATCTCTAATGAGAAAACTCATTGTTATGATGATTCTTACGAAGAAGACAAAGAGATAATTGAAGTTAAATTGC
TTGAGAAATCAAAAATTCTTGTACCTTTTCGTCATTGGAG

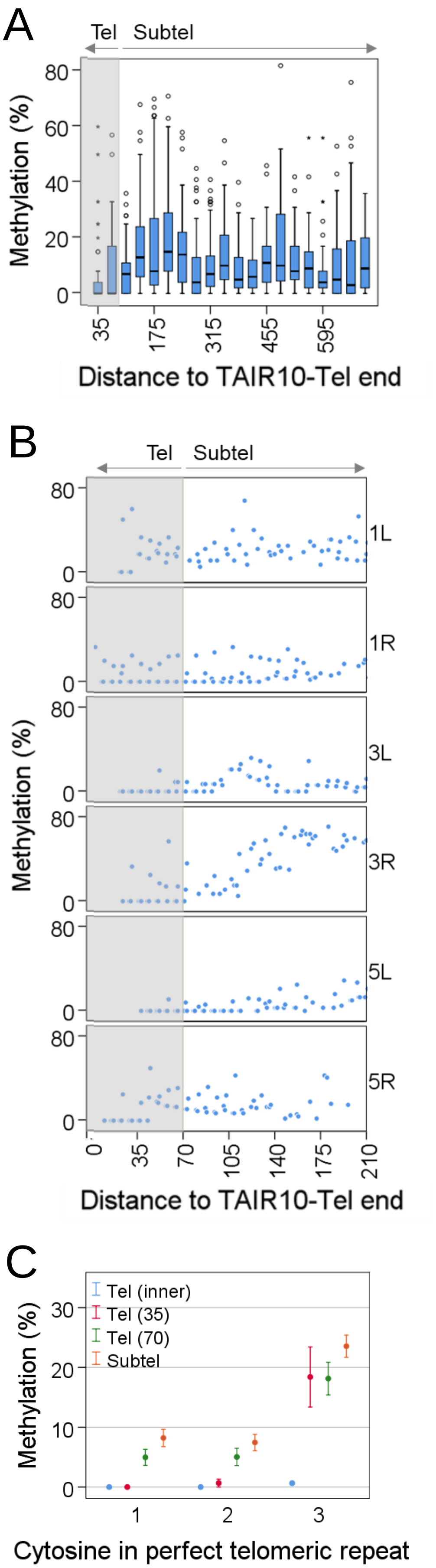

Supplementary Figure S3

A

Experiment (Millions of reads)	Reads length (bp)	*A	*B
SRR771524 (106)	100	1.28	15.2
SRR5494752 (29)	101	0.45	9.0
SRR5494753 (32)	101	0.69	16.5
SRR5494754 (40)	101	0.83	16.3
SRR3384734 (34)	151	0.52	9.4

B

-SRR771524 (total=3167)
[2685, 316, 66, 31, 12, 13, 4, 1, 2, 4, 0, 3, 1, 1, 3, 0, 1, 0, 0, 0, 0, 1, 2, 0, 1, 0, 0, 0, 0, 0, 0, 0, 0, 0, 2, 0, 0, 0, 0, 0, 0, 1, 4, 2, 11]

-SRR5494752 (total=1059)
[964, 71, 10, 4, 1, 0, 1, 0, 0, 0, 2, 1, 2, 2, 0, 0, 0, 0, 1, 0, 0, 0, 0, 0, 0, 0, 0, 0, 0, 0, 0, 0, 0, 0, 0, 0, 0, 0, 0, 0, 0, 0, 0, 0, 0]

-SRR5494753 (total=916)
[765, 110, 17, 12, 2, 3, 1, 0, 0, 2, 0, 0, 1, 2, 1, 0, 0, 0, 0, 0, 0, 0, 0, 0, 0, 0, 0, 0, 0, 0, 0, 0, 0, 0, 0, 0, 0, 0, 0, 0, 0, 0, 0, 0, 0]

-SRR5494754 (total=729)
[610, 70, 22, 11, 6, 3, 1, 2, 0, 0, 1, 0, 0, 1, 0, 1, 0, 0, 0, 0, 1, 0, 0, 0, 0, 0, 0, 0, 0, 0, 0, 0, 0, 0, 0, 0, 0, 0, 0, 0, 0, 0, 0, 0, 0]

-SRR3384734 (total=53)
[48, 1, 0, 2, 0, 0, 1, 0, 1, 0, 0, 0, 0, 0, 0, 0, 0, 0, 0, 0, 0, 0, 0, 0, 0, 0, 0, 0, 0, 0, 0, 0, 0, 0, 0, 0, 0, 0, 0, 0, 0, 0, 0, 0, 0, 0, 0, 0, 0, 0, 0, 0, 0, 0, 0, 0, 0, 0, 0, 0, 0, 0, 0, 0, 0]

Supplementary Figure S4

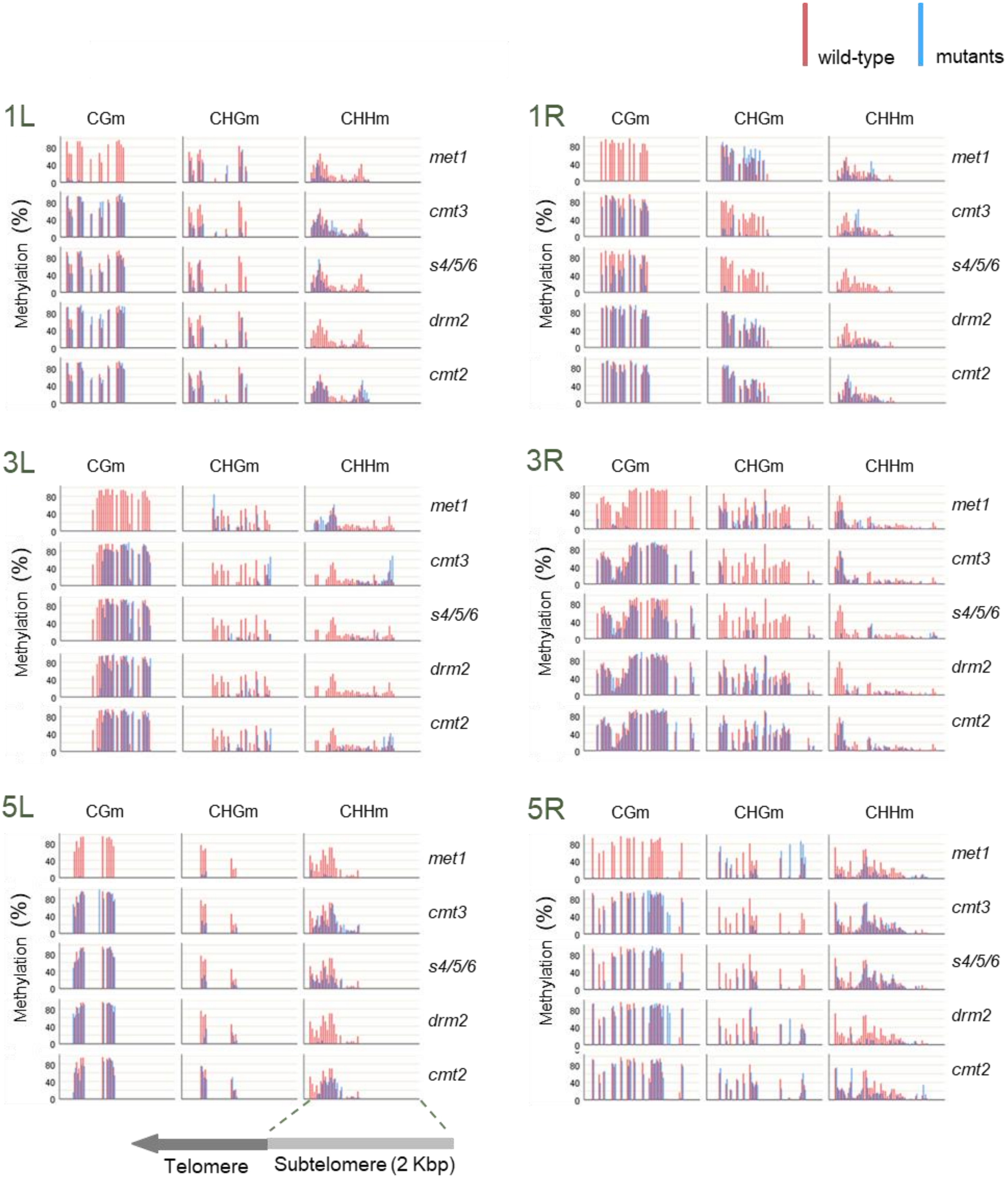

Supplementary Figure S5

A

	PC1	PC2	PC3
nºCG	-0,17	0,16	-0,16
nºCHG	-0,03	-0,10	0,08
nºCHH	0,13	-0,22	0,02
nºCCG	0,12	0,03	-0,41
nº(CAG+CTG)	-0,12	-0,12	0,37
WT CGm	0,02	0,85	-0,02
met1 CGm	0,65	-0,33	0,03
cmt3 CGm	0,08	0,93	0,02
suvh4/5/6 CGm	0,03	0,70	0,03
drm2 CGm	-0,10	0,84	0,06
cmt2 CGm	0,03	0,94	0,06
WT CHGm	0,32	0,31	0,72
met1 CHGm	0,05	-0,15	0,67
cmt3 CHGm	0,88	0,17	-0,03
suvh4/5/6 CHGm	0,46	0,25	0,19
drm2 CHGm	-0,02	0,12	0,84
cmt2 CHGm	0,33	0,43	0,61
WT CHHm	0,85	0,03	0,20
met1 CHHm	0,76	-0,20	0,19
cmt3 CHHm	0,89	0,09	-0,05
suvh4/5/6 CHHm	0,54	0,05	0,09
drm2 CHHm	0,05	-0,11	0,62
cmt2 CHHm	0,88	0,12	0,13

B

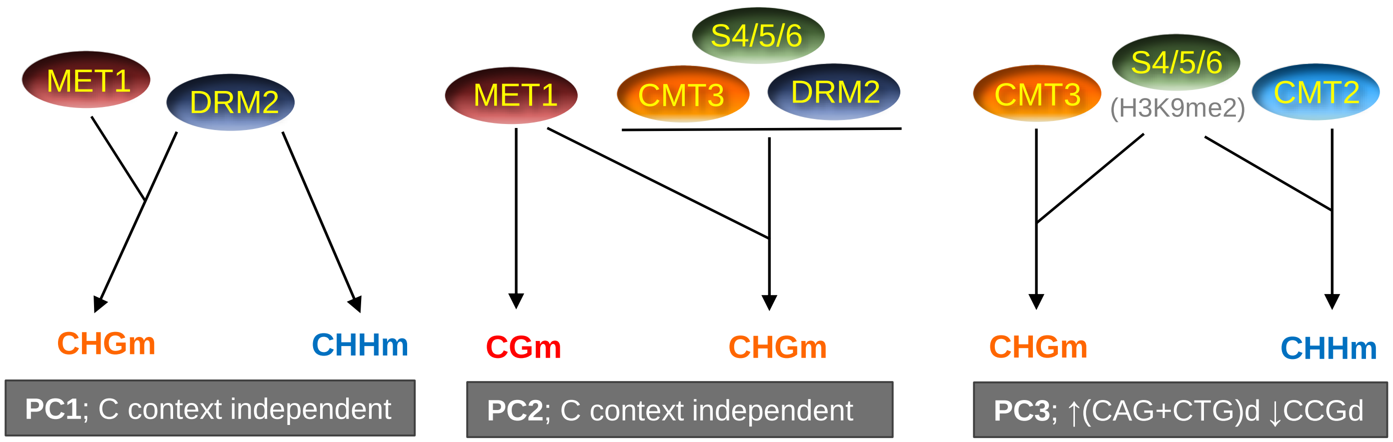

Supplementary Figure S6

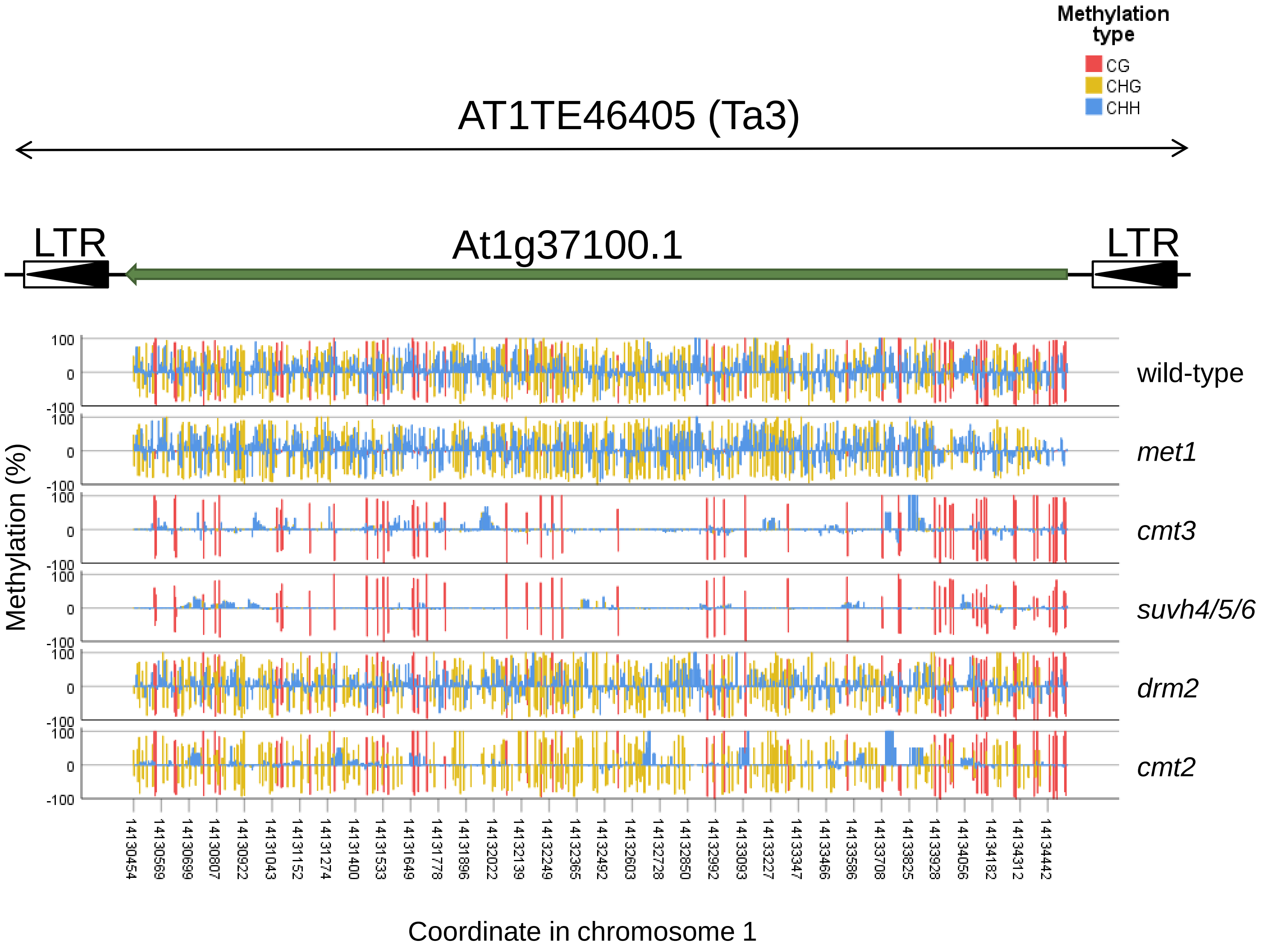

Supplementary Figure S7

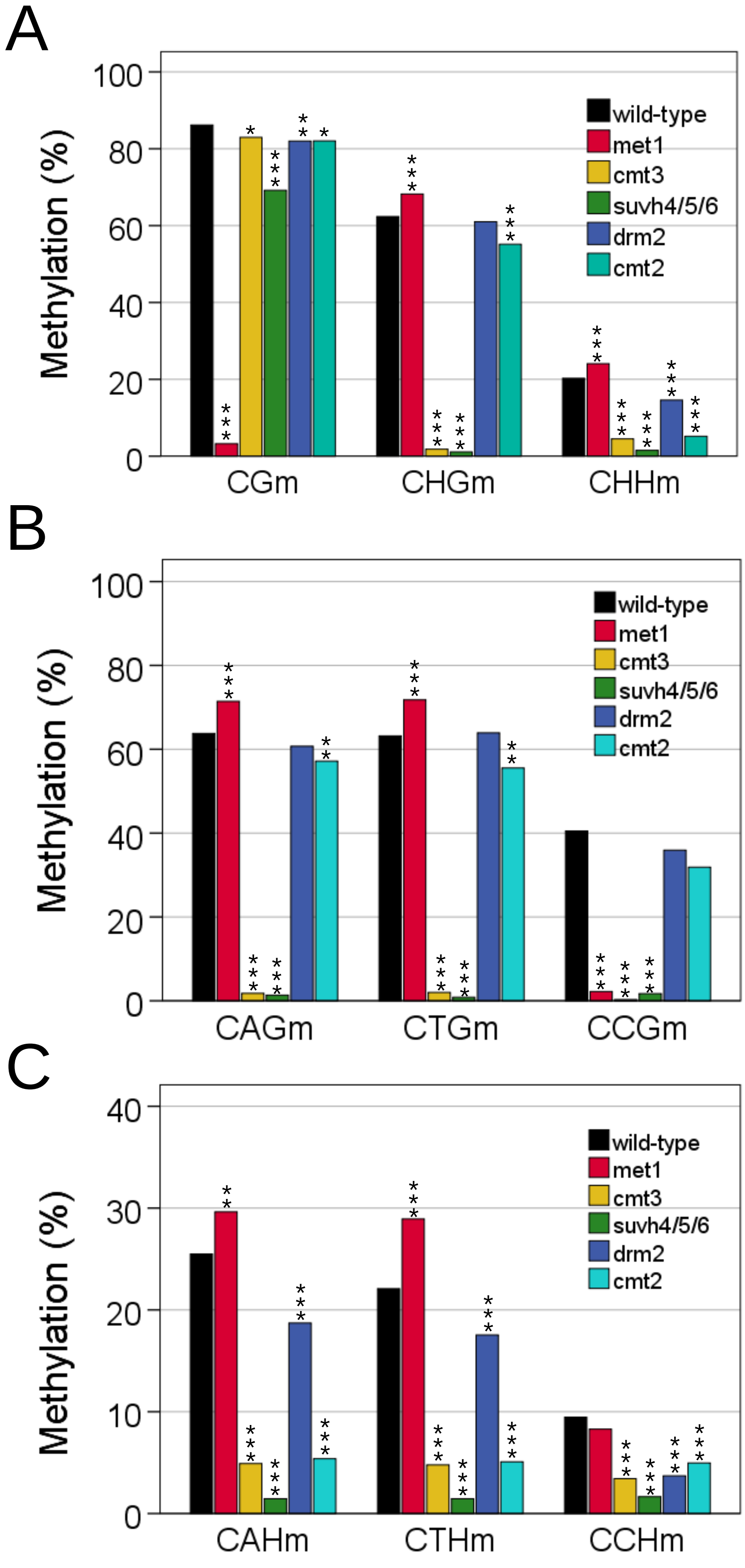

Supplementary Figure S8

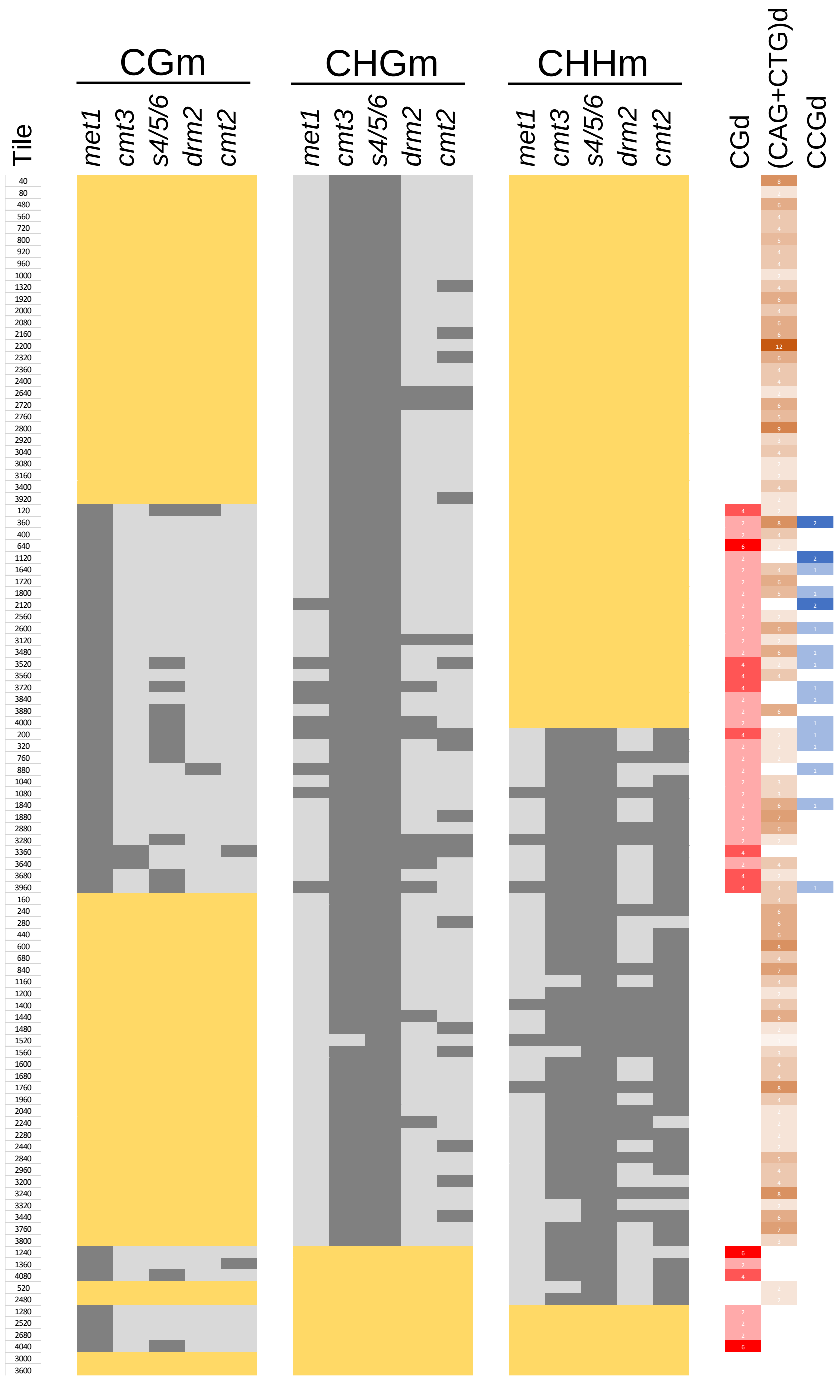

Supplementary Figure S9
