# Supplementary material for: A complex network of interactions governs DNA methylation at telomeric regions
Source: Nucleic Acids Res. 2022 Jan 21;50(3):1449–64. doi: 10.1093/nar/gkac012 (PMC8860613; doi:10.1093/nar/gkac012)
Supplement: gkac012_Supplemental_Files [file gkac012_supplemental_files.zip › Supplementary Figure S4.docx]

**A**

Experiment (Millions of reads) Reads length (bp) *A *B

SRR771524 (106) 100 1.28 15.2

SRR5494752 (29) 101 0.45 9.0

SRR5494753 (32) 101 0.69 16.5

SRR5494754 (40) 101 0.83 16.3

SRR3384734 (34) 151 0.52 9.4

**B**

-SRR771524 (total=3167)

[2685, 316, 66, 31, 12, 13, 4, 1, 2, 4, 0, 3, 1, 1, 3, 0, 1, 0, 0, 0, 0, 1, 2, 0, 1, 0, 0, 0, 0, 0, 0, 0, 0, 0, 2, 0, 0, 0, 0, 0, 0, 1, 4, 2, 11]

-SRR5494752 (total=1059)

[964, 71, 10, 4, 1, 0, 1, 0, 0, 0, 2, 1, 2, 2, 0, 0, 0, 0, 1, 0, 0, 0, 0, 0, 0, 0, 0, 0, 0, 0, 0, 0, 0, 0, 0, 0, 0, 0, 0, 0, 0, 0, 0, 0]

-SRR5494753 (total=916)

[765, 110, 17, 12, 2, 3, 1, 0, 0, 2, 0, 0, 1, 2, 1, 0, 0, 0, 0, 0, 0, 0, 0, 0, 0, 0, 0, 0, 0, 0, 0, 0, 0, 0, 0, 0, 0, 0, 0, 0, 0, 0, 0, 0]

-SRR5494754 (total=729)

[610, 70, 22, 11, 6, 3, 1, 2, 0, 0, 1, 0, 0, 1, 0, 1, 0, 0, 0, 0, 1, 0, 0, 0, 0, 0, 0, 0, 0, 0, 0, 0, 0, 0, 0, 0, 0, 0, 0, 0, 0, 0, 0, 0]

-SRR3384734 (total=53)

[48, 1, 0, 2, 0, 0, 1, 0, 1, 0, 0, 0, 0, 0, 0, 0, 0, 0, 0, 0, 0, 0, 0, 0, 0, 0, 0, 0, 0, 0, 0, 0, 0, 0, 0, 0, 0, 0, 0, 0, 0, 0, 0, 0, 0, 0, 0, 0, 0, 0, 0, 0, 0, 0, 0, 0, 0, 0, 0, 0, 0, 0, 0, 0, 0, 0]

Supplemental Figure S4
