# Supplementary figures and images for: A complex network of interactions governs DNA methylation at telomeric regions
Source: Nucleic Acids Res. 2022 Jan 21;50(3):1449–64. doi: 10.1093/nar/gkac012 (PMC8860613; doi:10.1093/nar/gkac012)

## Slide 1
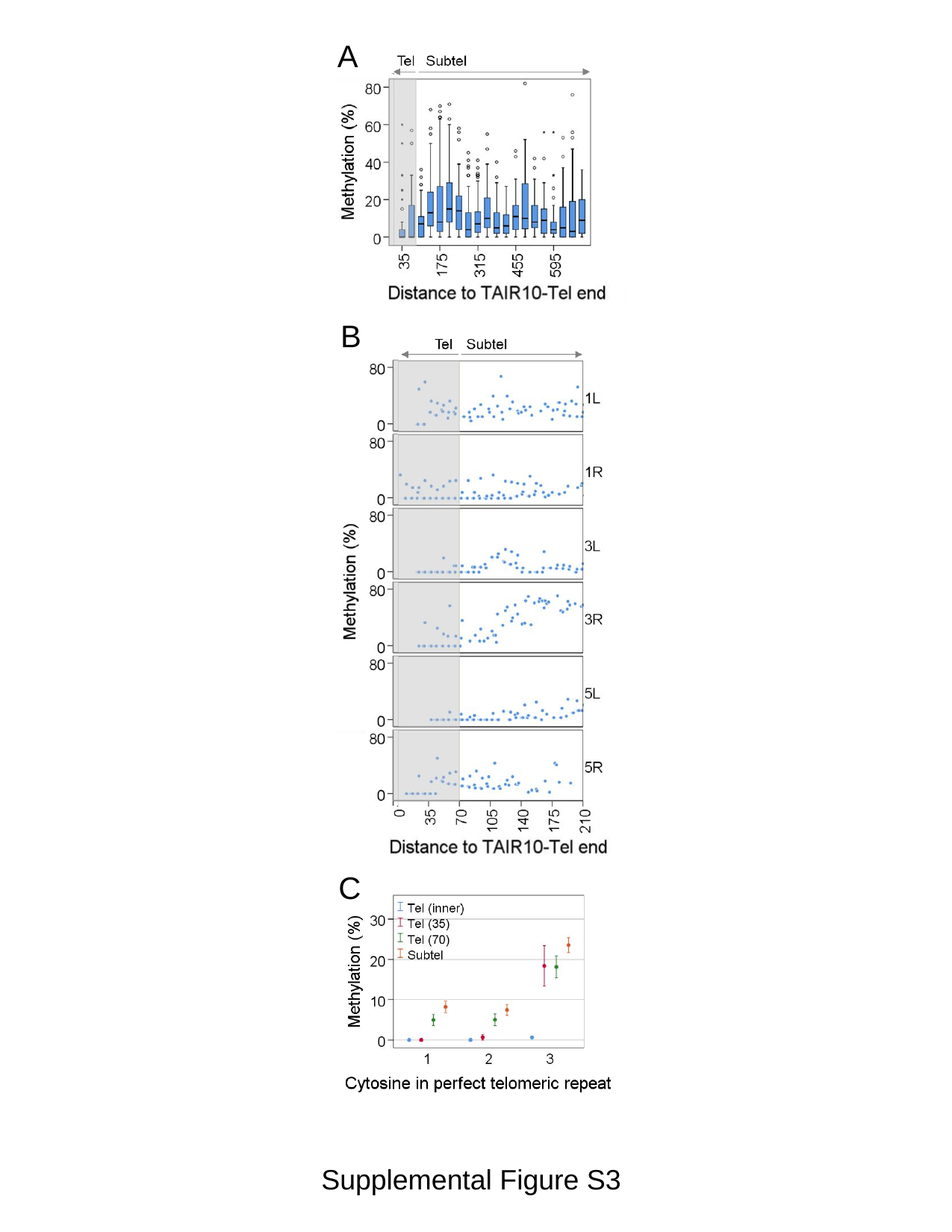

A
B
C
Supplemental Figure S3

Supplement: gkac012_Supplemental_Files [file gkac012_supplemental_files.zip › Supplementary Figure S3.pptx]

## Slide 1
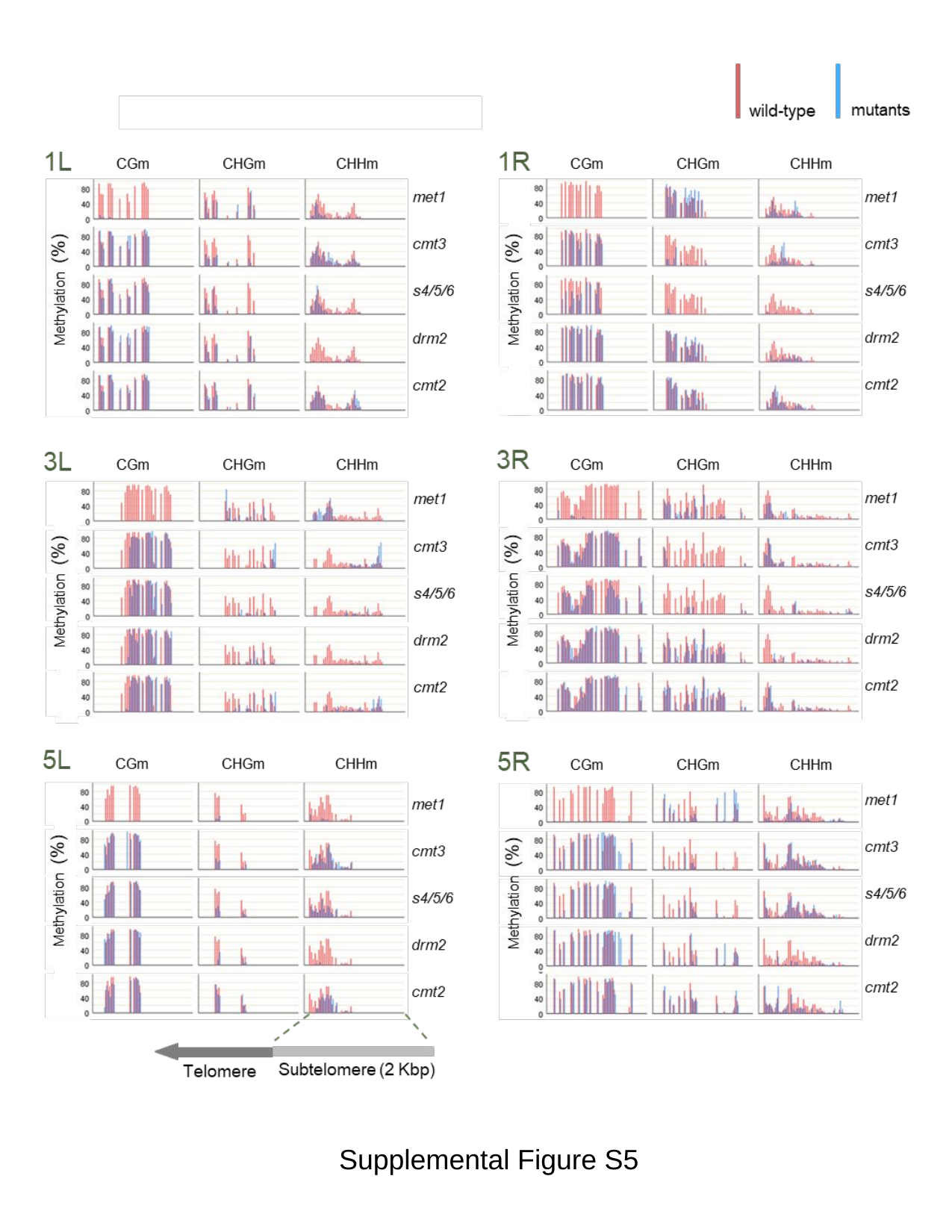

Supplemental Figure S5

Supplement: gkac012_Supplemental_Files [file gkac012_supplemental_files.zip › Supplementary Figure S5.pptx]
